# Parameter redundancy in discrete state‐space and integrated models

**DOI:** 10.1002/bimj.201400239

**Published:** 2016-06-30

**Authors:** Diana J. Cole, Rachel S. McCrea

**Affiliations:** ^1^ School of MathematicsStatistics and Actuarial Science, University of KentCanterburyKent CT2 7NFEngland

**Keywords:** Capture‐recapture, Identifiability, Joint likelihood, Mark‐recovery models, Population dynamics

## Abstract

Discrete state‐space models are used in ecology to describe the dynamics of wild animal populations, with parameters, such as the probability of survival, being of ecological interest. For a particular parametrization of a model it is not always clear which parameters can be estimated. This inability to estimate all parameters is known as parameter redundancy or a model is described as nonidentifiable. In this paper we develop methods that can be used to detect parameter redundancy in discrete state‐space models. An exhaustive summary is a combination of parameters that fully specify a model. To use general methods for detecting parameter redundancy a suitable exhaustive summary is required. This paper proposes two methods for the derivation of an exhaustive summary for discrete state‐space models using discrete analogues of methods for continuous state‐space models. We also demonstrate that combining multiple data sets, through the use of an integrated population model, may result in a model in which all parameters are estimable, even though models fitted to the separate data sets may be parameter redundant.

## 1 Introduction

Sometimes it is not possible to estimate all parameters in a particular parametrization of a model. An obvious example of parameter redundancy occurs when two parameters are confounded, so that they only ever appear as a product. In this case it would only be possible to estimate the product of these two parameters, rather than estimate the parameters individually. Such a model, with a particular parametrization, is termed parameter redundant or the parameters are described as nonidentifiable. While an example where two parameters only ever appear as a product is obviously parameter redundant, in more complex models it will not necessarily be clear whether a particular parametrization of a model is parameter redundant. It is essential to know whether or not a specific parametrization of a model is parameter redundant. This is because a parameter redundant parametrization of a model will have a flat ridge in the likelihood surface (Catchpole and Morgan, 1997), so will not have a unique maximum‐likelihood estimate. Also the standard errors will not exist for all parameters, as the Fisher Information matrix will be singular (Rothenberg, 1971).

Discrete state‐space models are often used to model time series of counts. In ecology, however, they have also been used as a general framework for large classes of models such as capture‐recapture models and occupancy models (see McCrea and Morgan, 2014, chapter 11). Discrete state‐space models are a special case of a wider class of models known as hidden process models (Newman et al., 2006). A discrete state‐space model comprises two stochastic processes: an underlying state equation and an observation equation. The state equation represents the underlying dynamics of how the value of an unobservable state evolves over time. As we cannot necessarily observe all states, the observation equation dictates which components of the system can be measured. The state equation is a stochastic version of widely used matrix projection models (Caswell, 2000). Buckland et al. (2004) describe how the first‐order Markovian transition matrix that describes the dynamics of wild animal populations can be constructed through the partitioning of the individual processes, such as reproduction, survival, and movement. Much work has been carried out recently on state‐space models; see Schaub and Abadi (2011) for a review. At present no methods exist for investigating the parameter redundancy of discrete state‐space models.

General methods have been developed for detecting parameter redundancy (see, e.g., Cole et al., 2010; Choquet and Cole, 2012). These methods involve forming a derivative matrix by differentiating a vector of parameter combinations with respect to the parameters of the model, and then calculating the rank of the resulting matrix. The vector of parameter combinations needs to provide a unique representation of the model. Although such vectors exist for continuous state‐space models (see, e.g., Chis et al., 2011), these are not applicable for discrete state‐space models because the model forms are different.

State‐space models may be parameter redundant because not all states are observed or may be parameter redundant due to the structure of the underlying state equation. However there may still be interest in estimating all the parameters. A possible solution to parameter redundancy could be to combine two or more different types of data, and then describe them with an integrated population model. While the component models may be parameter redundant the combined model may not be parameter redundant (see, e.g., Besbeas et al., 2002). Integrated population models are generally constructed as a product of component likelihoods resulting in a more complex model.

In this paper we demonstrate how to investigate parameter redundancy in discrete state‐space models and integrated population models. In Section 1.1 we introduce the theory of parameter redundancy and methods for detecting parameter redundancy. In Section 1.2 we introduce state‐space models. Sections 2 and 3 extend existing parameter redundancy methods to state‐space models and integrated population models, respectively. Throughout we use simple examples to illustrate the methods; we provide more complex examples in Section 4 and the Supporting Information. Computer code for all examples can also be found in the Supporting Information. The paper concludes with a discussion in Section 5.

### 1.1 Detecting parameter redundancy

A model, M(θ), with parameters given by the vector θ, of length *p*, is defined as parameter redundant if it can be rewritten in terms of a smaller vector of parameters β, of length *q* with q<p and β=f(θ), for some function *f* (Catchpole and Morgan, 1997). A model, M(θ), is said to be nonidentifiable if two different values of the parameters, $\theta_{1}$ and $\theta_{2}$, result in an identical expression of the model, that is, $M\left(\theta_{1}\right)=M\left(\theta_{2}\right)$, but $\theta_{1}\neq \theta_{2}$ (see, e.g., Cole et al., 2010). A parameter redundant model will be nonidentifiable (Catchpole and Morgan, 1997).

Models are described by exhaustive summaries, which are particular parameter combinations that fully specify models. A vector of parameters, κ(θ), is an exhaustive summary if knowledge of κ(θ) uniquely determines the model (Cole et al., 2010). That is, the vector κ(θ) is an exhaustive summary for model M(θ), if $\kappa \left(\theta_{1}\right)=\kappa \left(\theta_{2}\right)\Leftrightarrow M\left(\theta_{1}\right)=M\left(\theta_{2}\right)$ for all $\theta_{1}$ and $\theta_{2}$ in a given parameter space. An exhaustive summary can be used to infer results about parameter redundancy. For example, if the log‐likelihood can be written as $l=\sum_{i=1}^{N}l_{i}$, an exhaustive summary is $\kappa =[l_{1},...,l_{N}]$. However for any example there will be many different options for exhaustive summaries, which we demonstrate in Example 1a.

#### Example 1a

Mark‐recovery data, where animals are marked and dead individuals are subsequently reported, are one type of data that can be combined with census data, in integrated population models (Besbeas et al., 2002). Suppose that $F_{i}$ newborn animals are marked in year *i* and then $N_{ij}$ of those animals are recovered dead in year *j*. For this example we assume that all $N_{ij}$ (for j≥i) and $F_{i}$ are nonzero. Cole et al. (2012) considers relaxing these assumptions.

An example of mark‐recovery data arises from ring‐recovery experiments on birds, where animals are ringed and some of those ringed are recovered dead later (see, e.g., Freeman and Morgan, 1990, 1992). An example of ring‐recovery data is given in Table 1, which is data collected on Lapwing, *Vanellus vanellus*, where birds are ringed in their first year of life. Further years of data are given in Catchpole et al. (1999).

**Table 1:** Example ring‐recovery data on lapwings

Year of ringing	Number marked	Year of recovery		
		1963	1964	1965
1963	1147	13	4	1
1964	1285		16	4
1965	1106			11

We assume that the probability an animal survives a year is ϕ_1_ in the first year of life and $\phi_{a}$ in subsequent years. As animals may die without the marks being recovered, the probability of recovery also needs to be considered, which is λ_1_ in the first year of life and $\lambda_{a}$ in subsequent years. Each year of marking corresponds to a multinomial distribution with probability of marking in year *i* and recovery in year *j* defined by

$$\begin{array}{c} P_{ij}=\left\{\begin{array}{cc} \left(1-\phi_{1}\right)\lambda_{1} & \text{if}\;i=j\\ \phi_{1}\phi_{a}^{j-i-1}\left(1-\phi_{a}\right)\lambda_{a} & \text{otherwise.} \end{array}\right. \end{array}$$

An example where there is interest in this particular model is provided in Robinson (2010). The log‐likelihood for 3 years of marking and 3 years of recovery is

$$\begin{array}{c} l=\sum_{i=1}^{3}\left\{\sum_{j=i}^{3}N_{ij}\log\left(P_{ij}\right)+\left(F_{i}-\sum_{j=i}^{3}N_{ij}\right)\log\left(1-\sum_{j=i}^{3}P_{ij}\right)\right\}, \end{array}$$

where $1-\sum_{j=i}^{3}P_{ij}$ is the probability of being marked but not recovered. Equation (1) shows three exhaustive summaries for this model.

(1) $$\kappa_{1}=\left[\begin{array}{c} N_{11}\log\left\{\left(1-\phi_{1}\right)\lambda_{1}\right\}\\ N_{12}\log\left\{\phi_{1}\left(1-\phi_{a}\right)\lambda_{a}\right\}\\ N_{13}\log\left\{\phi_{1}\phi_{a}\left(1-\phi_{a}\right)\lambda_{a}\right\}\\ \left(F_{1}-\sum_{1}^{3}N_{1j}\right)\log\left(1-\sum_{1}^{3}P_{1j}\right)\\ N_{22}\log\left\{\left(1-\phi_{1}\right)\lambda_{1}\right\}\\ N_{23}\log\left\{\phi_{1}\left(1-\phi_{a}\right)\lambda_{a}\right\}\\ \left(F_{2}-\sum_{2}^{3}N_{2j}\right)\log\left(1-\sum_{2}^{3}P_{2j}\right)\\ N_{33}\log\left\{\left(1-\phi_{1}\right)\lambda_{1}\right\}\\ \left(F_{3}-N_{33}\right)\log\left(1-P_{3j}\right) \end{array}\right],\kappa_{2}=\left[\begin{array}{c} \left(1-\phi_{1}\right)\lambda_{1}\\ \phi_{1}\left(1-\phi_{a}\right)\lambda_{a}\\ \phi_{1}\phi_{a}\left(1-\phi_{a}\right)\lambda_{a}\\ 1-\sum_{1}^{3}P_{1j}\\ \left(1-\phi_{1}\right)\lambda_{1}\\ \phi_{1}\left(1-\phi_{a}\right)\lambda_{a}\\ 1-\sum_{2}^{3}P_{2j}\\ \left(1-\phi_{1}\right)\lambda_{1}\\ 1-P_{3j} \end{array}\right],\kappa_{3}=\left[\begin{array}{c} \left(1-\phi_{1}\right)\lambda_{1}\\ \phi_{1}\left(1-\phi_{a}\right)\lambda_{a}\\ \phi_{1}\phi_{a}\left(1-\phi_{a}\right)\lambda_{a} \end{array}\right].$$

The first exhaustive summary, $\kappa_{1}$, is formed from the log‐likelihood terms. Given that none of the $N_{ij}$ are equal to zero, multiplying by a constant is a one‐to‐one transformation, as is applying the exponential function, which results in exhaustive summary $\kappa_{2}$. Catchpole and Morgan (1997) show that the more complex terms corresponding to being marked but not recovered are not needed because the components are given by other exhaustive summary terms. It is also not necessary to include repeated terms in an exhaustive summary, which results in exhaustive summary $\kappa_{3}$. There are obviously many other one‐to‐one transformations we could apply to create alternative exhaustive summaries.

Any exhaustive summary of the model will contain the same information on the parameter redundancy of that model. In this example let $\left(1-\phi_{1}\right)\lambda_{1}=\beta_{1}$ and $\phi_{1}\lambda_{a}=\beta_{2}$. We can rewrite all three exhaustive summaries for this model in terms of $\beta_{1},\beta_{2}$ and $\phi_{a}$, giving Eq. (2) .

(2) $$\kappa_{1}=\left[\begin{array}{c} N_{11}\log\left(\beta_{1}\right)\\ N_{12}\log\left\{\beta_{2}\left(1-\phi_{a}\right)\right\}\\ N_{13}\log\left\{\beta_{2}\phi_{a}\left(1-\phi_{a}\right)\right\}\\ \left(F_{1}-\sum_{1}^{3}N_{1j}\right)\log\left(1-\sum_{1}^{3}P_{1j}\right)\\ N_{22}\log\left(\beta_{1}\right)\\ N_{23}\log\left\{\beta_{2}\left(1-\phi_{a}\right)\right\}\\ \left(F_{2}-\sum_{2}^{3}N_{2j}\right)\log\left(1-\sum_{2}^{3}P_{2j}\right)\\ N_{33}\log\left(\beta_{1}\right)\\ \left(F_{3}-N_{33}\right)\log\left(1-P_{3j}\right) \end{array}\right],\kappa_{2}=\left[\begin{array}{c} \beta_{1}\\ \beta_{2}\left(1-\phi_{a}\right)\\ \beta_{2}\phi_{2}\left(1-\phi_{a}\right)\\ 1-\sum_{1}^{3}P_{1j}\\ \beta_{1}\\ \beta_{2}\left(1-\phi_{a}\right)\\ 1-\sum_{2}^{3}P_{2j}\\ \beta_{1}\\ 1-P_{3j} \end{array}\right],\kappa_{3}=\left[\begin{array}{c} \beta_{1}\\ \beta_{2}\left(1-\phi_{a}\right)\\ \beta_{2}\phi_{a}\left(1-\phi_{a}\right) \end{array}\right].$$

As we can reparameterize the model in terms of a smaller number of parameters, the original model is parameter redundant. Parameter redundancy in mark‐recovery models is discussed in Cole et al. (2012).□

It is not always obvious whether or not a model can be reparameterized in terms of fewer parameters. In these cases there are several methods for investigating whether or not a model is parameter redundant, which include numerical methods (e.g., Viallefont et al., 1998), symbolic differentiation methods (e.g., Cole et al., 2010), and hybrids of numeric and symbolic methods (Choquet and Cole, 2012).

For the symbolic method a derivative matrix is formed by differentiating an exhaustive summary, κ(θ), with respect to the parameters, θ. The number of estimable parameters in a model is then equal to the rank, *r*, of the derivative matrix, D=∂κ/∂θ. If the rank is less than the number of parameters the model is parameter redundant, otherwise the model is termed full rank, and it is, in theory, possible to estimate all the parameters (Catchpole and Morgan, 1997; Cole et al., 2010). The symbolic algebra of this method can be executed in a symbolic algebra package such as Maple (see, e.g., Catchpole et al., 2002). We provide Maple code for all the examples of this paper in the Supporting Information. This method is demonstrated in the continuation of Example 1a.

#### Example 1a continued

Using the exhaustive summary $\kappa_{3}$ of Eq. (1), which has parameters $\theta =[\phi_{1},\phi_{a},\lambda_{1},\lambda_{a}]$, we form the derivative matrix,

(3) $$D=\left[\frac{\partial \kappa_{3j}}{\partial \theta_{i}}\right]=\left[\begin{array}{ccc} -\lambda_{1} & \left(1-\phi_{a}\right)\lambda_{a} & \phi_{a}\left(1-\phi_{a}\right)\lambda_{a}\\ 0 & -\phi_{1}\lambda_{a} & \phi_{1}\lambda_{a}-2\phi_{1}\phi_{a}\lambda_{a}\\ 1-\phi_{1} & 0 & 0\\ 0 & \phi_{1}\left(1-\phi_{a}\right) & \phi_{1}\phi_{a}\left(1-\phi_{a}\right) \end{array}\right].$$

The rank of this derivative matrix is 3, but there are four parameters so this parametrization of the model is parameter redundant.□

If a particular parametrization of a model is parameter redundant then it is said to have deficiency d=p−r. By examining the null space of D′, where D′ is the transpose of D, it is possible to find parameters that can be estimated. This involves solving $\alpha^{\prime }D=0$. There are *d* solutions to $\alpha^{\prime }D=0$, labeled $\alpha_{j}$, for j=1,...,d, with individual entries $\alpha_{ij}$. If $\alpha_{ij}=0$ for all *j* then it will still be possible to estimate $\theta_{i}$ even though not all parameters can be estimated. In order to find other parameter combinations that can be estimated, we solve the system of linear first‐order partial differential equations (PDEs) $\sum_{i=1}^{p}\alpha_{ij}\partial f/\partial \theta_{i}=0,j=1,...,d$ for arbitrary function *f* (Catchpole et al., 1998; Chappell and Gunn, 1998; Evans and Chappell, 2000; Cole et al., 2010).

#### Example 1a continued

The null space of the derivative matrix given by Eq. (3) is

$$\begin{array}{c} \alpha =\left[-\frac{\phi_{1}}{\lambda_{a}},0,-\frac{\lambda_{1}\phi_{a}}{\left(1-\phi_{1}\right)\lambda_{a}},1\right]. \end{array}$$

The zero in the second position indicates that only the parameter $\phi_{a}$ can be estimated in the original parametrization. Solving the PDEs,

$$\begin{array}{c} -\frac{\partial f}{\partial \phi_{1}}\frac{\phi_{1}}{\lambda_{a}}-\frac{\partial f}{\partial \lambda_{1}}\frac{\lambda_{1}\phi_{a}}{\left(1-\phi_{1}\right)\lambda_{a}}+\frac{\partial f}{\partial \lambda_{a}}=0, \end{array}$$

gives estimable parameter combinations $\phi_{1}\lambda_{a}$ and $\left(1-\phi_{1}\right)\lambda_{1}$.□

We show in the Maple code for Example 1a that all the exhaustive summaries of Eq. (2) result in derivative matrices that all have rank 3 and the same null space, resulting in identical estimable parameter combinations. This is true of all exhaustive summaries for a model. Although all three exhaustive summaries give the same results, in this case we would recommend using the third exhaustive summary, $\kappa_{3}$, as it results in the simplest derivative matrix. Generally the best exhaustive summary to use is one that results in the simplest derivative matrix; this is often also the exhaustive summary with the smallest dimension.

There are alternative methods of investigating parameter redundancy, which are summarized in Gimenez et al. (2004). However numeric methods, such as examining the rank of the Hessian matrix (Viallefont et al., 1998), can lead to incorrect results (see, e.g., Cole and Morgan, 2010a). While the symbolic method is preferred, because it gives correct results, in more complex problems Maple may run out of memory trying to calculate the rank of the derivative matrix. Examples of models where symbolic computations would be too difficult include Hunter and Caswell (2009), Jiang et al. (2007), and Forcina (2008). In these three papers numerical methods are used to detect parameter redundancy. However, it is still possible to apply the symbolic approach to these more complicated models by using a framework provided in Cole et al. (2010). This involves creating new structurally simpler exhaustive summaries by reparameterizing the model in such a way that the resulting derivative matrix is structurally simpler. The Reparametrization theorem states that if s is a reparametrization of a model parameterized in terms of θ such that $\text{rank}\left(\partial s/\partial \theta \right)=n_{s}$, where $n_{s}$ is the length of s, then rank(D)=rank{∂κ(θ)/∂θ}=rank{∂κ(s)/∂s}. The s can be chosen so that the derivative matrix, ∂κ(s)/∂s, is structurally simpler than the derivative for the original parametrization, ∂κ(θ)/∂θ, and therefore the rank can be calculated. The vector s is either a new exhaustive summary, or a new exhaustive summary can be created from s. This extended symbolic method is used in Cole (2012) for models of Hunter and Caswell (2009) and in Cole and Morgan (2010a) for the models of Jiang et al. (2007). In Cole and Morgan (2010b) further theory is developed specifically for models with covariates, with one illustrative example covering the models of Forcina (2008).

An alternative method is the hybrid symbolic‐numeric method (Choquet and Cole, 2012). In this method D is calculated symbolically but the rank is evaluated numerically at five randomly chosen points in the parameter space. In a parameter redundant model it is then possible to determine if any of the original parameters can still be estimated, but it is not possible to find other estimable parameter combinations.

#### Example 1a continued

It is shown in the Maple code for Example 1a that the hybrid symbolic‐numeric method also results in rank 3, and that $\phi_{a}$ can be estimated. However the hybrid method cannot be used to show that the estimable parameter combinations are $\phi_{1}\lambda_{a}$ and $\left(1-\phi_{1}\right)\lambda_{1}$.□

### 1.2 The state‐space model

A continuous linear state‐space model is defined by the equations,

$$\begin{array}{c} y\left(t,\theta \right)=C\left(\theta \right)x\left(t,\theta \right)\;\text{and}\;\frac{\partial }{\partial t}x\left(t,\theta \right)=A\left(\theta \right)x\left(t,\theta \right)+B\left(\theta \right)u, \end{array}$$

where y is the output function, x is the state‐variable function, θ is a vector of unknown parameters, u is the input function, and *t* is the time recorded on a continuous scale. The matrices A, B, C are the compartmental, input, and the output matrices, respectively. Several exhaustive summaries already exist for continuous linear state‐space models, which result in different general methods for detecting nonidentifiability including the Laplace transform approach (Bellman and Aström, 1970) and the Taylor series approach (Pohjanpalo, 1978). These different methods are compared in Chis et al. (2011). However, these exhaustive summaries are not suitable for discrete linear state‐space models, as the model formation is different. Discrete state‐space models do not involve differential equations, but instead use a recursion such as Eq. (4) .

In ecology, observations are typically recorded on a discrete scale, such as counts of population sizes. A discrete linear state‐space model is defined by the respective observation and state equations,

(4) $$y_{t}=A_{t}x_{t}+\eta_{t}\;\text{and}\;x_{t}=C_{t}x_{t-1}+\varepsilon_{t-1}\text{,}\;t=1,2,3,...,$$

where A_*t*_ is an n×n transition matrix, C_*t*_ is an m×n measurement matrix, $x_{0}$ is a vector of initial values, and η and ε are error processes with zero means. The model has *n* states, with *m* states or combination of states observed, where m≤n. It is assumed that the initial observation is at time t=1 and the final observation is at t=T. For ecological applications, observations are typically taken on a yearly time scale, and the matrix A_*t*_ will contain the demographic parameters of interest, such as survival probabilities and fecundity. Typically the measurement matrix is a fixed matrix that does not contain any parameters and does not depend on time, so that $C_{t}=C$. A linear Gaussian state‐space model assumes that the error processes $\eta_{t}$ and $\varepsilon_{t}$ and the initial state $x_{0}$ have multivariate normal distributions, with all three variables being independent between times and each other. Assuming a linear Gaussian state‐space model allows parameters to be estimated using the Kalman filter (see, e.g., Harvey, 1989), but this assumption is not necessary to check whether or not a model is parameter redundant.

#### Example 1b

An example of a simple discrete state‐space model used in ecology is given in Besbeas et al. (2002). They create a discrete linear state‐space model for abundance data on the number of lapwings, *V. vanellus*, with the respective observation and state equations:

$$\begin{array}{c} y_{t}=\left[\begin{array}{cc} 0 & 1 \end{array}\right]x_{t}+\eta_{t}\;\text{with}\;x_{t}=\left[\begin{array}{cc} 0 & \rho \phi_{1}\\ \phi_{a} & \phi_{a} \end{array}\right]x_{t-1}+\left[\begin{array}{c} \varepsilon_{1,t}\\ \varepsilon_{a,t} \end{array}\right]. \end{array}$$

Here, $x_{t}={\left[N_{1,t},N_{a,t}\right]}^{\prime }$, where $N_{1,t}$ and $N_{a,t}$ are, respectively, the number of first year lapwings and adult lapwings seen in year $t,y_{t}$ is the observed numbers of lapwings at time t,ρ is a parameter representing productivity, and ϕ_1_ and $\phi_{a}$ are the probabilities of survival for first year and adult lapwings, respectively. The error term $\eta_{1,t}$ is assumed to follow a normal distribution with mean zero and variance σ^2^, and the error terms $\varepsilon_{1,t}$ and $\varepsilon_{a,t}$ are also assumed initially to have mean zero. This assumption is used in Besbeas et al. (2002) to allow the use of the Kalman filter in estimating parameters. For simplicity in the example here, we assume that the variance in the error terms is known. In Supporting Information Appendix A this assumption is relaxed.

This parametrization of the model is obviously parameter redundant due to the confounding of the parameters ρ and ϕ_1_ and would remain parameter redundant even if numbers of first year lapwings were observed. However we use this model to illustrate the methods in this paper because it is simple enough that derivative matrices and PDEs can be displayed. In Section 4 we give examples of more complex models, where parameter redundancy results are not obvious, and the methods of this paper are needed in order to investigate parameter redundancy.□

An extension to the state‐space model allows nonlinear functions with

(5) $$y_{t}=h\left(x_{t},\theta \right)+\eta_{t}\;\text{with}\;x_{t}=g\left(x_{t-1},\theta \right)+\varepsilon_{t-1}\text{,}\;t=1,2,3,...,$$

where $\eta_{t}$ and $\varepsilon_{t}$ are error processes, $x_{0}$ is a vector of initial values, and h(·) and g(·) are known functions.

## 2 Discrete state‐space model exhaustive summaries

To examine the parameter redundancy of a discrete state‐space model, we need a suitable exhaustive summary. Several exhaustive summaries exist for examining the identifiability of compartment models, which are continuous state‐space models (see, e.g., Chis et al., 2011). These exhaustive summaries include the Laplace transform approach (Bellman and Aström, 1970) and the Taylor series approach (Pohjanpalo, 1978).

The Laplace transform approach, which is described in Supporting Information Appendix A, involves taking the Laplace transform of y(t). The discrete analogue of a Laplace transform is the *z*‐transform; we show how this can be used to derive an exhaustive summary in Supporting Information Appendix A. The *z*‐transform exhaustive summary is given in Theorem 2.1 a. Like its continuous analogue, this approach is only applicable for linear discrete state‐space models without time‐dependent parameters.

The Taylor series approach involves a Taylor series expansion of $y_{t}$. A similar idea can be utilized for discrete state‐space models, which involves a direct expansion of $y_{t}$ starting at $y_{0}$. This results in the expansion exhaustive summary given in Theorem 2.1 b. The proof for this method is given in Supporting Information Appendix A. The expansion exhaustive summary, like its continuous analogue, can be used for linear and nonlinear discrete state‐space models.

Theorem 2.1 **Exhaustive Summaries for discrete state‐space models**

a. For discrete linear state‐space models with nontime‐dependent transition matrix, A, and measurement matrix, C, the z‐transform exhaustive summary is formed from the nonconstant coefficients of the powers of *z* in the numerator and denominator of the transfer function,
  $$\begin{array}{c} Q\left(z\right)=C{\left(zI-A\right)}^{-1}Ax_{0}, \end{array}$$
  where I is the identity matrix with dimensions identical to A.
b. The expansion exhaustive summary for any discrete linear state‐space models is,
  $$\begin{array}{c} \kappa =\left[\begin{array}{c} C_{1}A_{1}x_{0}\\ C_{2}A_{2}A_{1}x_{0}\\ C_{3}A_{3}A_{2}A_{1}x_{0}\\ \vdots \end{array}\right]. \end{array}$$
  and for nonlinear state‐space models is,
  $$\begin{array}{c} \kappa =\left[\begin{array}{c} h\{g\left(x_{0}\right)\}\\ h[g\left\{g\left(x_{0}\right)\right\}]\\ h(g\left[g\left\{g\left(x_{0}\right)\right\}\right])\\ \vdots \end{array}\right]=\left[\begin{array}{c} h\{g\left(x_{0}\right)\}\\ h\{g^{2}\left(x_{0}\right)\}\\ h\{g^{3}\left(x_{0}\right)\}\\ \vdots \end{array}\right]. \end{array}$$

#### Example 1b continued

In Example 1b,

$$\begin{array}{c} C=\left[\begin{array}{cc} 0 & 1 \end{array}\right]\text{,}\;A=\left[\begin{array}{cc} 0 & \rho \phi_{1}\\ \phi_{a} & \phi_{a} \end{array}\right],\;\text{and}\;x_{0}=\left[\begin{array}{c} x_{0,1}\\ x_{0,2} \end{array}\right]. \end{array}$$

The transfer function is,

$$\begin{array}{c} Q\left(z\right)=C{\left(zI-A\right)}^{-1}Ax_{0}=\frac{-z\phi_{a}\left(x_{0,1}+x_{0,2}\right)-\rho \phi_{1}\phi_{a}x_{0,2}}{-s^{2}+z\phi_{a}+\rho \phi_{1}\phi_{a}}. \end{array}$$

The *z*‐transform exhaustive summary is then the nonzero coefficients of *z* in the numerator and denominator. This results in the exhaustive summary,

(6) $$\kappa =\left[\begin{array}{c} -\rho \phi_{1}\phi_{a}x_{0,2}\\ -\phi_{a}\left(x_{0,1}+x_{0,2}\right)\\ \rho \phi_{1}\phi_{a}\\ \phi_{a} \end{array}\right].$$

Differentiating the exhaustive summary with respect to the parameters, $\theta =[\phi_{1},\phi_{a},\rho ]$, gives the derivative matrix,

$$\begin{array}{c} D=\left[\begin{array}{cccc} -\rho \phi_{a}x_{0,2} & 0 & \rho \phi_{a} & 0\\ -\rho \phi_{1}x_{0,2} & -x_{0,1}-x_{0,2} & \rho \phi_{1} & 1\\ -\phi_{1}\phi_{a}x_{0,2} & 0 & \phi_{1}\phi_{a} & 0 \end{array}\right], \end{array}$$

which has rank 2. Therefore this model is parameter redundant with deficiency 1. The null space of D′ is $\alpha^{\prime }=\left[-\phi_{1}/\rho ,0,1\right]$. The position of the zero indicates that the second parameter $\phi_{a}$ can be estimated. Solving the PDEs $-\partial f/\partial \phi_{1}\times \phi_{1}/\rho +\partial f/\partial \rho =0$ gives $\phi_{1}\rho$ as the other estimable parameter.

The expansion exhaustive summary for this example consists of the terms,

$$\begin{array}{c} \kappa =\left[\begin{array}{c} C_{1}A_{1}x_{0}\\ C_{2}A_{2}A_{1}x_{0}\\ C_{3}A_{3}A_{2}A_{1}x_{0}\\ \vdots \end{array}\right]=\left[\begin{array}{c} x_{0,1}\phi_{a}+x_{0,2}\phi_{a}\\ x_{0,2}\phi_{1}\phi_{a}\rho +x_{0,1}\phi_{a}^{2}+x_{0,2}\phi_{a}^{2}\\ x_{0,1}\phi_{1}\phi_{a}^{2}\rho +2x_{0,2}\phi_{1}\phi_{a}^{2}\rho +x_{0,1}\phi_{a}^{3}+x_{0,2}\phi_{a}^{3}\\ \vdots \end{array}\right]. \end{array}$$

However to use this expansion exhaustive summary in Maple we need to fix the number of terms.□

To use the expansion exhaustive summary there are three possible options:

Option I: If there are *T* years of data, the exhaustive summary is $\kappa^{\prime }=\left[{\left(C_{1}A_{1}x_{0}\right)}^{\prime },{\left(C_{2}A_{2}A_{1}x_{0}\right)}^{\prime },...,{\left(C_{T}A_{T}\cdots A_{2}A_{1}x_{0}\right)}^{\prime }\right]$ for a discrete linear state‐space model or $\kappa^{\prime }=[h{\left\{g\left(x_{0}\right)\right\}}^{\prime },h{\left\{g^{2}\left(x_{0}\right)\right\}}^{\prime },...,$ $h{\left\{g^{T}\left(x_{0}\right)\right\}}^{\prime }]$ for a discrete non‐inear state‐space model.
Option II: Theorem 2.2 provides a limit to the number of exhaustive summary terms required.
Option III: The Extension theorem of Catchpole and Morgan (1997) and Cole et al. (2010) can be used.

Option I is straightforward. However note that in Example 1b each successive exhaustive summary term is more complex than the previous. It will always be the case due to the nature of this exhaustive summary. Therefore Option I is not suitable for large studies, when exhaustive summary terms become too complex. In such cases Options II or III should be used instead.

Option II is based on a result for continuous state‐space models. The Taylor series expansion method creates an exhaustive summary that is infinite. For a linear system of compartment models with *n* compartments, only the first (2n−1) terms are required (Thowsen, 1978; Magaria et al., 2001). Theorem 2.2 provides a similar result.

Theorem 2.2 If A and C are constant and there are *n* states, then a simpler exhaustive summary requires only the terms $\kappa ={\left[E{\left(y_{1}\right)}^{\prime },E{\left(y_{2}\right)}^{\prime },...,E{\left(y_{2n}\right)}^{\prime }\right]}^{\prime }$.

The proof of Theorem 2.2 is given in Supporting Information Appendix A.

To use Option III we consider the exhaustive summary $\kappa_{K}$, for the first *K* years, which is a function of parameters $\theta_{K}$. Suppose the derivative matrix $D_{K}=\partial \kappa_{K}/\partial \theta_{K}$ is full rank. The exhaustive summary can be extended to $\kappa_{K+1}$ with extra terms $\kappa_{ex}$ and parameters $\theta_{K+1}=\left[\theta_{K},\theta_{ex}\right]$. If $D_{ex}=\partial \kappa_{ex}/\partial \theta_{ex}$ is full rank, then by the Extension theorem the model will be full rank for T≥K (Catchpole and Morgan, 1997; Cole et al., 2010). If a model is parameter redundant, with rank q<p, the model can be reparameterized in terms of a vector of parameters, β, of length *q*. The vector β can be the estimable parameter combinations found by solving the PDEs described in Section 1.1. Once the model has been reparameterized, the Extension theorem can be applied with derivative matrices formed by differentiating the exhaustive summary with respect to β rather than θ. The Reparametrization theorem of Cole et al. (2010) can be used to give the result that the model specified in terms of θ has the same rank as the model reparameterized in terms of β.

#### Example 1b continued

As n=2, the exhaustive summary using Option II is,

(7) $$\begin{array}{ccc} \kappa_{1} & = & \left[x_{0,1}\phi_{a}+x_{0,2}\phi_{a},x_{0,2}\phi_{1}\phi_{a}\rho +x_{0,1}\phi_{a}^{2}+x_{0,2}\phi_{a}^{2},x_{0,1}\phi_{1}\phi_{a}^{2}\rho +2x_{0,2}\phi_{1}\phi_{a}^{2}\rho +x_{0,1}\phi_{a}^{3}+\right.\\ & & {\left.+\;x_{0,2}\phi_{a}^{3},x_{0,2}\phi_{1}^{2}\phi_{a}^{2}\rho^{2}+2x_{0,1}\phi_{1}\phi_{a}^{3}\rho +3x_{0,2}\phi_{1}\phi_{a}^{3}\rho +x_{0,1}\phi_{a}^{4}+x_{0,2}\phi_{a}^{4}\right]}^{\prime }. \end{array}$$

The derivative matrix, formed with respect to the parameters $\theta =[\phi_{1},\phi_{a},\rho ]$, is,

(8) $$D=\frac{\partial \kappa_{1}}{\partial \theta }=\left[\begin{array}{cccc} 0 & x_{0,2}\phi_{a}\rho & x_{0,1}\phi_{a}^{2}\rho +2x_{0,2}\phi_{a}^{2}\rho & d_{1,4}\\ x_{0,1}+x_{0,2} & x_{0,2}\phi_{1}\rho +2x_{0,1}\phi_{a}+2x_{0,2}\phi_{a} & d_{2,3} & d_{2,4}\\ 0 & x_{0,2}\phi_{1}\phi_{a} & x_{0,1}\phi_{1}\phi_{a}^{2}+2x_{0,2}\phi_{1}\phi_{a}^{2} & d_{3,4} \end{array}\right],$$

where $d_{1,4}=2x_{0,2}\phi_{1}\phi_{a}^{2}\rho^{2}+2x_{0,1}\phi_{a}^{3}\rho +3x_{0,2}\phi_{a}^{3}\rho ,d_{2,3}=2x_{0,1}\phi_{1}\phi_{a}\rho +4x_{0,1}\phi_{1}\phi_{a}\rho +3x_{0,1}\phi_{a}^{2}+3x_{0,2}\phi_{a}^{2},d_{2,4}=2x_{0,2}\phi_{1}^{2}\phi_{a}\rho^{2}+6x_{0,1}\phi_{1}\phi_{a}^{2}\rho +9x_{0,2}\phi_{1}\phi_{a}^{2}\rho +4x_{0,1}\phi_{a}^{3}+4x_{0,2}\phi_{a}^{3}$, and $d_{3,4}=2x_{0,2}\phi_{1}^{2}\phi_{a}^{2}\rho +2x_{0,1}\phi_{1}\phi_{a}^{3}+3x_{0,2}\phi_{1}\phi_{a}^{3}$. The rank of this derivative matrix is 2. As there are three parameters in this model, the model is parameter redundant with deficiency 1. The null space is identical to using the *z*‐transform exhaustive summary, resulting in the same conclusion that the estimable parameter combinations are $\phi_{a}$ and $\phi_{a}\rho$. Alternatively we show how Option III can be used in the Maple code. □

If there are time‐dependent A_*t*_ and/or C_*t*_, then the *z*‐transform exhaustive summary and Theorem 2.2 do not apply. However the expansion exhaustive summary can be used with Option I or Option III, the latter of which we demonstrate in the example below.

#### Example 2

Consider Example 1b extended to include time‐dependent first year survival, so that the transition matrix is $A_{t}=\left[\begin{array}{cc} 0 & \rho \phi_{1,t}\\ \phi_{a} & \phi_{a} \end{array}\right]$. The expansion exhaustive summary for T=3 is $\kappa_{3}\left(\theta \right)={\left[\phi_{a}x_{0,1}+\phi_{a}x_{0,2},x_{0,2}\phi_{1,1}\phi_{a}\rho +x_{0,1}\phi_{a}^{2}+x_{0,2}\phi_{a}^{2},x_{0,1}\phi_{1,2}\phi_{a}^{2}\rho +x_{0,2}\phi_{1,2}\phi_{a}^{2}\rho +x_{0,2}\phi_{1,1}\phi_{a}^{2}\rho +x_{0,1}\phi_{a}^{3}+x_{0,2}\phi_{a}^{3}\right]}^{\prime }$. Differentiating this exhaustive summary with respect to the parameters, $\theta_{3}=\left[\rho ,\phi_{a},\phi_{1,1},\phi_{1,2}\right]$ results in the derivative matrix,

$$\begin{array}{c} \frac{\partial \kappa_{3}}{\partial \theta_{3}}\;=\;\left[\;\begin{array}{ccc} 0 & \phi_{1,1}\phi_{a}x_{0,2} & \phi_{a}^{2}\left(\phi_{1,1}x_{0,2}\;+\;\phi_{1,2}x_{0,1}\;+\;\phi_{a}^{2}\phi_{1,2}x_{0,2}\right)\\ x_{0,1}\;+\;x_{0,2} & \rho \phi_{1,1}x_{0,2}\;+\;2\phi_{a}\left(x_{0,1}\;+\;x_{0,2}\right) & 2\rho \phi_{a}\left\{\phi_{1,1}x_{0,2}\;+\;\phi_{1,2}\left(x_{0,1}\;+\;x_{0,2}\right)\right\}\;+\;3\phi_{a}^{2}\left(x_{0,1}\;+\;x_{0,2}\right)\\ 0 & \rho \phi_{a}x_{0,2} & \rho \phi_{a}^{2}x_{0,2}\\ 0 & 0 & \rho \phi_{a}^{2}\left(x_{0,1}\;+\;x_{0.2}\right) \end{array}\;\right], \end{array}$$

which has rank 3. By solving the appropriate set of PDEs in the Maple code, we show that the estimable parameter combinations are $\phi_{a},\phi_{1,1}\rho$, and $\phi_{1,2}\rho$. To use Option III, as this model is parameter redundant, we reparameterize in terms of the estimable parameter combinations to give parameters $\beta_{3}=\left[\phi_{a},\nu_{1},\nu_{2}\right]$ with $\nu_{t}=\phi_{1,t}\rho$, so that $\kappa_{3}\left(\beta \right)={\left[\phi_{a}x_{0,1}+\phi_{a}x_{0,2},x_{0,2}\nu_{1}\phi_{a}+x_{0,1}\phi_{a}^{2}+x_{0,2}\phi_{a}^{2},x_{0,1}\nu_{2}\phi_{a}^{2}+x_{0,2}\nu_{2}\phi_{a}^{2}+x_{0,2}\nu_{1}\phi_{a}^{2}+x_{0,1}\phi_{a}^{3}+x_{0,2}\phi_{a}^{3}\right]}^{\prime }$. The derivative matrix,

$$\begin{array}{c} \frac{\partial \kappa_{3}}{\partial \beta_{3}}=\left[\;\begin{array}{ccc} x_{0,1}\;+\;x_{0,2} & 2x_{0,1}\phi_{a}\;+\;x_{0,2}\nu_{1}\;+\;2x_{0,2}\phi_{a} & 2x_{0,1}\nu_{2}\phi_{a}\;+\;3x_{0,1}\phi_{a}^{2}\;+\;2x_{0,2}\mu_{1}\phi_{a}\;+\;2x_{0,2}\nu_{2}\phi_{a}\;+\;3x_{0,2}\phi_{a}^{2}\\ 0 & x_{0,2}\phi_{a} & x_{0,2}\phi_{a}^{2}\\ 0 & 0 & x_{0,1}\phi_{a}^{2}\;+\;x_{0,2}\phi_{a}^{2} \end{array}\;\right], \end{array}$$

has full rank 3. Increasing *T* to 4 adds only one extra parameter, $\nu_{3}=\phi_{1,3}\rho$, so that $\beta_{ex}=\left[\nu_{3}\right]$ and adds one extra exhaustive summary term $\kappa_{ex}=x_{0,2}\nu_{1}\nu_{3}\phi_{a}^{2}+x_{0,2}\nu_{1}\phi_{a}^{3}+x_{0,1}\nu_{2}\phi_{a}^{3}+x_{0,2}\nu_{2}\phi_{a}^{3}+x_{0,1}\nu_{3}\phi_{a}^{3}+x_{0,2}\nu_{3}\phi_{a}^{3}+x_{0,1}\phi_{a}^{4}+x_{0,2}\phi_{a}^{4}$. The extra part of the derivative matrix,

$$\begin{array}{c} D_{ex}=\frac{\partial \kappa_{ex}}{\partial \beta_{ex}}=\left[\begin{array}{c} x_{0,2}\nu_{1}\phi_{a}^{2}+x_{0,1}\phi_{a}^{3}+x_{0,2}\phi_{a}^{3} \end{array}\right], \end{array}$$

obviously has full rank 1. Therefore by the Extension theorem the model parameterized in terms of β will always have full rank *T* for any T≥3. Then by the Reparametrization theorem the model parameterized in terms of θ will also have rank *T*, but as there are T+1 parameters the model is parameter redundant with deficiency 1. The estimable parameter combinations are then $\phi_{a},\phi_{1,1}\rho ,\phi_{1,2}\rho ,...,\phi_{1,T}\rho$.□

We demonstrate how the expansion exhaustive summary can be used in nonlinear models in Example 3.

#### Example 3

A model for density dependence has the form $x_{t}=x_{t-1}\exp\left\{a+b\log\left(x_{t-1}\right)\right\}$ with $y_{t}=x_{t}$, where $y_{t}$ is the population abundance and *a* and *b* are constant parameters. If we want to estimate *x*_0_, the vector of parameters is $\theta =[x_{0},a,b]$ (see, e.g., Dennis et al., 2006). The exhaustive summary for T=3 is

$$\begin{array}{c} \kappa_{2}=\left[\begin{array}{c} \kappa_{21}\\ \kappa_{22}\\ \kappa_{23} \end{array}\right]=\left[\begin{array}{c} x_{0}\exp\left\{a+b\log\left(x_{0}\right)\right\}\\ x_{0}\exp\left\{a+b\log\left(x_{0}\right)\right\}\exp\left\{a+b\log\left(c_{1}\left[\exp\left\{a+b\log\left(c_{1}\right)\right\}\right]\right)\right\}\\ \kappa_{22}\exp\left\{a+b\log\left(\kappa_{22}\right)\right\} \end{array}\right]. \end{array}$$

The derivative matrix D=∂κ/∂θ has full rank 3. Increasing *T* by 1 adds no extra parameters; in such cases the Extension theorem tells us that the model will always be full rank, without the need to check the rank of another matrix (Remark 7, Catchpole and Morgan, 1997). Therefore we conclude this model is not parameter redundant for any T≥3.□

Supporting Information Appendix A also provides an explanation of how parameters in the error terms can be incorporated into the exhaustive summary.

## 3 Integrated models

If state‐space models are parameter redundant there may still be interest in estimating all of the parameters because of their biological importance. To estimate such parameters one method is to combine two or more different data sets. If the data sets are independent, a joint likelihood can be formed as the product of the individual model likelihoods (see, e.g., Lebreton et al., 1995; Besbeas et al., 2002; McCrea et al., 2013).

An integrated model can be used to describe two or more independent data sets. Each data set can be modeled separately, however some of the models may have parameters in common. If using maximum likelihood to estimate the parameters, and independence of the data sets is assumed, the joint log‐likelihood is $l=\sum_{i=1}^{N}l_{i}$, where there are *N* data sets each with log‐likelihood $l_{i}$. Although structurally simplistic, in terms of addition of two or more log‐likelihood functions, the integrated population model has advanced the modeling of ecological data (see, e.g., chapter 9, Newman et al., 2006 or chapters 11 and 12 of McCrea and Morgan, 2014). We demonstrate that aside from the natural advantages of integrated population models, such as improved precision of parameter estimates and reduced correlation between parameters, integrated population models have the additional advantage of making it possible to estimate some parameters that were not estimable from modeling the data sets individually. It is essential that the tools are made available to be able to understand and identify the parameter redundancy of integrated population models.

In this section we consider two different methods for determining whether or not an integrated model is parameter redundant. The first method is the obvious extension of combining the two exhaustive summaries for each model; this is described in Section 3.1. The second method is an extension of results from Cole et al. (2010) and is of particular use in more complex models; this is described in Section 3.2. The methods are illustrated using the example below.

#### Example 1c

In Besbeas et al. (2002), the state‐space model is combined with a mark‐recovery model. In this example we consider combining both Examples 1a and 1b, as well as the example in Besbeas et al. (2002) where in the ring‐recovery model the reporting probability is not dependent on age so that $\lambda_{1}=\lambda_{a}$.□

### 3.1 Method A: combined exhaustive summary

Suppose there are *N* different data sets being described by an integrated model. The exhaustive summaries for these different data sets are $\kappa_{1},\kappa_{2},...,\kappa_{N}$, with parameters, $\theta_{1},\theta_{2},...,\theta_{N}$. The exhaustive summary for the integrated model is $\kappa ={\left[\kappa_{1}^{\prime },\kappa_{2}^{\prime },...,\kappa_{N}^{\prime }\right]}^{\prime }$ and its parameters are $\theta =[\theta_{1},\theta_{2},...,\theta_{N}]$, with any duplicate parameters removed. The standard derivative matrix method can then be used as explained in Section 1.1.

#### Example 1c continued

This example has N=2 data sets and we use the *z*‐transform exhaustive summary, Eq. (6) for the state‐space part, combined with the third exhaustive summary from Eq. (1) for the mark‐recovery part. This results in a combined exhaustive summary of

$$\begin{array}{c} \kappa =\left[\begin{array}{c} -\rho \phi_{1}\phi_{a}x_{0,2}\\ -\phi_{a}\left(x_{0,1}+x_{0,2}\right)\\ \rho \phi_{1}\phi_{a}\\ \phi_{a}\left(1-\phi_{1}\right)\lambda_{1}\\ \phi_{1}\left(1-\phi_{a}\right)\lambda_{a}\\ \phi_{1}\phi_{a}\left(1-\phi_{a}\right)\lambda_{a} \end{array}\right]. \end{array}$$

The parameters are $\theta =[\phi_{1},\phi_{a},\rho ,\lambda_{1},\lambda_{a}]$. The derivative matrix,

$$\begin{array}{c} D=\frac{\partial \kappa }{\partial \theta }=\left[\begin{array}{ccccccc} -\phi_{a}\rho x_{0,2} & 0 & \rho \phi_{a} & 0 & -\lambda_{1} & \left(1-\phi_{a}\right)\lambda_{a} & \phi_{a}\left(1-\phi_{a}\right)\lambda_{a}\\ -\rho \phi_{1}x_{0,2} & -x_{0,1}-x_{0,2} & \rho \phi_{1} & 1 & 0 & -\phi_{1}\lambda_{a} & \phi_{1}\lambda_{a}-2\phi_{1}\phi_{a}\lambda_{a}\\ -\phi_{1}\phi_{a}x_{0,2} & 0 & \phi_{1}\phi_{a} & 0 & 0 & 0 & 0\\ 0 & 0 & 0 & 0 & 1-\phi_{1} & 0 & 0\\ 0 & 0 & 0 & 0 & 0 & \phi_{1}\left(1-\phi_{a}\right) & \phi_{1}\phi_{a}\left(1-\phi_{a}\right) \end{array}\right], \end{array}$$

has rank 4, but there are five parameters so this model is parameter redundant. In the Maple code the estimable parameter combinations are shown to be $\phi_{a},\phi_{1}\rho ,\left(1-\phi_{1}\right)\lambda_{1}$, and $\phi_{1}\lambda_{a}$.

The same model with $\lambda_{1}=\lambda_{a}$ is also considered in the Maple code . The derivative matrix also has rank 4, but this time there are four parameters, so it is possible to estimate all the parameters in this model.□

### 3.2 Method B: reparametrization

Suppose there are two different data sets within the integrated model. The exhaustive summaries for these different data sets are $\kappa_{B,1}$ and $\kappa_{B,2}$, with parameters $\theta_{1}$ and $\theta_{2}$ of length *p*_1_ and *p*_2_, respectively. Method B involves using the following theorem.

Theorem 3.1 If the rank of $D_{1,1}\left(\theta_{1}\right)=\left[\partial \kappa_{B,1}\left(\theta_{1}\right)/\partial \theta_{1}\right]$ is $q_{1}<p_{1}$, then reparameterize $\kappa_{B,1}$ in terms of its estimable parameters combinations, which we denote $s_{B,1}$. If the rank of $D_{1,1}\left(\theta_{1}\right)$ is *p*_1_, then set $s_{B,1}=\theta$. Next rewrite $\kappa_{B,2}$ in terms of $s_{B,1}$. This gives $\kappa_{B,2}\left(\theta_{2}\right)=\kappa_{2}\left(s_{B,1},\theta_{2,ex}\right)$, where $\theta_{2,ex}$ is a vector of length $p_{2,ex}$ consisting of any additional parameters. Let the rank of $D_{2,2}=\left[\partial \kappa_{B,2}\left(s_{B,1},\theta_{2,ex}\right)/\partial \theta_{2,ex}\right]$ equal $r_{ex}$. Then the integrated model has rank $q_{1}+r_{ex}$.

The proof of Theorem 3.1 is given in the Supporting Information Appendix A.

The advantage of this method is that the model rank can be found by calculating the rank of two smaller and simpler derivative matrices, compared with the combined exhaustive summary method that requires calculating the rank of one larger more complex derivative matrix. This method is therefore useful for more complex models.

Remark 1 Suppose that $\partial \kappa_{B,1}/\partial \theta_{1}$ and $\partial \kappa_{B,2}/\partial \theta_{2}$ are individually full rank with ranks $q_{1}=p_{1}$ and *p*_2_ and the integrated model has $p_{2}-p_{2,ex}$ parameters common to both exhaustive summaries, so that there are $p_{1}+p_{2,ex}$ parameters in the integrated model. A natural consequence of Theorem 3.1 is the already obvious fact that the integrated model will also be full rank with rank $p_{1}+p_{2,ex}$.

Remark 2 Suppose that $\theta_{1}$ and $\theta_{2}$ have no parameters in common and the rank of $\partial \kappa_{B,2}/\partial \theta_{2}$ is *q*_2_, then the rank of the combined model is $q_{1}+q_{2}$.

Remark 3 If $\theta_{2,ex}$ consists of only one parameter, then D_2, 2_ will trivially have full rank 1. Therefore the integrated model will have rank $q_{1}+1$.

Remark 4 The estimable parameter combinations, $s_{B,1}$, can be replaced by any reparametrization with rank$\left(\partial s_{B,1}/\partial \theta_{1}\right)=q_{1}$ and Theorem 3.1 will still apply.

The proofs of Remarks 1–4 are given in Supporting Information Appendix A.

#### Example 1c continues

Consider the integrated model again but where the recovery probability is not age‐dependent, such that $\lambda_{1}=\lambda_{a}$. We start with the state‐space model and use the *z*‐transform exhaustive summary so that

$$\begin{array}{c} \kappa_{B,1}=\left[\begin{array}{c} -\rho \phi_{1}\phi_{a}x_{0,2}\\ -\phi_{a}\left(x_{0,1}+x_{0,2}\right)\\ \rho \phi_{1}\phi_{a}\\ \phi_{a} \end{array}\right]. \end{array}$$

The parameters for the first part are $\theta =[\phi_{1},\phi_{a},\rho ]$. The derivative matrix $\partial \kappa_{B,1}/\partial \theta_{1}$ has rank 2 and estimable parameter combinations $\phi_{a}$ and $\phi_{1}\rho$. The reparametrization is then the estimable parameter combinations with $s_{B,1}={\left[s_{1,1},s_{1,2}\right]}^{\prime }={\left[\phi_{a},\phi_{1}\rho \right]}^{\prime }$. The second part consists of the ring‐recovery model with $\lambda_{1}=\lambda_{a}$. The exhaustive summary $\kappa_{B,2}$ is rewritten in terms of $s_{B,1}$,

$$\begin{array}{c} \kappa_{B,2}=\left[\begin{array}{c} \left(1-\phi_{1}\right)\lambda_{1}\\ \phi_{1}\left(1-\phi_{a}\right)\lambda_{a}\\ \phi_{1}\phi_{a}\left(1-\phi_{a}\right)\lambda_{a} \end{array}\right]\;\text{becomes}\;\kappa_{B,2}\left(s_{B,1},\theta_{2,ex}\right)=\left[\begin{array}{c} \lambda (\rho -s_{1,2})/\rho \\ \lambda s_{1,2}\left(1-s_{1,1}\right)/\rho \\ \lambda s_{1,2}s_{1,1}\left(1-s_{1,1}\right)/\rho \end{array}\right], \end{array}$$

with $\theta_{2,ex}=\left[\lambda ,\rho \right]$. The derivative matrix,

$$\begin{array}{c} D_{2,2}=\frac{\partial \kappa_{2}\left(s_{B,1},\theta_{2,ex}\right)}{\partial \theta_{2,ex}}=\left[\begin{array}{ccc} \frac{\rho -s_{1,2}}{\rho } & \frac{s_{1,2}\left(1-s_{1,1}\right)}{\rho } & \frac{s_{1,1}s_{1,2}\left(1-s_{1,1}\right)}{\rho }\\ \frac{\lambda s_{1,2}}{\rho^{2}} & -\frac{\lambda s_{1,2}\left(1-s_{1,1}\right)}{\rho^{2}} & -\frac{\lambda s_{1,1}s_{1,2}\left(1-s_{1,1}\right)}{\rho^{2}} \end{array}\right], \end{array}$$

has rank 2. By Theorem 3.1 the rank of the integrated model is $q_{1}+r_{ex}=2+2=4$, which is an alternative method of showing this model is not parameter redundant. Method B for the model with $\lambda_{1}\neq \lambda_{a}$ is demonstrated in the Maple code.□

## 4 Further examples

This section provides further examples of applications of the methods for state‐space models. Example 4 is a more complex linear state‐space model. Example 5 combines a more complex state‐space model integrated with three other data sets.

Examples 6–8 are given in Supporting Information Appendix B and provide examples of integrated models not involving a state‐space model component. Examples 6 and 7 illustrate the use of Remarks 1 and 3, respectively. Example 8 illustrates how method B can be used for a more complicated model structure. Full details for each example are available in the Supporting Information Maple code.

### 4.1 Example 4

Multistate analyses are a natural generalization of single‐state ecological models (see Lebreton et al., 2009 and Chapter 5 of McCrea and Morgan, 2014). This is a multisite example based on models of McCrea et al. (2010). We suppose abundance data are collected at two different sites. Individuals are assumed to fall into one of three categories: newborn, immature, and breeder. The variables $x_{1}\left(t\right)$ and $x_{2}\left(t\right)$ denote the number of newborn animals in sites 1 and 2, respectively; $x_{3}\left(t\right)$ and $x_{4}\left(t\right)$ denote the number of immature animals in sites 1 and 2, respectively; $x_{5}\left(t\right)$ and $x_{6}\left(t\right)$ denote the number of breeding animals in sites 1 and 2, respectively. Unlike the age‐class states used in earlier examples, there is no specific age at which individuals recruit to a breeding state. Instead immature individuals move to a breeding state with probability $\pi_{k}$, for site *k*, in any given year. It is assumed that individuals will not move between the sites until they become breeding individuals, and the probability of moving from site *k* to *l* is $\psi_{k,l}$. The transition matrix is,

$$\begin{array}{c} A=\left[\begin{array}{cccccc} 0 & 0 & 0 & 0 & \rho_{1}\phi_{1,1} & 0\\ 0 & 0 & 0 & 0 & 0 & \rho_{1}\phi_{1,2}\\ \phi_{1,1} & 0 & \phi_{1,1}\left(1-\pi_{1}\right) & 0 & 0 & 0\\ 0 & \phi_{1,2} & 0 & \phi_{1,2}\left(1-\pi_{2}\right) & 0 & 0\\ 0 & 0 & \phi_{1,1}\pi_{1} & 0 & \phi_{2,1}\left(1-\psi_{1,2}\right) & \phi_{2,2}\psi_{2,1}\\ 0 & 0 & 0 & \phi_{1,2}\pi_{2} & \phi_{2,1}\psi_{1,2} & \phi_{2,2}\left(1-\psi_{2,1}\right) \end{array}\right], \end{array}$$

where $\phi_{1,k}$ is the survival probability of immature animals at site $k,\phi_{2,k}$ is the survival probability of breeders at site *k*, and $\rho_{k}$ is the fecundity of a breeding animal. Assuming that only breeders are observed at both sites, the observation matrix is $C=\left[\begin{array}{cccccc} 0 & 0 & 0 & 0 & 1 & 0\\ 0 & 0 & 0 & 0 & 0 & 1 \end{array}\right]$. The initial values of the state equation are assumed to be known values $x_{0}={\left[x_{0,1},x_{0,2},x_{0,3},x_{0,4},x_{0,5},x_{0,6}\right]}^{\prime }$.

There are n=6 states, so that using Theorem 2.2 (Option II) results in an exhaustive summary with 2n=12 terms. Due to the complexity of the later terms we use Option III instead and consider the first five exhaustive summary terms. However, even with five exhaustive summary terms Maple cannot calculate the rank directly. Instead we use the reparametrization

$$\begin{array}{c} s=\left[\begin{array}{c} s_{1}\\ s_{2}\\ s_{3}\\ s_{4}\\ s_{5}\\ s_{6}\\ s_{7}\\ s_{8}\\ s_{9}\\ s_{10} \end{array}\right]=\left[\begin{array}{c} \phi_{1,1}\pi_{1}x_{0,3}+\phi_{2,1}\left(1-\psi_{1,2}\right)x_{0,5}+\phi_{2,2}\psi_{2,1}x_{0,6}\\ \phi_{1,2}\phi_{2}x_{0,4}+\phi_{2,1}\psi_{1,2}x_{0,5}+\phi_{2,2}\left(1-\psi_{2,1}\right)x_{0,6}\\ \phi_{1,1}\pi_{1}\left\{\phi_{1,1}x_{0,1}+\phi_{1,1}\left(1-\phi_{1}\right)x_{0,3}\right\}\\ \phi_{1,2}\pi_{2}\left\{\phi_{1,2}x_{0,2}+\phi_{1,2}\left(1-\phi_{2}\right)x_{0,4}\right\}\\ \phi_{2,1}\left(1-\psi_{1,2}\right)\\ \phi_{2,2}\psi_{2,1}\\ \phi_{2,1}\psi_{1,2}\\ \phi_{2,2}\left(1-\psi_{2,1}\right)\\ \phi_{1,1}\pi_{1}\left[\rho_{1}\phi_{1,1}^{2}x_{0,5}+\phi_{1,1}\left(1-\pi_{1}\right)\left\{\phi_{1,1}x_{0,1}+\phi_{1,1}\left(1-\pi_{1}\right)x_{0,3}\right\}\right]\\ \phi_{1,2}\pi_{2}\left[\rho_{2}\phi_{1,2}^{2}x_{0,6}+\phi_{1,2}\left(1-\pi_{2}\right)\left\{\phi_{1,2}x_{0,2}+\phi_{1,2}\left(1-\pi_{2}\right)x_{0,4}\right\}\right] \end{array}\right]. \end{array}$$

The exhaustive summary written in terms of $s,\kappa \left(s\right)={\left[s_{1},s_{2},s_{1}s_{5}+s_{2}s_{6}+s_{3},s_{1}s_{7}s_{2}s_{8}+s_{4},...\right]}^{\prime }$, is differentiated with respect to s, to form the derivative matrix $D_{s}=\left[\partial \kappa \left(s\right)/\partial s\right]$, which has full rank 10. By the Reparametrization theorem the original parametrization also has rank 10. As this model has 10 parameters, this example is not parameter redundant. Although in practice it is possible to fit models to census data alone, we also note that there can be problems with boundary estimation (see Table 1 of McCrea et al., 2010).□

### 4.2 Example 5

Reynolds et al. (2009) combine four different data sets collected on a colony of common guillemots on the Isle of May, to create an integrated population model over *T* years. There is interest in whether survey effort could be reduced in collecting data, including stopping collection of one set of data (Lahoz‐Monfort et al., 2014). Here we discuss one aspect of reducing effort, demonstrating which parameters can be estimated with different combinations of the four data sets.

The first data set consists of productivity data, where the eggs laid by pairs of birds are monitored and the number of eggs that resulted in fledged chicks is recorded. A binomial model is fitted to these data so an exhaustive summary is $\kappa_{1}={\left[n_{1}\rho_{1},n_{2}\rho_{2},...,n_{T}\rho_{T}\right]}^{\prime }$, where $n_{t}$ is the number of eggs laid in year *t* and $\rho_{t}$ denotes the productivity rate in year *t*. The vector of parameters is $\theta_{1}=\rho =\left[\rho_{1},\rho_{2},...,\rho_{T}\right]$. It is obvious that all parameters in this model can be estimated.

The second data set is adult capture‐recapture data, where adult birds are marked and live recaptures are recorded. An exhaustive summary for the model fitted to this data set is $\kappa_{2}={\left[\phi_{1}p_{2},...,\phi_{T-1}p_{T},\phi_{1}\left(1-p_{2}\right),...,\phi_{T-2}\left(1-p_{T-1}\right)\right]}^{\prime }$ (Hubbard et al., 2014), where $\phi_{t}$ is the survival probability for year *t* and $p_{t}$ is the capture probability for year *t*. The vector of parameters is $\theta_{2}=\left[\Phi_{a},p\right],$ where $\Phi_{a}=\left[\phi_{a,1},...,\phi_{a,T-1}\right]$ and $p=[p_{a,2},...,p_{a,T}]$. This model is known to be parameter redundant, with estimable parameter combinations $\phi_{a,1},...,\phi_{a,T-2},p_{a,2},...,p_{a,T-1}$, and $\phi_{a,T-1}p_{a,T}$ (see, e.g., Cole et al., 2010).

The third data set consists of chick capture‐recapture‐recovery data. In this study birds are marked as young and then birds are either resighted alive or recovered dead at subsequent sampling occasions. An exhaustive summary for the capture‐recapture‐recovery model fitted to this data set is $\kappa_{3}$, which consists of the terms $\psi_{i,t}\tau_{i,t}\phi_{i-1,t}q_{i,t}$ for i=0,...,T−1 and t=i,...,T−1, $\psi_{i,j}\tau_{i,j}\phi_{i-1,j}(1-q_{i,j}$) for i=0,...,T−2, and j=i,...,T−2 and $\phi_{i-1,j}\left(1-\lambda_{j}\right)$ for i=0,...,T−1 and j=i,...,T−1 (Hubbard et al., 2014). The parameter $\lambda_{t}$ is the recovery probability for year *t*. The parameter $\psi_{i,j}=\psi$ if i=4 and 1 otherwise, where ψ is the probability that a bird does not permanently emigrate. The parameter $\tau_{i,j}=\tau$ if i>4 and 1 otherwise, where τ is the probability of tag loss. The resighting probability, $q_{i,j}$ is different from the recapture probability for the adult data set as the methods used are different. We let $q=[q_{2,2},...,q_{2,T-1},q_{3,3},...,q_{3,T-1},q_{4},q_{a}]$, where $q_{4,t}=q_{4}$ for all *t* and $q_{i,t}=q_{a}$ for all i>4 and all *t*. The vector of parameters is $\theta_{3}=\left[\Phi_{i},\Phi_{a}^{☆},q,\Lambda \right]$, where $\Phi_{a}^{☆}=\left[\phi_{a,5},...,\phi_{a,T-1}\right]$ and $\Phi_{i}=\left[\phi_{0,1},...,\phi_{0,T-1},\phi_{1},\phi_{2},\phi_{3}\right]$ with $\phi_{i,t}=\phi_{i}$ for i>0. We show in the Maple code that this model has full rank 5T−10.

The final data set consists of abundance data modeled by a state‐space model with the expected number of juveniles at time *t* equal to $E\left(J_{t}\right)=\rho_{t}\phi_{0,t}\phi_{1}\phi_{2}\phi_{3}E\left(x_{t}\right)/2$ and the expected number of adults at time *t* equal to $E\left(x_{t}\right)=\psi \phi_{a,t-1}J_{t-5}+\phi_{a,t-1}E\left(x_{t-1}\right)$. Only the adults are observed so the expectation of the observation process is $E\left(y_{t}\right)=E\left(x_{t}\right)$. For T=7, the exhaustive summary is $\kappa_{4}=\left[x_{0,5}\phi_{a,5}+\frac{1}{2}x_{0,1}\rho_{1}\psi \phi_{0,1}\phi_{1}\phi_{2}\phi_{3}\phi_{a,5},\left(x_{0,5}\phi_{a,5}+1\frac{1}{2}x_{0,1}\rho_{1}\phi_{0,1}\psi \phi_{1}\phi_{2}\phi_{3}\phi_{a,5}\right)\phi_{a,6}+\frac{1}{2}x_{0,2}\rho_{2}\psi \phi_{0,2}\phi_{1}\phi_{2}\phi_{3},\sigma_{N}^{2}\right]$. The rank of the derivative matrix for the state‐space model will always be limited by the number of exhaustive summary terms (see Cole et al., 2012 for details of this method); in this case the rank is 3. In general the rank of this model is then T−4 and only $\sigma_{N}$ can be estimated. We let $\rho^{☆}=\left[\rho_{1},...,\rho_{T-5}\right]$ denote the productivity parameters used in the state‐space model so the vector of parameters is $\theta_{4}=\left[\psi ,\rho^{☆},\Phi_{i}^{☆},\Phi_{a}^{☆},\sigma_{N}\right]$, where $\Phi_{i}^{☆}=\left[\phi_{0,1},...,\phi_{0,T-5},\phi_{1},\phi_{2},\phi_{3}\right]$.

It is assumed that we can estimate all the parameters, $\theta =[\Phi_{i},\Phi_{a},q,\Lambda ,\tau ,\psi ,p_{a}\rho ]$, when all the models are combined. We confirm that is the case below. We might also like to consider whether we actually needed all four data sets to estimate the parameters that are of biological interest. So in Table 2 we show the parameter redundancy results for different combinations of the four data sets, including each data set alone and all four data sets combined.

**Table 2:** Parameter redundancy results for Example 6

	Prod.	Adult CR	Chick CRR	State space
	r=T	r=2T−3	r=5T−5	r=T−4
	d=0	d=1	d=0	d=2T−6
	(ρ)	$(\phi_{a,1},...,\phi_{a,T-2},$	$(\Phi_{i},\Phi_{a}^{☆},q,\Lambda ,$	($\sigma_{N}$)^a^
		$p_{a,2},...,p_{a,T-1},$	τ,ψ)	
		$\phi_{a,T-1}p_{a,T}$)		
		r=3T−3	r=6T−5	r=2T−4
		d=1	d=0	d=T−1
Prod.		($\phi_{a,1},...,\phi_{a,T-2},$	($\rho ,\Phi_{i},\Phi_{a}^{☆},q,$	($\rho ,\sigma_{N}$)^a^
		$p_{a,2},...,p_{a,T-1},$	Λ,τ,ψ)	
		$\phi_{a,T-1}p_{a,T},\rho$)		
			r=6T−2,	r=3T−7
Adult CR			d=0	d=T
			$(\Phi_{i},\Phi_{a},q,\Lambda ,$	($\phi_{a,1},...,\phi_{a,T-2},\phi_{a,T-1}p_{a,T},$
			$\tau ,\psi ,p_{a}$)	$p_{a,2},...,p_{a,T-1},\sigma_{N}$)^a^
				r=6T−9
Chick CRR				d=0
				$(\Phi_{i},\Phi_{a}^{☆},q,\Lambda ,\tau ,\psi ,\rho^{☆},\sigma_{N}$)
Prod. and			r=7T−2	r=4T−7
adult CR			d=0	d=5
			$(\rho ,\Phi_{i},\Phi_{a},q,$	($\phi_{a,1},...,\phi_{a,T-2},\phi_{1}\phi_{2}\phi_{3}\psi \phi_{0,1}$
			$\Lambda ,\tau ,\psi ,p_{a}$)	$p_{a,2},...,p_{a,T-1},\phi_{a,T-1}p_{a,T},\rho ,$
				$\frac{\phi_{0,2}}{\phi_{0,1}},...,\frac{\phi_{0,T-6}}{\phi_{0,1}},\sigma_{N})$ a
Prod. and				r=6T−4
chick CRR				d=0
				$(\Phi_{i},\Phi_{a}^{☆},q,\Lambda ,\tau ,\psi ,\rho ,\sigma_{N}$)
Adult CR and				r=7T−6
chick CRR				d=0
				$(\Phi_{i},\Phi_{a},q,\Lambda ,\tau ,\psi ,p_{a},\rho^{☆},\sigma_{N}$)
Prod. and				r=7T−1
adult CR and				d=0
chick CRR				$(\Phi_{i},\Phi_{a},q,\Lambda ,\tau ,\psi ,p_{a},\rho ,\sigma_{N}$)

The model rank *r* and deficiency *d* are given for each combination of the four data sets with the estimable parameters in brackets. d>0 indicates a model is parameter redundant. d=0 indicates that it is theatrically possible to estimate all the parameters. “Prod.” denotes the productivity data set, “adult CR” denotes the adult capture‐recapture data set, “chick CRR” denotes the chick capture‐recapture‐recovery data set, and “state space” is the abundance data set.

a The other estimable parameter combination(s) are exhaustive summary terms.

To find the parameter redundancy results of Table 2 we use method B. Some of the combinations have no parameters in common, such as the productivity data combined with the adult capture‐recapture data, in which case Remark 2 is applied to obtain the results. If two models that are already full rank are combined, such as the productivity data and the chick capture‐recapture‐recovery data set, we apply Remark 1 to obtain that the combined model is also full rank. The other combinations use Theorem 3.1 directly with full details for each combination given in the Maple code of the Supporting Information.

For the adult capture‐recapture data and chick mark‐recapture‐recovery data combined, we start with the chick mark‐recapture‐recovery data exhaustive summary, $\kappa_{B,1}=\kappa_{3}$ and note that it is full rank $q_{1}=5T-10$ , so $s_{B,1}=\theta_{3}$. Then we examine the adult capture‐recapture data set, so that $\kappa_{B,2}=\kappa_{2}$. The extra parameters are $\theta_{2,ex}=\left[p,\phi_{a,1},...,\phi_{a,4}\right]$. For T=7, the derivative matrix $D_{2,2}=\left[\partial \kappa_{B,1}/\partial \theta_{2,ex}\right]$ is of full rank 10. As adding an extra year adds only one extra parameter, by a trivial application of the Extension theorem D_2, 2_ will always have full rank $r_{ex}=T+3$. By Theorem 3.1, the rank for this combined model is $q_{1}+r_{ex}=6T-6$.

When all four data sets are combined in one integrated model, this parametrization of resulting model is full rank, by Remark 1 as the productivity data set is full rank and the adult capture‐recapture data, chick mark‐recapture‐recovery data, and census data combined is full rank.

## 5 Discussion

Discrete state‐space models have become very popular for ecological applications and it is essential that users are conscious of parameter redundancy, and in particular whether or not a given parametrization of their model is parameter redundant. If a particular parametrization of a model is parameter redundant, it will have multiple maximum‐likelihood estimates. To obtain accurate estimates of the biological parameters of interest some form of constraints or reparametrization of the model is needed. Alternatively several data sets with different models could be combined to create an integrated population model.

State‐space models can also be fitted using Bayesian methodology and the issue of identifiability remains important. Nonidentifiability can result in poor mixing and slow convergence of the MCMC sample (see, e.g., page 187 of Carlin and Louis, 1996). Consider a model with a particular parametrization that is parameter redundant under the classical formation. If uninformative priors are used, then the posterior will be nonidentifiable in a Bayesian analysis. Using informative priors can result in an identifiable posterior, although this is not always the case. Nonidentifiability in a Bayesian analysis can be investigated by including prior information in the exhaustive summary. Alternatively Garrett and Zeger (2000) investigate weak identifiability by comparing marginal posteriors and priors. Weak identifiability is defined by Gelfand and Sahu (1999) as occurring when the data provide little information about a parameter, so that the prior and marginal posterior of a parameter are close under some suitable metric. To investigate weak identifiability, Garrett and Zeger (2000) display the marginal posterior and prior on the same plot and examine the percentage overlap. If the overlap is more than 35% a model is weakly identifiable. Gimenez et al. (2009) found this ad hoc guideline to be effective for capture‐recapture models.

We have shown how it is possible to detect parameter redundancy in state‐space models. In order to diagnose parameter redundancy, it is necessary to derive exhaustive summaries and here two approaches have been developed: the *z*‐transform exhaustive summary and the expansion exhaustive summary. The *z*‐transform approach has the advantage of giving a simpler exhaustive summary, however it is only applicable for linear discrete state‐space models without time‐dependent parameters. The alternative is to use the expansion exhaustive summary. This method is suitable for both linear and nonlinear models and models with time‐dependent parameters. The disadvantage of this method is that each successive term is more complicated than the previous. The reparametrization method or hybrid symbolic‐numeric method can be used if exhaustive summary terms become too complicated.

We have also shown how parameter redundancy can be investigated in integrated models. Method A is a straightforward extension of the standard derivative method. For more complex models, Maple may run out of memory calculating the rank of the derivative matrix, in which case method B can be used, as it only requires the calculation of ranks of simpler and smaller derivative matrices.

Mark‐recovery models, capture‐recapture models, and occupancy models are commonly used in ecology and can be written in terms of state‐space models (see, e.g., Gimenez et al., 2007; Royle, 2008; Chapter 11 of McCrea and Morgan, 2014). We show in Supporting Information Appendix C that the state‐space exhaustive summary for mark‐recovery models is the same as the exhaustive summary used in Cole et al. (2012), derived from the nonstate‐space derivation of the model. In Supporting Information Appendix C we also show that the state‐space exhaustive summaries for capture‐recapture models are not as simple as the exhaustive summaries provided in Hubbard et al. (2014). In general we recommend using the simplest exhaustive summary available. Hubbard et al. (2014) and Cole et al. (2012) also provide general results for a wide class of mark‐recovery models and capture‐recapture models, which can be used without using symbolic methods.

State‐space models are a special case of latent‐state models; other examples include hidden Markov models and hidden process models (see, e.g., Langrock et al., 2013). The extension of the methods of this paper to these more general model classes is an active piece of current research.

## Conflict of interest

*The authors have declared no conflict of interest*.

## Supporting information

As a service to our authors and readers, this journal provides supporting information supplied by the authors. Such materials are peer reviewed and may be re‐organized for online delivery, but are not copy‐edited or typeset. Technical support issues arising from supporting information (other than missing files) should be addressed to the authors.

Supporting Information

Code

## References

[bimj1702-bib-0050] Bellman, R. and Aström, K. J. (1970). On structural identifiability. Mathematical Biosciences 7, 329–339.

[bimj1702-bib-0001] Besbeas, P. , Freeman, S. N. , Morgan, B. J. T. and Catchpole, E. A. (2002). Integrating mark‐recapture‐recovery and census data to estimate animal abundance and demographic parameters. Biometrics 58, 540–547. 12229988 10.1111/j.0006-341x.2002.00540.x

[bimj1702-bib-0002] Buckland, S. T. , Newman, K. , Thomas, L. and Koesters, N. B. (2004). State‐space models for the dynamics of wild animal populations. Ecological Modelling 171, 157–175.

[bimj1702-bib-0003] Carlin, B. P. and Louis, T. A. (1996). Bayes and Empirical Bayes Methods for Data Analysis. Chapman and Hall/CRC, London, UK.

[bimj1702-bib-0004] Caswell, H. (2000). Matrix Population Models: Construction, Analysis and Interpretation (2nd edn.). Sinauer Associates Incorporated, U.S.

[bimj1702-bib-0005] Catchpole, E. A. and Morgan, B. J. T. (1997). Detecting parameter redundancy. Biometrika 84, 187–196.

[bimj1702-bib-0006] Catchpole, E. A. , Morgan, B. J. T. and Freeman, S. N. (1998). Estimation in parameter redundant models. Biometrika 85, 462–468.

[bimj1702-bib-0007] Catchpole, E. , A, Morgan B. J. T. , Freeman, S. N. and Peach, W. J. (1999). Modelling the survival of British lapwings Vanellus vanellus using weather covariates. Bird Study 46, 5–13.

[bimj1702-bib-0008] Catchpole, E. A. , Morgan, B. J. T. and Viallefont, A. (2002). Solving problems in parameter redundancy using computer algebra. Journal of Applied Statistics 29, 625–636.

[bimj1702-bib-0009] Chappell, M. J. and Gunn, R. N. (1998). A procedure for generating locally identifiable reparametrisations of unidentifiable non‐linear systems by the similarity transformation approach. Mathematical Biosciences 148, 21–41. 9597823 10.1016/s0025-5564(97)10004-9

[bimj1702-bib-0010] Chis, O. , Banga, J. R. and Balsa‐Canto, E. (2011). Structural identifiability of systems biology models: a critical comparison of methods. PLoS One 6, e27755. doi:10.1371/journal.pone.0027755 22132135 10.1371/journal.pone.0027755 PMC3222653

[bimj1702-bib-0011] Choquet, R. and Cole, D. J. (2012). A hybrid symbolic‐numerical method for determining model structure. Mathematical Biosciences 236, 117–125. 22366354 10.1016/j.mbs.2012.02.002

[bimj1702-bib-0012] Cole, D. J. (2012). Determining parameter redundancy of multi‐state mark‐recapture models for sea birds. Journal of Ornithology 152, S305–S315.

[bimj1702-bib-0013] Cole, D. J. and Morgan, B. J. T. (2010a). A note on determining parameter redundancy in age‐dependent tag return models for estimating fishing mortality, natural mortality and selectivity. Journal of Agricultural, Biological, and Environmental Statistics 15, 431–434.

[bimj1702-bib-0014] Cole, D. J. and Morgan, B. J. T. (2010b). Parameter redundancy with covariates. Biometrika 97, 1002–1005.

[bimj1702-bib-0015] Cole, D. J. , Morgan, B. J. T. , Catchpole, E. A. and Hubbard, B. A. (2012). Parameter redundancy of mark‐recovery models. Biometrical Journal 54, 507–523. 22688809 10.1002/bimj.201100210

[bimj1702-bib-0016] Cole, D. J. , Morgan, B. J. T. and Titterington, D. M. (2010). Determining the parametric structure of models. Mathematical Biosciences 228, 16–30. 20800072 10.1016/j.mbs.2010.08.004

[bimj1702-bib-0017] Dennis, B. , Ponciano, J. M. , Lele, S. R. , Taper, M. L. and Staples, D. F. (2006). Estimating density dependence, process noise, and observation error. Ecological Monographs 76, 323–341.

[bimj1702-bib-0018] Evans, N. D. and Chappell, M. J. (2000). Extensions to a procedure for generating locally identifiable reparametrisations of unidentifiable systems. Mathematical Biosciences 168, 137–159. 11121562 10.1016/s0025-5564(00)00047-x

[bimj1702-bib-0019] Forcina, A. (2008). Identifiability of extended latent class models with individual covariates. Computational Statistics and Data Analysis 52, 5263–5268.

[bimj1702-bib-0020] Freeman, S. N. and Morgan, B. J. T. (1990). Studies in the analysis of ring‐recovery data, Ring 13, 217–287.

[bimj1702-bib-0021] Freeman, S. N. and Morgan, B. J. T. (1992). A modelling strategy for recovery data from birds ringed as nestlings, Biometrics 48, 217–236. 1581486

[bimj1702-bib-0022] Garrett, E. S. and Zeger, S. L. (2000). Latent class model diagnosis. Biometrics 56, 1055–1066. 11129461 10.1111/j.0006-341x.2000.01055.x

[bimj1702-bib-0023] Gelfand, A. E. and Sahu, K. (1999). Identifiability, improper priors, and Gibbs sampling for generalized linear models. Journal of the American Statistical Association 94, 237–253.

[bimj1702-bib-0024] Gimenez, O. , Morgan, B. J. T. and Brooks, S. P. (2009). Weak identifiability in models for mark‐recapture‐recovery data In Thomson, D. , Cooch, E. G. and Conroy, M. J. (Eds.), Modeling Demographic Processes in Marked Populations. Springer Series, U.S.: Environmental and Ecological Statistics, 3, pp. 1057–1070.

[bimj1702-bib-0025] Gimenez, O. , Rossi, V. , Choquet, R. , Dehais, C. , Dorris, B. , Varella, H. , Vila, J. and Pradel, R. (2007). State‐space modelling of data on marked individuals. Ecological Modelling 206, 431–438.

[bimj1702-bib-0026] Gimenez, O. , Viallefont, A. , Catchpole, E. A. , Choquet, R. and Morgan, B. J. T. (2004). Methods for investigating parameter redundancy. Animal Biodiversity and Conservation 27, 1–12.

[bimj1702-bib-0027] Harvey, A. C. (1989). Forecasting, Structural Time Series Models and the Kalman Filter Cambridge University Press, Cambridge, UK.

[bimj1702-bib-0028] Hubbard, B. A. , Cole, D. J. and Morgan, B. J. T. (2014). Parameter redundancy in capture‐recapture‐recovery models. Statistical Methodology 17, 17–29.

[bimj1702-bib-0029] Hunter, C. M. and Caswell, H. (2009). Rank and redundancy of multistate mark‐recapture models for seabird populations with unobservable states In Thomson, D. , Cooch, E. G. and Conroy, M. J. (Eds.), Modeling Demographic Processes in Marked Populations. Springer Series, U.S.: Environmental and Ecological Statistics, 3, pp. 797–826.

[bimj1702-bib-0030] Jiang, H. , Pollack, K. H. , Brownie, C. , Hightower, J. E. , Hoenig, J. E. and Hearn, W. S. (2007). Age‐dependent tag return models for estimating fishing mortality, natural mortality and selectivity. Journal of Agricultural, Biological, and Environmental Statistics 12, 177–194.

[bimj1702-bib-0031] Lahoz‐Monfort, J. J. , Harris, M. P. , Morgan, B. J. M. , Freeman, S. N. and Wanless, S. (2014). Exploring the consequences of reducing survey effort for detecting individual and temporal variability in survival. Journal of Applied Ecology 51, 534–543.

[bimj1702-bib-0032] Langrock, R. , Borchers, D. L. and Skaug, H. J. (2013). Markov‐modulated nonhomogeneous Poisson processes for modeling detections in surveys of marine mammal abundance. Journal of the American Statistical Association 108, 840–851.

[bimj1702-bib-0033] Lebreton, J. , Morgan, B. J. T. , Pradel, R. , Freeman, S. N. (1995). A simultaneous survival rate analysis of dead recovery and live recapture data. Biometrics 51, 1418–1428.

[bimj1702-bib-0034] Lebreton, J. , Nichols, J. A. , Barker, R. J. , Pradel, R. and Spendelow, J. A. (2009). Modeling individual animal histories with multistate capture? Recapture models Caswell (Ed.), Advances in Ecological Research. Academic Press, pp. 87–173.

[bimj1702-bib-0035] Magaria, G. , Riccomagno, E. , Chappell, M. J. and Wynn, H. P. (2001). Differential algebra methods for the study of the structural identifiability of rational function state‐space models in biosciences. Mathematical Biosciences 174, 1–26. 11595254 10.1016/s0025-5564(01)00079-7

[bimj1702-bib-0036] McCrea, R. S. and Morgan, B. J. T. (2014). Analysis of Capture‐Recapture. Chapman and Hall, CRC Interdisciplinary Statistics Series, USA.

[bimj1702-bib-0037] McCrea, R. S. , Morgan, B. J. T. and Cole, D. J. (2013). Age‐dependent models for recovery data on animals marked at unknown age. Applied Statistics 62, 101–113.

[bimj1702-bib-0038] McCrea, R. S. , Morgan, B. J. T. , Gimenez, O. , Besbeas, P. , Lebreton, J. D. and Bregnballe, T. (2010). Multi‐site integrated population modelling. Journal of Agricultural, Biological, and Environmental Statistics 15, 539–561.

[bimj1702-bib-0039] Newman, K. B. , Buckland, S. T. , Lindley, S. T. , Thomas, L. and Fernandez, C. (2006). Hidden process models for animal population dynamics. Ecological Applications 16, 74–86. 16705962 10.1890/04-0592

[bimj1702-bib-0040] Pohjanpalo, H. (1978). System identifiability based on the power series of the solution. Mathematical Biosciences 41, 21–33.

[bimj1702-bib-0041] Pohjanpalo, H. (1982). Identifiability of deterministic differential models in state space. Technical Research Centre of Finland Research Report No. 56.

[bimj1702-bib-0042] Reynolds, T. J. , King, R. , Harwood, J. , Frederikesen, M. , Harris, M. P. and Wanless, S. (2009). Integrated data analyses in the presence of emigration and tag‐loss. Journal of Agricultural, Biological, and Environmental Statistics 14, 411–431.

[bimj1702-bib-0043] Robinson, R. A. (2010). Estimating age‐specific survival rates from historical data. Ibis 152, 651–653.

[bimj1702-bib-0044] Rothenberg, T. J. (1971). Identification in parametric models. Econometrica 39, 577–591.

[bimj1702-bib-0045] Royle, J. A. (2008). Modeling individual effects in the Cormack‐Jolly‐Seber model: a state‐space formulation. Biometrics 64, 364–370. 17725811 10.1111/j.1541-0420.2007.00891.x

[bimj1702-bib-0046] Schaub, M. and Abadi, F. (2011). Integrated population models: a novel analysis framework for deeper insights into population dynamics. Journal of Ornithology 152, 227–237.

[bimj1702-bib-0047] Thowsen, A. (1978). Identifiability of dynamic systems. International Journal of Systems Science 9, 813–825.

[bimj1702-bib-0048] Viallefont, A. , Lebreton, J. D. , Reboulet, A. M. and Gory, G. (1998). Parameter identifiability and model selection in capture‐recapture models : a numerical approach. Biometrical Journal 40, 1–13.

