# Supplementary material for: Parameter redundancy in discrete state‐space and integrated models
Source: Biom J. 2016 Jun 30;58(5):1071–90. doi: 10.1002/bimj.201400239 (PMC5031231; doi:10.1002/bimj.201400239)
Supplement: Supplementary file 1 — Supporting Information [file BIMJ-58-1071-s001.pdf]

Biometrical Journal ( ) , zzz–zzz / DOI: 10.1002/

Web based supplementary material for the paper parameter redundancy in discrete state-space and integrated models

Diana J. Cole[*,1] and **Rachel S. McCrea** [1]

[1] School of Mathematics, Statistics and Actuarial Science, University of Kent, Canterbury, Kent, CT2 7NF, England

Web Appendix A

This Appendix provides supporting information for Sections 2 and 3 of the main paper. This includes proofs of Theorems 2.1, Theorem 2.2 and Theorem 3.1 as well as a discussion on incorporating parameters in error terms.

Web Appendix A.1 Derivation of the Z-transform exhaustive summary (Theorem 2.1a)

As stated in the main paper, a continuous linear state-space model is defined by the equations,

$$\mathbf{y}(t,\theta) = \mathbf{C}(\theta)\mathbf{x}(t,\theta) \text{ and } \frac{\partial}{\partial t}\mathbf{x}(t,\theta) = \mathbf{A}(\theta)\mathbf{x}(t,\theta) + \mathbf{B}(\theta)\mathbf{u},$$

where $\mathbf{y}$ is the output function, $\mathbf{x}$ is the state-variable function, θ is a vector of unknown parameters, $\mathbf{u}$ is the input function and t is the time recorded on a continuous time scale. The matrices $\mathbf{A}$, $\mathbf{B}$, $\mathbf{C}$ are the compartmental, input and the output matrices respectively. The Laplace transform approach involves taking the Laplace transform of $\mathbf{y}(t,\theta)$ and rearranging the resulting equation results in the transfer function $\mathbf{Q}(s) = \mathbf{C}(s\mathbf{I} - \mathbf{A})^{-1}\mathbf{B}$. An exhaustive summary is then formed from the coefficients of s in the numerator and denominator of $\mathbf{Q}(s)$ (see, for example, Bellman and Aström, 1970 and Godfrey and Distefano, 1987).

As stated in equation 4 of the main paper, a discrete linear state-space model is defined by the equations

$$\mathbf{y}_t = \mathbf{A}_t\mathbf{x}_t + \eta_t \text{ and } \mathbf{x}_t = \mathbf{C}_t\mathbf{x}_{t-1} + \varepsilon_{t-1},\ t = 1,2,3,\ldots$$

where $\mathbf{A}_t$ is an $n \times n$ transition matrix, $\mathbf{C}_t$ is an $m \times n$ measurement matrix, $\mathbf{x}_0$ is a vector of initial values and η and ε are error processes with zero means. The model has n states with m states or combination of states observed, where $m \leq n$. If $\mathbf{A}_t = \mathbf{A}$ and $\mathbf{C}_t = \mathbf{C}$, so that neither matrix contains time-dependent parameters, then the model becomes,

$$\mathbf{y}_t = \mathbf{C}\mathbf{x}_t + \eta_t \text{ and } \mathbf{x}_t = \mathbf{A}\mathbf{x}_{t-1} + \varepsilon_{t-1},\ t = 1,2,3,\ldots\ ,$$

and the expectation of $\mathbf{y}_t$ is,

$$E(\mathbf{y}_t) = \mathbf{C}E(\mathbf{x}_t) \text{ with } E(\mathbf{x}_t) = \mathbf{A}E(\mathbf{x}_{t-1}),\ t = 1,2,3,\ldots$$

This can be rewritten as

$$E(\mathbf{y}_t) = \mathbf{C}E(\mathbf{x}_t) \text{ with } E(\mathbf{x}_t) = \mathbf{A}E(\mathbf{x}_{t-1}) + \mathbf{B}\delta_{t-1}, \tag{1}$$

*Corresponding author: e-mail:d.j.cole@kent.ac.uk, Phone: +44-1227-823664

where δ_t is equal to 1 at time $t = 0$ and 0 otherwise. The matrix $\mathbf{B} = \mathbf{A}\mathbf{v}$, with $\mathbf{v} = [x_{0,1}, x_{0,2}, \ldots, x_{0,n}]^T$. This reparameterises the model so that $\mathbf{B}\delta_{t-1}$ specifies the initial values rather than $\mathbf{x}_0$, which becomes $\mathbf{x}_0 = \mathbf{0}$.

The discrete analogy of Laplace transforms is Z-transforms. Let $\tilde{\mathbf{w}}_z$ denote the unilateral Z-transform of $E(\mathbf{w}_t)$, with $\tilde{\mathbf{w}}_z = \sum_{t=0}^{\infty} E(\mathbf{w}_t) z^{-t}$. As the Z-transform of $E(\mathbf{w}_{t-1})$ is $z^{-1}\tilde{\mathbf{w}}_z$, and the Z-transform of δ_{t-1} is z^{-1}, taking Z-transforms of (1) results in,

$$\tilde{\mathbf{x}}_z = z^{-1}\mathbf{A}\tilde{\mathbf{x}}_z + z^{-1}\mathbf{B} \Rightarrow \tilde{\mathbf{x}}_z = (z\mathbf{I} - \mathbf{A})^{-1}\mathbf{B} \Rightarrow \tilde{\mathbf{y}}_z = \mathbf{C}(z\mathbf{I} - \mathbf{A})^{-1}\mathbf{B}.$$

The transfer function is then defined as $\mathbf{Q}(z) = \mathbf{C}(z\mathbf{I} - \mathbf{A})^{-1}\mathbf{B} = \mathbf{C}(z\mathbf{I} - \mathbf{A})^{-1}\mathbf{A}\mathbf{v}$, which is of a similar form to the transfer function for continuous state-space models. The numerator and denominator of $\mathbf{Q}(z)$ are both polynomials in z. In the same way as for continuous state-space models we form the exhaustive summary from the non-constant coefficients of the powers of z is the numerator and denominator of $\mathbf{Q}(z)$. We do not include the constant terms as they will not affect the parameter redundancy results.

Web Appendix A.2 Derivation of the expansion exhaustive summary (Theorem 2.1b)

The Taylor series approach, used in continuous state-space models, involves expanding $\mathbf{y}$ using the differential of $\mathbf{y}$ evaluated at $t = 0$ (Pohjanpalo, 1978). We firstly consider discrete linear state-space models. We return again to the general form of the discrete linear state-space model, given by equation 4 of the main paper with

$$\mathbf{y}_t = \mathbf{A}_t\mathbf{x}_t + \eta_t \text{ and } \mathbf{x}_t = \mathbf{C}_t\mathbf{x}_{t-1} + \varepsilon_{t-1},\ t = 1, 2, 3, \ldots$$

In this discrete state-space model, $E(\mathbf{y})$ can be expanded instead by finding algebraic expressions for each successive $E(\mathbf{y}_t)$ starting at $t = 0$. We assume that the start value is $\mathbf{x}_0$, which can be either a vector of known initial values, or a vector consisting of some function of the parameters. We then proceed to expand each of the observation vectors in turn. For linear discrete state-space models this gives,

$$\begin{array}{rclrcl} E(\mathbf{x}_1) & = & \mathbf{A}_1\mathbf{x}_0 & E(\mathbf{y}_1) & = & \mathbf{C}_1 E(\mathbf{x}_1) = \mathbf{C}_1\mathbf{A}_1\mathbf{x}_0 \\ E(\mathbf{x}_2) & = & \mathbf{A}_2 E(\mathbf{x}_1) = \mathbf{A}_2\mathbf{A}_1\mathbf{x}_0 & E(\mathbf{y}_2) & = & \mathbf{C}_2 E(\mathbf{x}_2) = \mathbf{C}_2\mathbf{A}_2\mathbf{A}_1\mathbf{x}_0 \\ \vdots & & & \vdots & & \end{array}$$

The $E(\mathbf{y}_t)$ then form the expansion exhaustive summary with terms,

$$\kappa = \begin{bmatrix} \mathrm{E}(\mathbf{y}_1) \\ \mathrm{E}(\mathbf{y}_2) \\ \vdots \end{bmatrix} = \begin{bmatrix} \mathbf{C}_1\mathbf{A}_1\mathbf{x}_0 \\ \mathbf{C}_2\mathbf{A}_2\mathbf{A}_1\mathbf{x}_0 \\ \vdots \end{bmatrix}.$$

Note this assumes that there are no parameters in the error terms.

For non-linear discrete state-space models we expand directly giving

$$\begin{array}{rclrcl} \mathbf{x}_1 & = & g(\mathbf{x}_0) + \varepsilon_0 & \mathbf{y}_1 & = & h(\mathbf{x}_1) = h\{g(\mathbf{x}_0) + \varepsilon_0\} + \eta_1 \\ \mathbf{x}_2 & = & g(\mathbf{x}_1) + \varepsilon_1 = g\{g(\mathbf{x}_0) + \varepsilon_0\} + \varepsilon_1 & \mathbf{y}_2 & = & h(\mathbf{x}_2) = h[g\{g(\mathbf{x}_0) + \varepsilon_0\} + \varepsilon_1] + \eta_1 \\ \vdots & & & \vdots & & \end{array}$$

Again assuming there are no parameters in the error terms then the error terms can be considered as constant values that will have no effect on parameter redundancy results. Adding a constant is a one-to-one transformation. Therefore this can be simplified to the expansion exhaustive summary,

$$\kappa = \begin{bmatrix} h\{g(\mathbf{x}_0)\} \\ h[g\{g(\mathbf{x}_0)\}] \\ \vdots \end{bmatrix}.$$

We note that the linear expansion exhaustive summary could also be derived using this argument.

© WILEY-VCH Verlag GmbH & Co. KGaA, Weinheim

Web Appendix A.3 Proof of Theorem 2.2

The proof of Theorem 2.2 is similar to Thowsen (1978)'s proof for the same result in the Taylor series approach for continuous state-space models. Theorem 2.2 states that if $\mathbf{A}$ and $\mathbf{C}$ are constant and there are n states, then a simpler exhaustive summary consists of the terms $\kappa = [E(\mathbf{y}_1)', \ldots, E(\mathbf{y}_{2n})']'$.

The exhaustive summary can be split into three parts:

$$\left.\begin{array}{rcl} \mathbf{y}_1 &=& \mathbf{CAx}_0 \\ \mathbf{y}_2 &=& \mathbf{CA}^2\mathbf{x}_0 \\ \vdots && \\ \mathbf{y}_{n-1} &=& \mathbf{CA}^{n-1}\mathbf{x}_0 \end{array}\right\} \text{Part a}$$

$$\left.\begin{array}{rcl} \mathbf{y}_n &=& \mathbf{CA}^n\mathbf{x}_0 \\ \mathbf{y}_{n+1} &=& \mathbf{CA}^{n+1}\mathbf{x}_0 \\ \vdots && \\ \mathbf{y}_{2n} &=& \mathbf{CA}^{2n}\mathbf{x}_0 \end{array}\right\} \text{Part b}$$

$$\left.\begin{array}{rcl} \mathbf{y}_{2n+1} &=& \mathbf{CA}^{2n+1}\mathbf{x}_0 \\ \mathbf{y}_{2n+2} &=& \mathbf{CA}^{2n+2}\mathbf{x}_0 \\ \vdots && \end{array}\right\} \text{Part c}$$

As $\mathbf{A}$ is an $n \times n$ matrix, by the Caley-Hamilton Theorem,

$$\mathbf{A}^n = \delta_0\mathbf{I} + \delta_1\mathbf{A} + \delta_2\mathbf{A}^2 + \ldots + \delta_{n-1}\mathbf{A}^{n-1},$$

so that

$$\begin{array}{rcl} \mathbf{y}_n &=& \mathbf{C}(\delta_0\mathbf{I} + \delta_1\mathbf{A} + \delta_2\mathbf{A}^2 + \ldots + \delta_{n-1}\mathbf{A}^{n-1})\mathbf{x}_0 \\ &=& \delta_0\mathbf{Cx}_0 + \delta_1\mathbf{y}_1 + \delta_2\mathbf{y}_2 + \ldots + \delta_{n-1}\mathbf{y}_{n-1}. \end{array}$$

Similarly,

$$\begin{array}{rcl} \mathbf{y}_{n+1} &=& \mathbf{CAA}^n\mathbf{c}_0 \\ &=& \mathbf{CA}(\delta_0\mathbf{I} + \delta_1\mathbf{A} + \delta_2\mathbf{A}^2 + \ldots + \delta_{n-1}\mathbf{A}^{n-1})\mathbf{x}_0 \\ &=& \delta_0\mathbf{y}_1 + \delta_1\mathbf{y}_2 + \delta_2\mathbf{y}_3 + \ldots + \delta_{n-1}\mathbf{y}_n. \\ &=& \delta_0\mathbf{y}_1 + \delta_1\mathbf{y}_2 + \delta_2\mathbf{y}_3 + \ldots + \delta_{n-1}(\delta_0\mathbf{Cx}_0 + \delta_1\mathbf{y}_1 + \delta_2\mathbf{y}_2 + \ldots + \delta_{n-1}\mathbf{y}_{n-1}). \end{array}$$

It follows that all the exhaustive summary terms of parts b and c can be written in terms of $\mathbf{y}_1$ to $\mathbf{y}_{n-1}$, δ_0 to δ_{n-1} and $\mathbf{y}_0 = \mathbf{Cx}_0$. From part a we can uniquely determine $\mathbf{y}_1$ to $\mathbf{y}_{n-1}$. Therefore it follows that from part b we can uniquely determine δ_0 to δ_{n-1} and $\mathbf{y}_0$, and part c contains no additional information on the parameters and does not need to be considered. Therefore only the first $2n$ terms of the exhaustive summary are needed.

Web Appendix A.4 Error Terms

The exhaustive summaries of Theorem 2.1 in the main paper assume that there are no parameters in the error terms η and ε. Here we consider how the error terms are included in the exhaustive summaries.

One possible exhaustive summary for linear discrete state-space models can be derived by expanding the variance as well as the expectation. This results in the exhaustive summary

$$\kappa = \begin{bmatrix} \mathrm{E}(\mathbf{y}_1) \\ \mathrm{Var}(\mathbf{y}_1) \\ \mathrm{E}(\mathbf{y}_2) \\ \mathrm{Var}(\mathbf{y}_2) \\ \vdots \end{bmatrix}.$$

© WILEY-VCH Verlag GmbH & Co. KGaA, Weinheim

Example 1b continued

In Besbeas et al. (2002) the variance is,

$$\text{Var}(y_t) = \begin{bmatrix} 0 & 1 \end{bmatrix} \text{Var}(\mathbf{x}_t) + \sigma^2 \text{ with } \text{Var}(\mathbf{x}_t) = \begin{bmatrix} 0 & \rho\phi_1 \\ \phi_a(1-\phi_a) & \phi_a(1-\phi_a) \end{bmatrix} \text{E}(\mathbf{x}_{t-1}).$$

For $T = 2$ years the exhaustive summary consists of the expectation terms used previously of $\kappa_1 = [x_{0,1}\phi_a + x_{0,2}\phi_a,\ x_{0,2}\phi_1\phi_a\rho + x_{0,1}\phi_a^2 + x_{0,2}\phi_a^2, x_{0,1}\phi_1\phi_a^2\rho + 2x_{0,2}\phi_1\phi_a^2\rho + x_{0,1}\phi_a^3 + x_{0,2}\phi_a^3]'$ and the the variance terms

$$\kappa_2 = \begin{bmatrix} \text{Var}(y_1) \\ \text{Var}(y_2) \end{bmatrix} = \begin{bmatrix} (x_{0,1} + x_{0,2})\phi_a(1-\phi_a) + \sigma^2 \\ \phi_a(1-\phi_a)\{x_{0,2}\phi_1\rho + (x_{0,1}+x_{0,2})\phi_a^2(1-\phi_a)\} + \sigma^2 \end{bmatrix}.$$

The derivative matrix formed by differentiating $[\kappa_1', \kappa_2']'$ with respect to the parameters $\theta = [\phi_1, \phi_a, \rho, \sigma]$ has rank 3, as previously this result can be extended to any $T \geq 2$. This parameter redundant model can be shown to have the estimable parameter combinations $\phi_1\rho$, ϕ_a and σ. □

An alternative exhaustive summary, that is also applicable for non-linear discrete state-space, starts with the exhaustive summary,

$$\begin{bmatrix} h\{g(\mathbf{x}_0) + \varepsilon_0\} + \eta_1 \\ h[g\{g(\mathbf{x}_0) + \varepsilon_0)\} + \varepsilon_1] + \eta_1 \\ \vdots \end{bmatrix}.$$

The terms ε_i and η_i can be replaced replaced with appropriate functions such as the variance of these terms.

Consider the case where the variance of ε_i is a function of the estimable parameters of the model with known variances. The exhaustive summary could then be simplified to

$$\begin{bmatrix} h\{g(\mathbf{x}_0)\} + \text{Var}(\eta_1) \\ h[g\{g(\mathbf{x}_0)\}] + \text{Var}(\eta_1) \\ \vdots \end{bmatrix}.$$

Next suppose that there is a single variance parameter for the error term η_i so that $\text{Var}(\eta_i) = \sigma^2$. The exhaustive summary is then

$$\begin{bmatrix} h\{g(\mathbf{x}_0)\} + \sigma^2 \\ h[g\{g(\mathbf{x}_0)\}] + \sigma^2 \\ \vdots \end{bmatrix}.$$

Suppose with exhaustive summary terms,

$$\kappa_w(\theta) = \begin{bmatrix} h\{g(\mathbf{x}_0)\} \\ h[g\{g(\mathbf{x}_0)\}] \\ \vdots \\ h\{g^n(\mathbf{x}_0)\} \end{bmatrix}$$

the model has rank q with estimable parameter combinations β. Also assume the next term can be reparameterised in terms of β, so that $\kappa_{ex}(\beta, \sigma) = h\{g^{n+1}(\mathbf{x}_0)\} + \sigma^2$. We are interested in examining the parameter redundancy of the model with exhaustive summary,

$$\begin{bmatrix} \kappa(\theta,\sigma) \\ \kappa_{ex}(\theta,\sigma) \end{bmatrix} = \begin{bmatrix} h\{g(\mathbf{x}_0) + \sigma^2\} \\ h[g\{g(\mathbf{x}_0)\} + \sigma^2] \\ \vdots \\ h[g^n(\mathbf{x}_0) + \sigma^2] \\ h[g^{n+1}(\mathbf{x}_0) + \sigma^2] \end{bmatrix},$$

© WILEY-VCH Verlag GmbH & Co. KGaA, Weinheim

which we reparameterise as

$$\left[\begin{array}{c}\kappa(\beta,\sigma)\\ \kappa_{ex}(\beta,\sigma)\end{array}\right].$$

The appropriate derivative matrix can be partitioned as

$$\mathbf{D} = \left[\begin{array}{cc}\mathbf{D}_{1,1} & \mathbf{D}_{1,2}\\ \mathbf{D}_{2,1} & \mathbf{D}_{2,2}\end{array}\right] = \left[\begin{array}{cc}\frac{\partial\kappa}{\partial\beta} & \frac{\partial\kappa_{ex}}{\partial\beta}\\ \frac{\partial\kappa}{\partial\sigma} & \frac{\partial\kappa_{ex}}{\partial\sigma}\end{array}\right] \qquad (2)$$

Theorem 4 of Meyer (1973) states that if a matrix is partitioned as equation (2) then

$$\text{rank}(\mathbf{D}) = \text{rank}(\mathbf{D}_{1,1}) + \text{rank}(\mathbf{Y}) + \text{rank}(\mathbf{W}) + \text{rank}(\mathbf{U})$$

where $\mathbf{X}^-$ is the generalised inverse of $\mathbf{X}$, and $\mathbf{I}$ is the identity matrix and where $\mathbf{Y} = (\mathbf{I}-\mathbf{D}_{1,1}\mathbf{D}_{1,1}^-)\mathbf{D}_{1,2}$, $\mathbf{W} = \mathbf{D}_{2,1}(\mathbf{I}-\mathbf{D}_{1,1}^-\mathbf{D}_{1,1})$, and $\mathbf{U} = (\mathbf{I}-\mathbf{W}\mathbf{W}^-)(\mathbf{D}_{2,2}-\mathbf{D}_{2,1}\mathbf{D}_{1,1}^-\mathbf{D}_{1,2})(\mathbf{I}-\mathbf{Y}^-\mathbf{W})$.

Due to the structure of $\kappa(\theta,\sigma)$, $\mathbf{D}_{1,1} = \partial\kappa(\beta,\sigma)/\partial\beta$ is identical to $\partial\kappa_w(\beta)/\partial\partial\beta$, so that the rank of $\mathbf{D}_{1,1}$ is q. Note that $\mathbf{D}_{1,1}$ is a $q\times n$ matrix. Theorem 6.2.16 of Graybill (1969) states that if $\mathbf{D}_{1,1}$ is a $q\times n$ of rank q then $\mathbf{D}_{1,1}\mathbf{D}_{1,1}^- = \mathbf{I}$. Therefore $\mathbf{Y} = (\mathbf{I}-\mathbf{I})\mathbf{D}_{1,2} = \mathbf{0}$, which will have rank 0. In this case $\mathbf{W}$ is a $1\times n$ matrix with non-zero entries, so it will have rank 1. Using Theorem 6.2.16 of Graybill (1969) again $\mathbf{I}-\mathbf{W}\mathbf{W}^- = \mathbf{I}-\mathbf{I} = \mathbf{0}$, therefore $\mathbf{U} = \mathbf{0}$ with rank 0. We then get the result,

$$\text{rank}(\mathbf{D}) = q+1.$$

The estimable parameter combination will be β and σ.

Example 1b continued

Consider the state-space model with unknown variances, described above. From the main paper we know that the rank of model with known variances is 2 and the estimable parameter combinations are ϕ_a and $\phi_1\rho$. As the variance of ε_i can be written in terms of ϕ_a and $\phi_1\rho$ and $\text{Var}(\eta_i) = \sigma^2$, without further calculation we can deduce that the rank of the model with unknown variances is 3. This model is therefore parameter redundant with estimable parameters ϕ_a, σ and $\phi_1\rho$.

Web Appendix A.5 Proof of Theorem 3.1

The proof of Theorem 3.1 is based on the same idea as the extension theorem (Catchpole and Morgan 1997, Cole et al. 2010).

Suppose the two exhaustive summaries for an integrated model are $\kappa_{B,1}$ and $\kappa_{B,2}$ of length k_1 and k_2, with parameters θ_1 and θ_2 of length p_1 and p_2 respectively. The matrix $\mathbf{D}_{1,1}(\theta_1) = [\partial\kappa_{B,1}(\theta_1)/\partial\theta_1]$ has rank $q_1 \le p_1$. This first part of the integrated model can be reparameterised in terms of its estimable parameters or another reparameterisation, $\mathbf{s}_1$, of length q_1 with $\text{rank}(\partial\mathbf{s}_1/\partial\theta_1) = q_1$. If $q_1 = p_1$ we can use the original parameters, θ_1, as the reparameterisation. By Theorem 8 of Cole et al. (2010), $\mathbf{D}_{1,1}(\mathbf{s}_1) = [\partial\kappa_{B,1}(\mathbf{s}_1)/\partial\mathbf{s}_1]$ has full rank q_1. The exhaustive summary $\kappa_2(\theta_2)$ is then rewritten in terms of $\mathbf{s}_1$ as $\kappa_{B,2}(\theta_2) = \kappa_2(\mathbf{s}_1,\theta_2')$, where θ_2' is a vector of length p_2' consisting of the rest of the parameters of $\kappa_{B,2}$. The rank of $\mathbf{D}_{2,2} = [\partial\kappa_{B,2}(\mathbf{s}_1,\theta_2')/\partial\theta_2']$ is r'.

The derivative matrix for the integrated model is,

$$\mathbf{D}(\theta) = \left[\begin{array}{cc}\mathbf{D}_{1,1}(\theta_1) & \mathbf{D}_{1,2}(\theta)\\ \mathbf{0} & \mathbf{D}_{2,2}(\theta)\end{array}\right] = \left[\begin{array}{cc}\frac{\partial\kappa_{B,1}(\theta_1)}{\partial\theta_1} & \frac{\partial\kappa_{B,2}(\theta)}{\partial\theta_1}\\ \mathbf{0} & \frac{\partial\kappa_{B,2}(\theta)}{\partial\theta_{2,ex}}\end{array}\right],$$

© WILEY-VCH Verlag GmbH & Co. KGaA, Weinheim

where $\theta_{2,ex}$ is a vector consisting of the parameters of θ_2 that are not in θ_2. By Theorem 8 of Cole et al. (2010), $\text{rank}\{\mathbf{D}(\theta)\} = \text{rank}\{\mathbf{D}(\mathbf{s})\}$, where

$$\mathbf{D}(\mathbf{s}) = \begin{bmatrix} \mathbf{D}_{1,1}(\mathbf{s}_1) & \mathbf{D}_{1,2}(\mathbf{s}) \\ \mathbf{0} & \mathbf{D}_{2,2}(\mathbf{s}) \end{bmatrix} = \begin{bmatrix} \dfrac{\partial \kappa_{B,1}(\mathbf{s}_1)}{\partial \mathbf{s}_1} & \dfrac{\partial \kappa_{B,2}(\mathbf{s}_1, \theta_2')}{\partial \mathbf{s}_1} \\ \mathbf{0} & \dfrac{\partial \kappa_{B,2}(\mathbf{s}_1, \theta_2')}{\partial \theta_2'} \end{bmatrix}. \quad (3)$$

Theorem 4.2 of Meyer (1973) states that if a block-triangular matrix is partitioned as equation (3) then,

$$\begin{aligned} \text{rank}\{\mathbf{D}(\mathbf{s})\} &= \text{rank}\{\mathbf{D}_{1,1}(\mathbf{s}_1)\} + \text{rank}\{\mathbf{D}_{2,2}(\mathbf{s})\} + \\ &\quad \text{rank}[\{\mathbf{I} - \mathbf{D}_{1,1}(\mathbf{s}_1)\mathbf{D}_{1,1}^{-}(\mathbf{s}_1)\}\mathbf{D}_{1,2}(\mathbf{s})\{\mathbf{I} - \mathbf{D}_{2,2}^{-}(\mathbf{s})\mathbf{D}_{2,2}(\mathbf{s})\}], \end{aligned} \quad (4)$$

where $\mathbf{X}^{-}$ is the generalised inverse of $\mathbf{X}$ and $\mathbf{I}$ is the identity matrix.

Here $\mathbf{D}_{1,1}(\mathbf{s}_1)$ is a $q_1 \times k_1$ matrix which has rank q_1. Using Theorem 6.2.16 of Graybill (1969) again $\mathbf{D}_{1,1}(\mathbf{s}_1)\mathbf{D}_{1,1}^{-}(\mathbf{s}_1) = \mathbf{I}$. Therefore $\mathbf{I} - \mathbf{D}_{1,1}(\mathbf{s}_1)\mathbf{D}_{1,1}^{-}(\mathbf{s}_1) = \mathbf{0}$ so that $\text{rank}[\{\mathbf{I} - \mathbf{D}_{1,1}(\mathbf{s}_1)\mathbf{D}_{1,1}^{-}(\mathbf{s}_1)\}\mathbf{D}_{1,2}(\mathbf{s})\{\mathbf{I} - \mathbf{D}_{2,2}^{-}(\mathbf{s})\mathbf{D}_{2,2}(\mathbf{s})\}] = 0$. Thus $\text{rank}\{\mathbf{D}(\theta)\} = \text{rank}\{\mathbf{D}(\mathbf{s})\} = \text{rank}\{\mathbf{D}_{1,1}(\mathbf{s}_1) + \text{rank}(\mathbf{D}_{2,2}(\mathbf{s})\} = q_1 + r'$.

Remarks 1 to 4 are a direct result of the proof of Theorem 3.1.

Remark 1 states that if $\partial \kappa_{B,1}/\partial \theta_1$ and $\partial \kappa_{B,2}/\partial \theta_2$ are individually full rank with ranks $q_1 = p_1$ and p_2 and the integrated model has $p_2 - p_{2,ex}$ parameters common to both exhaustive summaries, there are $p_1 + p_{2,ex}$ parameters in the integrated model. The integrated model will also be full rank with rank $p_1 + p_{2,ex}$. This is true as $\theta_1 = \mathbf{s}_{B,1}$ and $\theta_{2,ex}$ consists of the $p_{2,ex}$ parameters not in θ_1. Because $[\partial \kappa_{B,2}(\theta_2)/\partial \theta_2]$ is full rank, $\mathbf{D}_{2,2} = [\partial \kappa_{B,2}(\mathbf{s}_{B,1}, \theta_{2,ex})/\partial \theta_{2,ex}]$ will also be full rank.

Remark 2 states that if θ_1 and θ_2 have no parameters in common and the rank of $\partial \kappa_{B,2}/\partial \theta_2$ is q_2 then the rank of the combined model is $q_1 + q_2$. We can apply equation (4) to $\mathbf{D}(\theta)$ and note that if θ_1 and θ_2 have no parameters in common, $\mathbf{D}_{1,2}(\theta) = \mathbf{0}$. Therefore $\text{rank}[\{\mathbf{I} - \mathbf{D}_{1,1}(\theta_1)\mathbf{D}_{1,1}^{-}(\theta_1)\}\mathbf{D}_{1,2}(\theta)\{\mathbf{I} - \mathbf{D}_{2,2}^{-}(\theta)\mathbf{D}_{2,2}(\theta)\}] = 0$. So that $\text{rank}\{\mathbf{D}(\theta)\} = \text{rank}\{\mathbf{D}_{1,1}(\theta_1)\} + \text{rank}\{\mathbf{D}_{2,2}(\theta_2)\} = q_1 + q_2$.

Remark 3 states that if $\theta_{2,ex}$ consists of only one parameter, then $\mathbf{D}_{2,2}$ will trivially have full rank 1. Therefore the integrated model will have rank $q_1 + 1$. This result stems from the fact that if $\theta_{2,ex}$ consists of only one parameter, $\mathbf{D}_{2,2}$ will be a $n \times 1$ matrix with at least 1 non-zero entry. Such a matrix will always have rank 1.

The proof of remark 4 is included in the proof of Theorem 3.1 above.

Web Appendix B

This section provides further examples of integrated models that do not include state-space models.

Example 6: Combining independent capture-recapture and mark-recovery data sets

The paper Lebreton et al. (1995) examines two independent data sets. The first is capture-recapture data which involves the live recapture of birds. The second is mark-recovery data which involves the recovery of dead marked birds, rather than the live recapture of marked birds; the mark-recovery model is described in more detail in Example 3. In Lebreton et al. (1995) the model for the mark-recovery data has three survival parameters, ϕ_1 and ϕ_2 for first and second year survival and ϕ_a for adult survival, as well as one recovery probability, λ. The model for the recovery data has one survival parameter in common with the mark-recovery data, ϕ_a, as well as having a recapture probability that is dependent on time, p_t. Separately the models for the mark-recovery data and recapture data are both full rank. It is not stated in Lebreton et al. (1995), but it is assumed to be obvious that this combined model is also full rank. This can now be formally stated to be true using Remark 1 of the main paper.

© WILEY-VCH Verlag GmbH & Co. KGaA, Weinheim

Example 7: Integrated mark-recovery model

For some mark-recovery data sets, such as mallard (*Anas platyrhynchos*) mark-recovery data, there is data on birds marked as both juveniles and adults (see, for example, Freeman et al., 1990). For such data sets it is possible to consider a model which has the adult survival probability, ϕ_a, and the adult recovery probability, λ_a, common to both data sets. For the juveniles data, the model could also have a separate survival probability for first year birds, ϕ_1, and a separate recovery probability for first year birds, λ_1. The probabilities of recovery for birds marked as juveniles and birds marked as adults respectively are,

$$P_{i,j,juv} = \begin{cases} (1-\phi_1)\lambda_1 & i=j \\ \phi_1\phi_a^{j-i-1}(1-\phi_a) & j>i \end{cases} \quad P_{i,j,adu} = \phi_a^{j-i}(1-\phi_a)\lambda_a.$$

An exhaustive summary can be formed from the natural logarithm of these probabilities. If there have been three years of marking and three years of recovery the exhaustive summaries for the bird marked as juveniles are

$$\kappa_1 = \begin{bmatrix} \ln\{(1-\phi_1)\lambda_1\} \\ \ln\{\phi_1(1-\phi_a)\lambda_a\} \\ \ln\{\phi_1\phi_a(1-\phi_a)\lambda_a\} \end{bmatrix} \text{ and } \kappa_2 = \begin{bmatrix} \ln\{(1-\phi_a)\lambda_a\} \\ \ln\{\phi_a(1-\phi_a)\lambda_a\} \\ \ln\{\phi_a^2(1-\phi_a)\lambda_a\} \end{bmatrix}$$

respectively. These exhaustive summaries are based on the natural logarithm of the probabilities of recovery (Catchpole and Morgan, 1997) with any repeated exhaustive summary terms ignored. The parameters are $\theta_1 = [\phi_1, \phi_a, \lambda_1, \lambda_a]$ and $\theta_2 = [\phi_a, \lambda_a]$. If the adult data is analysed separately the model is full rank. However if the juvenile data sets are analysed separately the model is parameter redundant with rank 3 and deficiency 1 with estimable parameter combinations $s_{1,1} = \phi_a$, $s_{1,2} = \phi_1\lambda_a$ and $s_{1,3} = \phi_1(1-\phi_1)$ (Cole et al., 2012). Rewriting κ_2 in terms of $\mathbf{s}_1 = [s_{1,1}, s_{1,2}, s_{1,3}]$ gives,

$$\kappa_2(\mathbf{s}_1, \theta_2') = \begin{bmatrix} \ln\left\{\dfrac{(1-s_{1,1})s_{1,3}\lambda_1}{s_{1,2}-\lambda_1}\right\} \\ \ln\left\{\dfrac{s_{1,1}(1-s_{1,1})s_{1,3}\lambda_1}{s_{1,2}-\lambda_1}\right\} \\ \ln\left\{\dfrac{s_{1,1}^2(1-s_{1,1})s_{1,3}\lambda_1}{s_{1,2}-\lambda_1}\right\} \end{bmatrix}.$$

There is only one extra parameter, $\theta_2' = [\lambda_1]$. Therefore by remark 3 of the main paper the integrated model has rank 4. As the model also has 4 parameters the combined model is full rank. Adding 1 extra year of marking or 1 extra year of recovery adds no extra parameters, therefore by a trivial application of the extension theorem of Catchpole and Morgan (1997) and Cole et al. (2010) this integrated model is full rank for any number of years of marking and recovery.

It is also straightforward to use method 1 to prove the same result (see `Maple` code). The deficiency of other combined mark-recovery models are considered in Cole et al. (2012).

Example 8: Integrated mark-recovery and age dependent mixture models

Some mark-recovery data is collected on animals of unknown age. It is not possible using standard mark-recovery models to incorporate an age structure for survival probabilities or recovery probabilities. The paper McCrea et al. (2013) develops an age dependent mixture model that allow for this structure. An additional parameter, β_a, is introduced to represent the unobserved proportion of animals in each age class. However it is shown in McCrea et al. (2013) that these models are normally parameter redundant. The problem of parameter redundancy is overcome by integrating another data set where the full age structure of the animals is known.

© WILEY-VCH Verlag GmbH & Co. KGaA, Weinheim

Here we examine one such combined age-dependent mixture model. It is assumed there are two sets of data both covering 5 years of marking and 5 years of recovery. In the first set of data, animals are marked in their first year of life so that the full age structure is known. A standard mark-recovery model can be fitted to this set of data. The second set of data consists of animals marked at an unknown age. Suppose that we wish to fit a combined model to both data sets with separate survival probabilities for first year animals, ϕ_1, second year animals, ϕ_2, and adult animals, ϕ_a. However the second set of data could include second year animals, so we assume that the proportion β_2 of animals are in their second year. To allow for this age structure an age dependent mixture model is to be fitted the second data set. The recovery probability, $\lambda_{a,t}$ is assumed to be dependent on both the age category and time.

The probabilities of recovery for an animal marked in year i and recovered dead in year j for the first data set are $P_{1,i,j}$ and are summarised in the matrix below:

$$\mathbf{P}_1 = \begin{bmatrix} \bar{\phi}_1\lambda_{1,1} & \phi_1\bar{\phi}_2\lambda_{2,2} & \phi_1\phi_2\bar{\phi}_a\lambda_{a,3} & \phi_1\phi_2\phi_a\bar{\phi}_a\lambda_{a,4} & \phi_1\phi_2\phi_a^2\bar{\phi}_a\lambda_{a,5} \\ 0 & \bar{\phi}_1\lambda_{1,2} & \phi_1\bar{\phi}_2\lambda_{2,3} & \phi_1\phi_2\bar{\phi}_a\lambda_{a,4} & \phi_1\phi_2\phi_a\bar{\phi}_a\lambda_{a,4} \\ 0 & 0 & \bar{\phi}_1\lambda_{1,3} & \phi_1\bar{\phi}_2\lambda_{2,4} & \phi_1\phi_2\bar{\phi}_a\lambda_{a,5} \\ 0 & 0 & 0 & \bar{\phi}_1\lambda_{1,4} & \phi_1\bar{\phi}_2\lambda_{2,5} \\ 0 & 0 & 0 & 0 & \bar{\phi}_1\lambda_{1,5} \end{bmatrix},$$

with $\bar{x} = 1 - x$. The probabilities of recovery for an animal marked in year i and recovered dead in year j for the second data set are $P_{2,i,j}$ and are summarised in the matrices below:

$$\mathbf{P}_{2,1\ldots5,1\ldots3} = \begin{bmatrix} \beta_2\bar{\phi}_2\lambda_{2,1} + \bar{\beta}_2\bar{\phi}_a\lambda_{a,1} & \beta_2\phi_2\bar{\phi}_a\lambda_{a,2} + \bar{\beta}_2\phi_a\bar{\phi}_a\lambda_{a,2} & \beta_2\phi_2\phi_a\bar{\phi}_a\lambda_{a,3} + \bar{\beta}_2\phi_a^2\bar{\phi}_a\lambda_{a,3} \\ 0 & \beta_2\bar{\phi}_2\lambda_{2,2} + \bar{\beta}_2\bar{\phi}_a\lambda_{a,2} & \beta_2\phi_2\bar{\phi}_a\lambda_{a,3} + \bar{\beta}_2\phi_a\bar{\phi}_a\lambda_{a,3} \\ 0 & 0 & \beta_2\bar{\phi}_2\lambda_{2,3} + \bar{\beta}_2\bar{\phi}_a\lambda_{a,3} \\ 0 & 0 & 0 \\ 0 & 0 & 0 \end{bmatrix}$$

$$\mathbf{P}_{2,1\ldots5,4\ldots5} = \begin{bmatrix} \beta_2\phi_2\phi_a^2\bar{\phi}_a\lambda_{a,4} + \bar{\beta}_2\phi_a^3\bar{\phi}_a\lambda_{a,4} & \beta_2\phi_2\phi_a^3\bar{\phi}_a\lambda_{a,5} + \bar{\beta}_2\phi_a^4\bar{\phi}_a\lambda_{a,5} \\ \beta_2\phi_2\phi_a\bar{\phi}_a\lambda_{a,4} + \bar{\beta}_2\phi_a^2\bar{\phi}_a\lambda_{a,4} & \beta_2\phi_2\phi_a^2\bar{\phi}_a\lambda_{a,5} + \bar{\beta}_2\phi_a^3\bar{\phi}_a\lambda_{a,5} \\ \beta_2\phi_2\bar{\phi}_a\lambda_{a,4} + \bar{\beta}_2\phi_a\bar{\phi}_a\lambda_{a,4} & \beta_2\phi_2\phi_a\bar{\phi}_a\lambda_{a,5} + \bar{\beta}_2\phi_a^2\bar{\phi}_a\lambda_{a,5} \\ \beta_2\bar{\phi}_2\lambda_{2,4} + \bar{\beta}_2\bar{\phi}_a\lambda_{a,4} & \beta_2\phi_2\bar{\phi}_a\lambda_{a,5} + \bar{\beta}_2\phi_a\bar{\phi}_a\lambda_{a,5} \\ 0 & \beta_2\bar{\phi}_2\lambda_{2,5} + \bar{\beta}_2\bar{\phi}_a\lambda_{a,5} \end{bmatrix}.$$

The exhaustive summaries κ_1 and κ_2 consist of the natural logarithm of the non-zero entries of $\mathbf{P}_1$ and $\mathbf{P}_2$. It is not possible to use method 1 to find the rank of the integrated mark-recovery and age dependent mixture model as `Maple` runs out of memory trying to calculate the rank. One possibility is to use the hybrid symbolic-numeric method of Choquet and Cole (2012), as demonstrated in the `Maple` code.

The alternative is to use method 2. The vector κ_1 has rank $q_1 = 13$, but 15 parameters so had deficiency 2. Here we use the reparameterisation, $\mathbf{s}_1$, consisting of ϕ_a, $P_{i,i}$ for $i = 1,\ldots,5$ $P_{i,i+1}$ for $i = 1,\ldots,4$ and $P_{1,j}$ for $i = 3,\ldots,5$. Next κ_2 is rewritten terms of $\mathbf{s}_1$, which has the additional parameters $\theta_2' = [\beta_2, \lambda_{1,4}, \lambda_{2,1}, \lambda_{2,4}, \lambda_{a,1}, \lambda_{a,2}]$ and then the derivative matrix $\mathbf{D}_{2,2} = \partial\kappa_2(\mathbf{s}_1, \theta_2')/\partial\theta_2'$ is formed. It is shown in the `Maple` code that the rank of $\mathbf{D}_{2,2}$ is $r' = 4$. By Theorem 2 the rank of the integrated model is $q_1 + r' = 13 + 4 = 17$. However there are 19 parameters so this model is parameter redundant with deficiency 2. It is not possible to estimate all the parameters of this model.

Full parameter redundancy results for other integrated mark-recovery and age dependent mixture models are given in McCrea et al. (2013).

Web Appendix C

In this section we examine mark-recovery, capture-recapture, and capture-recapture-recovery models in state-space model format.

© WILEY-VCH Verlag GmbH & Co. KGaA, Weinheim

The mark-recovery model discussed in examples 6 and 7 can be written as a state-space model with

$$Y_{i,t}|X_{i,t},X_{i,t-1} \sim \text{Bernoulli}\{(X_{i,t-1}-X_{i,t})\lambda_t\} \text{ and } X_{i,t+1}|X_{i,t} \sim \text{Bernoulli}(X_{i,t}\phi_{t+1}),$$

where for individual $i = 1,\dots,n$, $X_{i,t}$ is a binary random variable taking values 1 if the individual is alive at time $t = 1,\dots,T$ and 0 otherwise, $Y_{i,t}$ is a binary random variable taking on values 1 if the individual is recovered dead at time t and 0 otherwise. The parameter ϕ_t is the probability of survival and the parameter λ_t is the probability of recovery. As all calculations are made conditional on when individual i was marked at time $t = f_i$ we assume $X_{i,f_i-1} = 1$ (Gimenez et al., 2007). For individuals marked at time $t = 1$ the state-space exhaustive summary is formed from

$$\begin{array}{rclrcl} E(X_{i,1}) & = & \phi_1, & E(Y_{i,1}) & = & (1-\phi_1)\lambda_1 \\ E(X_{i,2}) & = & \phi_1\phi_2, & E(Y_{i,2}) & = & \phi_1(1-\phi_2)\lambda_2 \\ E(X_{i,3}) & = & \phi_1\phi_2\phi_3, & E(Y_{i,2}) & = & \phi_1\phi_2(1-\phi_3)\lambda_3 \\ \vdots & & & \vdots & & \end{array}$$

Similar terms can be derived from animals marked at later times. The $E(Y_{i,j})$ form an exhaustive summary with identical terms to the one used in Cole et al. (2012). Cole et al. (2012) also provide tables of general results for most common mark-recovery models.

The capture-recapture model can be written as a state-space model with

$$Y_{i,t}|X_{i,t},X_{i,t-1} \sim \text{Bernoulli}(X_{i,t}p_t) \text{ and } X_{i,t+1}|X_{i,t} \sim \text{Bernoulli}(X_{i,t}\phi_t),$$

where for individual $i = 1,\dots,n$, $X_{i,t}$ is a binary random variable taking values 1 if the individual is alive at time $t = 1,\dots,T$ and 0 otherwise, $Y_{i,t}$ is a binary random variable taking on values 1 if the individual is encountered at time t and 0 otherwise. The parameter ϕ_t is the probability of survival and the parameter λ_t is the probability of recovery. As all calculations are made conditional on when individual i was marked at time $t = f_i$ we assume $X_{i,f_i} = 1$ (Gimenez et al., 2007, Royle, 2008). For the exhaustive summary derived below for 4 years of marking and 4 subsequent years of recovery, $Y_{i,t}$ denotes all individuals marked at time i.

$$\kappa_1 = \begin{bmatrix} E(Y_{1,2}) \\ E(Y_{1,3}) \\ E(Y_{1,4}) \\ E(Y_{1,5}) \\ E(Y_{2,3}) \\ E(Y_{2,4}) \\ E(Y_{2,5}) \\ E(Y_{3,4}) \\ E(Y_{3,5}) \\ E(Y_{4,5}) \end{bmatrix} = \begin{bmatrix} \phi_1 p_2 \\ \phi_1\phi_2 p_3 \\ \phi_1\phi_2\phi_3 p_4 \\ \phi_1\phi_2\phi_3\phi_4 p_5 \\ \phi_2 p_3 \\ \phi_2\phi_3 p_4 \\ \phi_2\phi_3\phi_4 p_5 \\ \phi_3 p_4 \\ \phi_3\phi_4 p_5 \\ \phi_4 p_5 \end{bmatrix}.$$

This exhaustive summary consists of 10 terms. In general for $T-1$ years of marking and $T-1$ subsequent years of recovery the exhaustive summary consists of $\frac{1}{2}T^2 - \frac{1}{2}T$ terms.

A simpler exhaustive summary is given Hubbard et al. (2014) with,

$$\kappa_1 = \begin{bmatrix} \phi_1 p_2 \\ \phi_2(1-p_2) \\ \phi_2 p_3 \\ \phi_2(1-p_3) \\ \phi_3 p_4 \\ \phi_3(1-p_4) \\ \phi_4 p_5 \end{bmatrix}.$$

© WILEY-VCH Verlag GmbH & Co. KGaA, Weinheim

This exhaustive summary is simpler and consists of only 7 terms. In general for $T-1$ years of marking and $T-1$ subsequent years of recovery the exhaustive summary consists of $2T-3$ terms.

The exhaustive summary of Hubbard et al. (2014) is also more general than the exhaustive summary produced when a capture-recapture model is written in terms of a state-space model, because the Hubbard et al. (2014) exhaustive summary allows for age dependence. It is better to use the simplest exhaustive summary available for any given model, therefore we recommend the use of the (Hubbard et al., 2014) exhaustive summary in this case. We also note that (Hubbard et al., 2014) gives general parameter results for many capture-recapture models.

Similarly simpler exhaustive summaries and general results are given for capture-recapture-recovery models in Hubbard et al. (2014).

References

Bellman,R. and Aström, K. J. (1970) On structural identifiability. *Mathematical Biosciences*, **7**, 329-339.

Besbeas, P., Freeman, S. N., Morgan, B. J. T. and Catchpole, E. A. (2002) Integrating Mark-Recapture-Recovery and Census Data to Estimate Animal Abundance and Demographic Parameters. *Biometrics*, **58**, 540-547.

Catchpole, E. A. and Morgan, B. J. T. (1997) Detecting parameter redundancy. *Biometrika*, **84**, 187-196.

Choquet, R., and Cole, D. J. (2012) A Hybrid Symbolic-Numerical Method for Determining Model Structure. *Mathematical Biosciences*, **236**, 117-125.

Cole, D. J., Morgan, B. J. T. and Titterington, D. M. (2010) Cole D. J., Morgan B. J. T, Titterington D (2010) Determining the parametric structure of models. *Mathematical Biosciences*, **228**, 16-30.

Cole, D. J., Morgan, B. J. T., Catchpole, E. A. and Hubbard, B. A. (2012) Parameter Redundancy of Mark-Recovery Models. *Biometrical Journal*, **54**, 507-523.

Freeman, S. N. and Morgan, B. J. T. (1990) Studies in the analysis of ring-recovery data. *The Ring*, **13**, 217-287.

Gimenez O., Rossi, V., Choquet, R., Dehais, C., Dorris, B., Varella, H. Vila, J. and Pradel, R. (2007). State-space modelling of data on marked individuals. *Ecological Modelling*, **206** 431-438.

Godfrey K. R. and Distefano, J. J. (1987) Identifiability of Model Parameters. In: Walter, E. (Ed.), Identifiability of Parametric Models, Pergamon Press, England, 1-20.

Graybill, F. A. (1969) Introduction to matrices with applications in statistics. Wadsworth Publishing Company, Belmont, California.

Hubbard, B. A., Cole, D. J. and Morgan, B. J .T. (2014) Parameter Redundancy in Capture-Recapture-Recovery Models. *Statistical Methodology*, **17**, 17-29.

Lebreton, J., Morgan, B. J. M., Pradel, R., Freeman, S. N. (1995) A Simultaneous Survival Rate Analysis of Dead Recovery and Live Recapture Data. Biometrics, **51**, 1418-1428.

McCrea, R. S., Morgan, B. J. T. and Cole, D. J. (2013) Age-dependent models for recovery data on animals marked at unknown age. *Applied Statistics*, **62**, 101-113.

Meyer, C. D. (1973) Generalized inverses and ranks of block matrices. *SIAM Journal of Applied Mathematics*, **25**, 597-602.

Pohjanpalo, H. (1978) System identifiability based on the power series of the solution. *Mathematical Biosciences*, **41**, 21-33.

Royle, J. A. (2008) Modeling Individual Effects in the Cormack-Jolly-Seber Model: A State-Space Formulation. *Biometrics*, **64**, 364-370.

Thowsen, A. (1978) Identifiability of dynamic systems. *International Journal of Systems Science*, **9**, 813-825.

© WILEY-VCH Verlag GmbH & Co. KGaA, Weinheim
