# Supplementary material for: Parameter redundancy in discrete state‐space and integrated models
Source: Biom J. 2016 Jun 30;58(5):1071–90. doi: 10.1002/bimj.201400239 (PMC5031231; doi:10.1002/bimj.201400239)
Supplement: Supplementary file 2 — Code [file BIMJ-58-1071-s002.zip › Example8.pdf]

```
> #Example 8 of Parameter Redundancy in Discrete State-Space and Integrated Models by D. J.
      Cole and R.S. McCrea
> with(LinearAlgebra) :
> Dmat :=proc(kappa, pars)
  local DD1, i, j;
  description "Form the derivative matrix";
  with(LinearAlgebra) :
  DD1 := Matrix(1 ..Dimension(pars), 1 ..Dimension(kappa) ) :
  for i from 1 to Dimension(pars)  do
      for j from 1 to Dimension(kappa) do
          DD1[i, j] := diff(kappa[j], pars[i])
      end do
   end do;
  DD1;
  end proc:
> Matvec :=proc(P)
   local sizekappa, i, j, kappa, kappaindex;
  description "Converts a Matrix into a Vector of the matrix's non-zero enteries";
  with(LinearAlgebra) : sizekappa := 0 :
  for i from 1 to Dimension(P)[1] do
     for j from 1 to Dimension(P)[2] do
         if (P[i, j] ≠ 0) then sizekappa := sizekappa + 1 : end if:
     end do
   end do;
  κ := Vector(sizekappa) : kappaindex := 0 :
  for i from 1 to Dimension(P)[1] do
     for j from 1 to Dimension(P)[2] do
         if (P[i, j] ≠ 0) then
            kappaindex :=  kappaindex + 1 : κ[kappaindex] := P[i, j] :
         end if:
     end do:
   end do: κ;
   end proc:
> Findalpha :=proc(DD1, pars)
  local r, d, alphapre, alpha, PDE, FF, i, ans;
  description "Finds α";
  with(LinearAlgebra) :
  r := Rank(DD1);
  d := Dimension(pars) - r :
  alphapre := NullSpace(Transpose(DD1) ) :
  α := Matrix(d, Dimension(pars) ) :
  for i from 1 to d do
        α[i, 1 ..Dimension(pars) ] := alphapre[i] :
   end do:
   α :
  end proc:
> Estpars :=proc(DD1, pars)
  local r, d, alphapre, alpha, PDE, FF, i, ans;
  description "Finds the estimable set of parameters";
  with(LinearAlgebra) :
```

```
r := Rank(DD1);
d := Dimension(pars) - r :
alphapre := NullSpace(Transpose(DD1)) :
α := Matrix(d, Dimension(pars)) : PDE := Vector(d) :
FF := f(seq(pars[i], i = 1 ..Dimension(pars))) :
for i from 1 to d do
    α[i, 1 ..Dimension(pars)] := alphapre[i] :
    PDE[i] := add(diff(FF, pars[j]) · α[i, j], j = 1 ..Dimension(pars)) :
 end do:
 ans := pdsolve({seq(PDE[i] = 0, i = 1 ..d)});
 end proc:
> logvector :=proc(A)
local i, lnA;
description "Finds ln of the entries of vector";
with(LinearAlgebra) :
 lnA := Vector(A);
for i from 1 to Dimension(A) do
   lnA[i] := ln(A[i]) :
 end do:
 lnA;
end proc:
> ringmod :=proc(x, y, z, r, c)
 local i, j, P, aa, b;
description "Finds P-array for x/y/z ring-recovery models. x first year survival, y adult
    survival, z reporting probability. 1=constant(C), 2=time(T), 3=age(A), 4=A,T ";
with(LinearAlgebra) :
P := Matrix(r, c) :
 if x = 1 then
    for i from 1 to c do
       aa[1, i] := phi[1] :
    end do:
 else
    for i from 1 to c do
       aa[1, i] := phi[1, i] :
    end do:
 end if:
 if y = 1 then
    for i from 2 to c do
       for j from 1 to c do
           aa[i, j] := phi[a] :
       end do:
    end do:
 elif y = 2 then
     for i from 2 to c do
       for j from 1 to c do
           aa[i, j] := phi[j] :
       end do:
    end do:
 elif y = 3 then
     for i from 2 to c do
       for j from 1 to c do
```

```
            aa[i, j] := phi[i] :
        end do:
    end do:
else
    for i from 2 to c do
        for j from 1 to c do
            aa[i, j] := phi[i, j] :
        end do:
    end do:
end if:
if z = 1 then
    for i from 1 to c do
        for j from 1 to c do
            b[i, j] := lambda :
        end do:
    end do:
elif z = 2 then
    for i from 1 to c do
        for j from 1 to c do
            b[i, j] := lambda[j] :
        end do:
    end do:
elif z = 3 then
    for i from 1 to c do
        for j from 1 to c do
            b[i, j] := lambda[i] :
        end do:
    end do:
else
    for i from 1 to c do
        for j from 1 to c do
            b[i, j] := lambda[i, j] :
        end do:
    end do:
end if:

for i from 1 to Dimension(P)[1] do
    for j from i to Dimension(P)[2] do
        P[i, j] := product(aa[k − i + 1, k], k = i..j − 1) · (1 − aa[j − i + 1, j]) · b[j − i + 1,
    j];
    end do:
end do:
P;
end proc:
> age := proc(r, c, As, Af)
local i, j, P, a;
description "Find the general P-array for r years of ringing and c years of recovery for ages As
    to Af";
with(LinearAlgebra) :
P := Matrix(r, c) :
for i from 1 to Dimension(P)[1] do
```

```
for j from i to Dimension(P)[2] do
        if i = j then
            for a from As to Af do
                P[i, j] := P[i, j] + β_{a, j}·(1 − ϕ_{a, j})·λ_{a, j}:
            end do:
        else
            for a from As to Af do
                P[i, j] := P[i, j] + β_{a, j}·product(ϕ_{a + k − i, k}, k = i..j − 1)·(1 − ϕ_{a + j − i, j})
·λ_{a + j − i, j}:
            end do:
        end if:
    end do:
end do:

P;
end proc:
```

```
>
> #Using method A
> n1 := 5 : n2 := 5 : J := 2 :
> P1 := ⟨eval(ringmod(2, 3, 4, n1, n2), {seq(seq(ϕ_{k, i} = ϕ_k, i = 1..20), k = 1..J), seq(ϕ_i = ϕ_a, i = J + 1..20), seq(seq(λ_{k, i} = λ_{k, i}, i = 1..20), k = 1..J), seq(seq(λ_{k, i} = λ_{a, i}, i = 1..20), k = J + 1..20)})⟩;
```

$$P1 := \left[\left[(1-\phi_1)\,\lambda_{1,1},\ \phi_1\,(1-\phi_2)\,\lambda_{2,2},\ \phi_1\,\phi_2\,(1-\phi_a)\,\lambda_{a,3},\ \phi_1\,\phi_2\,\phi_a\,(1-\phi_a)\,\lambda_{a,4},\ \phi_1\,\phi_2\,\phi_a^2\,(1-\phi_a)\,\lambda_{a,5}\right],\right. \tag{1}$$
$$\left[0,\ (1-\phi_1)\,\lambda_{1,2},\ \phi_1\,(1-\phi_2)\,\lambda_{2,3},\ \phi_1\,\phi_2\,(1-\phi_a)\,\lambda_{a,4},\ \phi_1\,\phi_2\,\phi_a\,(1-\phi_a)\,\lambda_{a,5}\right],$$
$$\left[0,\ 0,\ (1-\phi_1)\,\lambda_{1,3},\ \phi_1\,(1-\phi_2)\,\lambda_{2,4},\ \phi_1\,\phi_2\,(1-\phi_a)\,\lambda_{a,5}\right],$$
$$\left[0,\ 0,\ 0,\ (1-\phi_1)\,\lambda_{1,4},\ \phi_1\,(1-\phi_2)\,\lambda_{2,5}\right],$$
$$\left.\left[0,\ 0,\ 0,\ 0,\ (1-\phi_1)\,\lambda_{1,5}\right]\right]$$

```
> P2 := ⟨eval(age(n1, n2, 2, J + 1), {seq(seq(β_{k, i} = β_k, i = 1..20), k = 2..J), seq(β_{J + 1, i} = 1 − sum(β_k, k = 2..J), i = 1..20), seq(seq(ϕ_{k, i} = ϕ_k, i = 1..20), k = 1..J), seq(seq(ϕ_{k, i} = ϕ_a, i = 1..20), k = J + 1..10), seq(seq(λ_{k, i} = λ_{k, i}, i = 1..20), k = 1..J), seq(seq(λ_{k, i} = λ_{a, i}, i = 1..20), k = J + 1..20)})⟩ : P2[1..5, 1..3]; P2[1..5, 4..5];
```

$$\left[\left[\beta_2\,(1-\phi_2)\,\lambda_{2,1} + (1-\beta_2)\,(1-\phi_a)\,\lambda_{a,1},\ \beta_2\,\phi_2\,(1-\phi_a)\,\lambda_{a,2} + (1-\beta_2)\,\phi_a\,(1-\phi_a)\,\lambda_{a,2},\ \beta_2\,\phi_2\,\phi_a\,(1-\phi_a)\,\lambda_{a,3} + (1-\beta_2)\,\phi_a^2\,(1-\phi_a)\,\lambda_{a,3}\right],\right.$$
$$\left[0,\ \beta_2\,(1-\phi_2)\,\lambda_{2,2} + (1-\beta_2)\,(1-\phi_a)\,\lambda_{a,2},\ \beta_2\,\phi_2\,(1-\phi_a)\,\lambda_{a,3} + (1-\beta_2)\,\phi_a\,(1\right.$$

$-\phi_a)\,\lambda_{a,3}\Big],$

$\Big[0, 0, \beta_2\,(1-\phi_2)\,\lambda_{2,3} + (1-\beta_2)\,(1-\phi_a)\,\lambda_{a,3}\Big],$

$\Big[0, 0, 0\Big],$

$\Big[0, 0, 0\Big]\Big]$

$$\Big[\Big[\beta_2\,\phi_2\,\phi_a^2\,(1-\phi_a)\,\lambda_{a,4} + (1-\beta_2)\,\phi_a^3\,(1-\phi_a)\,\lambda_{a,4}, \beta_2\,\phi_2\,\phi_a^3\,(1-\phi_a)\,\lambda_{a,5} + (1-\beta_2)\,\phi_a^4\,(1-\phi_a)\,\lambda_{a,5}\Big], \tag{2}$$

$\Big[\beta_2\,\phi_2\,\phi_a\,(1-\phi_a)\,\lambda_{a,4} + (1-\beta_2)\,\phi_a^2\,(1-\phi_a)\,\lambda_{a,4}, \beta_2\,\phi_2\,\phi_a^2\,(1-\phi_a)\,\lambda_{a,5} + (1-\beta_2)\,\phi_a^3\,(1-\phi_a)\,\lambda_{a,5}\Big],$

$\Big[\beta_2\,\phi_2\,(1-\phi_a)\,\lambda_{a,4} + (1-\beta_2)\,\phi_a\,(1-\phi_a)\,\lambda_{a,4}, \beta_2\,\phi_2\,\phi_a\,(1-\phi_a)\,\lambda_{a,5} + (1-\beta_2)\,\phi_a^2\,(1-\phi_a)\,\lambda_{a,5}\Big],$

$\Big[\beta_2\,(1-\phi_2)\,\lambda_{2,4} + (1-\beta_2)\,(1-\phi_a)\,\lambda_{a,4}, \beta_2\,\phi_2\,(1-\phi_a)\,\lambda_{a,5} + (1-\beta_2)\,\phi_a\,(1-\phi_a)\,\lambda_{a,5}\Big],$

$\Big[0, \beta_2\,(1-\phi_2)\,\lambda_{2,5} + (1-\beta_2)\,(1-\phi_a)\,\lambda_{a,5}\Big]\Big]$

```
> kappa := Matvec(⟨P1, P2⟩) :
> pars := ⟨seq(op(i, indets(kappa)), i = 1 ..nops(indets(kappa)))⟩ :
> D1 := Dmat(kappa, pars) :
> #The computer runs out of memory trying to calculate the rank. It is possible using the
      symbolic hybrid symbolic-numeric method:
> results := Matrix(5, 1) :
for j from 1 to 5 do
    numpars := seq(pars[i] = evalf(rand() / 1000000000000), i = 1 ..Dimension(pars)) :
    D1rand := eval(D1, {numpars});
    results[j, 1] := Rank( D1rand);
end do:
results
```

$$\begin{bmatrix} 17 \\ 17 \\ 17 \\ 17 \\ 17 \end{bmatrix} \tag{3}$$

```
> #Therefore the rank is 17
>
>
> #Using method B
```

```
> n1 := 5 : n2 := 5 : J := 2 :
> P1 := ⟨eval(ringmod(2, 3, 4, n1, n2), {seq(seq(φ[k, i] = φ[k], i = 1 ..20), k = 1 ..J), seq(φ[i] = φ[a], i
      = J + 1 ..20), seq(seq(λ[k, i] = λ[k, i], i = 1 ..20), k = 1 ..J), seq(seq(λ[k, i] = λ[a, i], i = 1 ..20), k = J
      + 1 ..20)})⟩;
```

$$P1 := \left[\left[\left(1-\phi_1\right)\lambda_{1,1}, \phi_1\left(1-\phi_2\right)\lambda_{2,2}, \phi_1\phi_2\left(1-\phi_a\right)\lambda_{a,3}, \phi_1\phi_2\phi_a\left(1-\phi_a\right)\lambda_{a,4}, \phi_1\phi_2\phi_a^2\left(1-\phi_a\right)\lambda_{a,5}\right],\right.$$
$$\left[0, \left(1-\phi_1\right)\lambda_{1,2}, \phi_1\left(1-\phi_2\right)\lambda_{2,3}, \phi_1\phi_2\left(1-\phi_a\right)\lambda_{a,4}, \phi_1\phi_2\phi_a\left(1-\phi_a\right)\lambda_{a,5}\right],$$
$$\left[0, 0, \left(1-\phi_1\right)\lambda_{1,3}, \phi_1\left(1-\phi_2\right)\lambda_{2,4}, \phi_1\phi_2\left(1-\phi_a\right)\lambda_{a,5}\right],$$
$$\left[0, 0, 0, \left(1-\phi_1\right)\lambda_{1,4}, \phi_1\left(1-\phi_2\right)\lambda_{2,5}\right],$$
$$\left.\left[0, 0, 0, 0, \left(1-\phi_1\right)\lambda_{1,5}\right]\right] \quad (4)$$

```
> kappa1 := Matvec(P1) :
> pars1 := ⟨seq(op(i, indets(kappa1)), i = 1 ..nops(indets(kappa1)))⟩ :
> D1 := Dmat(kappa1, pars1) :
> r := Rank(D1); d := Dimension(pars1) − r;
```

$$r := 13$$
$$d := 2 \quad (5)$$

```
> # reparmeterisation
> s := ⟨φ[a], P1[1, 1], P1[2, 2], P1[3, 3], P1[4, 4], P1[5, 5], P1[1, 2], P1[2, 3], P1[3, 4],
      P1[4, 5], P1[1, 3], P1[1, 4], P1[1, 5]⟩ :
> #check reparameterisation theorem applies (should be 0):
> Dimension(s) - Rank(Dmat(s, pars1));
```

$$0 \quad (6)$$

```
> A := solve({seq(s[i] = ss[i], i = 1 ..Dimension(s))}, {seq(pars1[i], i = 1
      ..Dimension(pars1))}) :
> κ2 := Vector(kappa1) :
  for i from 1 to Dimension(kappa1) do
     κ2[i] := simplify(applyrule([seq(op(i, A), i = 1 ..nops(A))], kappa1[i])) :
  end do:
>
> Ds := Dmat(κ2, ⟨seq(ss[i], i = 1 ..Dimension(s))⟩) :
> r := Rank(Ds); d := Dimension(s) − r;
```

$$r := 13$$
$$d := 0 \quad (7)$$

```
> #s is a full rank reparameterisation so we can apply theorem 2
> P2 := ⟨eval(age(n1, n2, 2, J + 1), {seq(seq(β[k, i] = β[k], i = 1 ..20), k = 2 ..J), seq(β[J + 1, i] = 1
      − sum(β[k], k = 2 ..J), i = 1 ..20), seq(seq(φ[k, i] = φ[k], i = 1 ..20), k = 1 ..J), seq(seq(φ[k, i] = φ[a],
      i = 1 ..20), k = J + 1 ..10), seq(seq(λ[k, i] = λ[k, i], i = 1 ..20), k = 1 ..J), seq(seq(λ[k, i] = λ[a, i], i
```

```
        = 1 ..20), k = J + 1 ..20)})〉 :
> κ2 := Matvec(P2) :
> κ22 := Vector(κ2) :
  for i from 1 to Dimension(κ2) do
      κ22[i] := simplify(applyrule([seq(op(i, A), i = 1 ..nops(A))], κ2[i]));
  end do:
> indets(kappa22)
```

$$\{ss_1, ss_5, ss_7, ss_8, ss_9, ss_{10}, ss_{11}, ss_{12}, ss_{13}, \beta_2, \lambda_{1,4}, \lambda_{2,1}, \lambda_{2,4}, \lambda_{a,1}, \lambda_{a,2}\} \quad \textbf{(8)}$$

```
> par2dash := 〈β2, λ1,4, λ2,1, λ2,4, λa,1, λa,2〉 :
> D22 := Dmat(κ22, par2dash) :
> Rank(D22);
```

$$4 \quad \textbf{(9)}$$

```
> # Rank is 13+4=17, but there are 19 parameters in this model, therefore the deficeny is 2.
>
```
