# Supplementary material for: Parameter redundancy in discrete state‐space and integrated models
Source: Biom J. 2016 Jun 30;58(5):1071–90. doi: 10.1002/bimj.201400239 (PMC5031231; doi:10.1002/bimj.201400239)
Supplement: Supplementary file 2 — Code [file BIMJ-58-1071-s002.zip › Example1.pdf]

```
> #Example 1 of Parameter Redundancy in Discrete State-Space and Integrated Models by D. J.
      Cole and R.S. McCrea
> restart;
> with(LinearAlgebra) :
> Dmat :=proc(se, pars)
local DD1, i, j;
description "Form the derivative matrix";
with(LinearAlgebra) :
DD1 := Matrix(1 ..Dimension(pars), 1 ..Dimension(se)) :
for i from 1 to Dimension(pars)  do
    for j from 1 to Dimension(se) do
        DD1[i, j] := diff(se[j], pars[i])
    end do
 end do;
DD1;
 end proc:
> Estpars :=proc(DD1, pars)
local r, d, alphapre, alpha, PDE, FF, i, ans;
description "Finds the estimable set of parameters for derivative matrix DD1";
with(LinearAlgebra) :
r := Rank(DD1); d := Dimension(pars) - r :
alphapre := NullSpace(Transpose(DD1)) : α := Matrix(d, Dimension(pars)) : PDE
      := Vector(d) :
FF := f(seq(pars[i], i = 1 ..Dimension(pars))) :
for i from 1 to d do
      α[i, 1 ..Dimension(pars)] := alphapre[i] :
      PDE[i] := add(diff(FF, pars[j]) · α[i, j], j = 1 ..Dimension(pars)) :
 end do:
 ans := pdsolve({seq(PDE[i] = 0, i = 1 ..d)});
end proc:
> Estpars2 :=proc(DD1, pars)
local r, d, alphapre, alpha, PDE, FF, i, ans;
description "Finds the estimable set of parameters for derivative matrix DD1, and returns alpha
      and the PDEs";
with(LinearAlgebra) :
r := Rank(DD1); d := Dimension(pars) - r :
alphapre := NullSpace(Transpose(DD1)) : α := Matrix(d, Dimension(pars)) : PDE
      := Vector(d) :
FF := f(seq(pars[i], i = 1 ..Dimension(pars))) :
for i from 1 to d do
      α[i, 1 ..Dimension(pars)] := alphapre[i] :
      PDE[i] := add(diff(FF, pars[j]) · α[i, j], j = 1 ..Dimension(pars)) :
 end do:
 ans := ⟨pdsolve({seq(PDE[i] = 0, i = 1 ..d)}), {alpha}, {PDE}⟩;
end proc:
> ringmod2 :=proc(y, z, r, c)
 local i, j, P, aa, b;
description "Finds P-array for y/z ring-recovery models.  y survival, z reporting probability. 1=
      constant(C), 2=time(T), 3=age(A), 4=A,T ";
with(LinearAlgebra) :
```

```
P := Matrix(r, c) :
 if y = 1 then
    for i from 1 to c do   for j from 1 to c do  aa[i, j] := phi :          end do: end do:
 elif y = 2 then
     for i from 1 to c do  for j from 1 to c do   aa[i, j] := phi[j] :   end do: end do:
 elif y = 3 then
     for i from 1 to c do for j from 1 to c do  aa[i, j] := phi[i] :    end do: end do:
 else
    for i from 1 to c do  for j from 1 to c do  aa[i, j] := phi[i, j] : end do: end do:
 end if:
  if z = 1 then
    for i from 1 to c do for j from 1 to c do  b[i, j] := lambda :    end do: end do:
 elif z = 2 then
     for i from 1 to c do for j from 1 to c do  b[i, j] := lambda[j] :  end do: end do:
 elif z = 3 then
     for i from 1 to c do for j from 1 to c do  b[i, j] := lambda[i] :  end do:  end do:
else
    for i from 1 to c do for j from 1 to c do  b[i, j] := lambda[i, j] : end do: end do:
 end if:
 for i from 1 to Dimension(P)[1] do          for j from i to Dimension(P)[2] do
       P[i, j] := product(aa[k − i + 1, k], k = i..j − 1) · (1 − aa[j − i + 1, j]) ·b[j − i + 1,
    j];
 end do:    end do:
 P;
 end proc:
> Matvec :=proc(P)
 local sizekappa, i, j, kappa, kappaindex;
description "Converts a Matrix into a Vector of the matrix's non-zero enteries";
with(LinearAlgebra) :  sizekappa := 0 :
for i from 1 to Dimension(P)[1] do
    for j from 1 to Dimension(P)[2] do
        if (P[i, j] ≠ 0) then  sizekappa := sizekappa + 1 :  end if:
    end do
 end do;
κ := Vector(sizekappa) : kappaindex := 0 :
for i from 1 to Dimension(P)[1] do
    for j from 1 to Dimension(P)[2] do
        if (P[i, j] ≠ 0) then
           kappaindex :=   kappaindex + 1 : κ[kappaindex] := P[i, j] :
        end if:
    end do:
 end do:  κ;
end proc:
> Expan :=proc(A, C, x0, n)
 local i, x, y, kappa, tt;
description "Finds the exhaustive summary for the expansion method with n terms";
 y := eval(Multiply(C, x0), t = 0);
 x := eval(Multiply(A, x0), t = 1);
 tt := 1 :
 kappa := ⟨ ⟩ :
  for i from 1 to n do
```

```
        y := Multiply(eval(C, t = tt), x);
        tt := tt + 1 :
        x := Multiply(eval(A, t = tt), x);
        kappa := ⟨kappa, y⟩;
    end do:
    kappa := convert(kappa, Vector)
    end proc:
> #The measurement matrix, transition matrix and vector of inital values:
> Z := ⟨0|1⟩ : A := ⟨⟨0|ρ·ϕ_1⟩, ⟨ϕ_a|ϕ_a⟩⟩ : x0 := ⟨x_{0, 1}, x_{0, 2}⟩ :
> #The exhaustive summary using option II. The proceedure Expan creates the exhaustive
    summary.
> kappa := simplify(Expan(A, Z, x0, 3));
```

$$\kappa := \begin{bmatrix} \phi_a x_{0,1} + \phi_a x_{0,2} \\ \phi_a \rho \phi_1 x_{0,2} + \phi_a^2 x_{0,1} + \phi_a^2 x_{0,2} \\ \phi_a^2 \rho \phi_1 x_{0,1} + 2 \phi_a^2 \rho \phi_1 x_{0,2} + \phi_a^3 x_{0,1} + \phi_a^3 x_{0,2} \end{bmatrix} \quad (1)$$

```
> #Vector of parameters:
> pars := ⟨ϕ_1, ϕ_a, ρ⟩ :
> #The procedure Dmat finds the derivative matrix:
> D1 := Dmat(kappa, pars);
```

$$D1 := \left[\left[0, \phi_a \rho x_{0,2}, \phi_a^2 \rho x_{0,1} + 2 \phi_a^2 \rho x_{0,2} \right], \left[x_{0,1} + x_{0,2}, \rho \phi_1 x_{0,2} + 2 \phi_a x_{0,1} + 2 \phi_a x_{0,2}, 2 \phi_a \rho \phi_1 x_{0,1} + 4 \phi_a \rho \phi_1 x_{0,2} + 3 \phi_a^2 x_{0,1} + 3 \phi_a^2 x_{0,2} \right], \left[0, \phi_a \phi_1 x_{0,2}, \phi_a^2 \phi_1 x_{0,1} + 2 \phi_a^2 \phi_1 x_{0,2} \right] \right] \quad (2)$$

```
> #The rank and deficency of the model:
> r := Rank(D1); d := Dimension(pars) − r;
```

$$r := 2$$
$$d := 1 \quad (3)$$

```
> #The procedures Estpars and Estpars2 find the estimable parameter combinations. Estpars2 also
    displays the null space and PDEs.
> Estpars(D1, pars);
```

$$\{ f(\phi_1, \phi_a, \rho) = \_F1(\phi_a, \rho \phi_1) \} \quad (4)$$

```
> Estpars2(D1, pars);
```

(5)

$$\left[\begin{array}{c} \left\{f(\phi_1, \phi_a, \rho) = \_F1(\phi_a, \rho\,\phi_1)\right\} \\ \left\{\left[\begin{array}{ccc} -\frac{\phi_1}{\rho} & 0 & 1 \end{array}\right]\right\} \\ \left\{\left[\begin{array}{c} -\frac{\left(\frac{\partial}{\partial \phi_1} f(\phi_1, \phi_a, \rho)\right)\phi_1}{\rho} + \frac{\partial}{\partial \rho} f(\phi_1, \phi_a, \rho) \end{array}\right]\right\} \end{array}\right] \quad (5)$$

> #The exhaustive summary using option III

> kappa := *simplify*(*Expan*(*A*, *Z*, *x0*, 2));

$$\kappa := \left[\begin{array}{c} \phi_a x_{0,1} + \phi_a x_{0,2} \\ \phi_a \rho\, \phi_1 x_{0,2} + \phi_a^2 x_{0,1} + \phi_a^2 x_{0,2} \end{array}\right] \quad (6)$$

> *pars* := $\langle \phi_1, \phi_a, \rho \rangle$: *D1* := *Dmat*(kappa, *pars*) : *r* := *Rank*(*D1*); *d* := *Dimension*(*pars*) − *r*; *Estpars*(*D1*, *pars*);

$$r := 2$$
$$d := 1$$
$$\left\{f(\phi_1, \phi_a, \rho) = \_F1(\phi_a, \rho\,\phi_1)\right\} \quad (7)$$

> #We reparameterise in terms of the estimable parameters.

> *kappa2* := *eval*$\left(\text{kappa}, \phi_1 = \frac{\beta}{\rho}\right)$;

$$\kappa 2 := \left[\begin{array}{c} \phi_a x_{0,1} + \phi_a x_{0,2} \\ \phi_a \beta x_{0,2} + \phi_a^2 x_{0,1} + \phi_a^2 x_{0,2} \end{array}\right] \quad (8)$$

> *pars* := $\langle \beta, \phi_a \rangle$:

> *D1* := *Dmat*(*kappa2*, *pars*);

$$D1 := \left[\begin{array}{cc} 0 & \phi_a x_{0,2} \\ x_{0,1} + x_{0,2} & \beta x_{0,2} + 2\,\phi_a x_{0,1} + 2\,\phi_a x_{0,2} \end{array}\right] \quad (9)$$

> *r* := *Rank*(*D1*); *d* := *Dimension*(*pars*) − *r*;

$$r := 2$$
$$d := 0 \quad (10)$$

> #By a trivial application of the extension theorem the reparameterised model is always full rank for T⩾2. Then by the reparameterisation theorem the original parameterisation must also have rank 2. Therefore this model parameter redundant with deficiency 1 for T⩾2.

>

> #The following Maple code looks at including Error Terms:

> #Deriving the exhaustive summary terms:

> $A2 := \langle\langle 0|\rho\cdot\phi_1\rangle, \langle\phi_a\cdot(1-\phi_a)|\phi_a\cdot(1-\phi_a)\rangle\rangle;$

$$A2 := \begin{bmatrix} 0 & \rho\,\phi_1 \\ \phi_a\,(1-\phi_a) & \phi_a\,(1-\phi_a) \end{bmatrix} \quad \textbf{(11)}$$

> $Varx_1 := Multiply(A2, x0) : Vary_1 := Multiply(Z, Varx_1) + \sigma^2;$

$$Vary_1 := \phi_a\,(1-\phi_a)\,x_{0,\,1} + \phi_a\,(1-\phi_a)\,x_{0,\,2} + \sigma^2 \quad \textbf{(12)}$$

> $Varx_2 := Multiply(A2, Varx_1) : x_2 := Multiply(A, Varx_1) : Vary_2 := Multiply(Z, Varx_2) + \sigma^2;$

$$Vary_2 := \phi_a\,(1-\phi_a)\,\rho\,\phi_1\,x_{0,\,2} + \phi_a\,(1-\phi_a)\,(\phi_a\,(1-\phi_a)\,x_{0,\,1} + \phi_a\,(1-\phi_a)\,x_{0,\,2}) + \sigma^2 \quad \textbf{(13)}$$

> $kappav := convert(\langle \text{kappa}, Vary_1, Vary_2\rangle, Vector);$

$$kappav := \begin{bmatrix} \phi_a\,x_{0,\,1} + \phi_a\,x_{0,\,2} \\ \phi_a\,\rho\,\phi_1\,x_{0,\,2} + \phi_a^2\,x_{0,\,1} + \phi_a^2\,x_{0,\,2} \\ \phi_a\,(1-\phi_a)\,x_{0,\,1} + \phi_a\,(1-\phi_a)\,x_{0,\,2} + \sigma^2 \\ \phi_a\,(1-\phi_a)\,\rho\,\phi_1\,x_{0,\,2} + \phi_a\,(1-\phi_a)\,(\phi_a\,(1-\phi_a)\,x_{0,\,1} + \phi_a\,(1-\phi_a)\,x_{0,\,2}) + \sigma^2 \end{bmatrix} \quad \textbf{(14)}$$

> #Vector of parameters:

> $pars := \langle\phi_1, \phi_a, \rho, \sigma\rangle :$

> #The procedure Dmat finds the derivative matrix:

> $Dv := Dmat(kappav, pars) :$

> #The rank and deficency of the model:

> $r := Rank(Dv); d := Dimension(pars) - r;$

$$r := 3$$

$$d := 1 \quad \textbf{(15)}$$

> #The model is parameter redundant with deficency 1

> #The estimable parameter combinations are:

> $Estpars(Dv, pars);$

$$\{f(\phi_1, \phi_a, \rho, \sigma) = \_F1(\phi_a, \rho\,\phi_1, \sigma)\} \quad \textbf{(16)}$$

>

> #Integrated population model combined with ring−recovery data. Method A.

> $\text{kappa} := simplify(Expan(A, Z, x0, 3)) : kappa1 := \text{kappa};$

$$\kappa 1 := \begin{bmatrix} \phi_a\,x_{0,\,1} + \phi_a\,x_{0,\,2} \\ \phi_a\,\rho\,\phi_1\,x_{0,\,2} + \phi_a^2\,x_{0,\,1} + \phi_a^2\,x_{0,\,2} \\ \phi_a^2\,\rho\,\phi_1\,x_{0,\,1} + 2\,\phi_a^2\,\rho\,\phi_1\,x_{0,\,2} + \phi_a^3\,x_{0,\,1} + \phi_a^3\,x_{0,\,2} \end{bmatrix} \quad \textbf{(17)}$$

> $kappa2 := Matvec(eval(ringmod2(3, 1, 3, 3), \{seq(\text{phi}[i] = \text{phi}[a], i = 2..10)\})) : kappa2$

$:= \langle kappa2[1], kappa2[2], kappa2[3] \rangle;$

$$\kappa 2 := \begin{bmatrix} (1-\phi_1)\,\lambda \\ \phi_1\,(1-\phi_a)\,\lambda \\ \phi_1\,\phi_a\,(1-\phi_a)\,\lambda \end{bmatrix} \quad (18)$$

> $pars := \langle \phi_1, \phi_a, \rho, \lambda \rangle :$

> $D1 := factor(simplify(Dmat(convert(\langle kappa1, kappa2 \rangle, Vector), pars)));$

$$D1 := [[0, \phi_a\,\rho\,x_{0,2}, \phi_a^2\,\rho\,x_{0,1} + 2\,\phi_a^2\,\rho\,x_{0,2}, -\lambda, -(-1+\phi_a)\,\lambda, -\phi_a\,(-1+\phi_a)\,\lambda], [x_{0,1} + x_{0,2}, \rho\,\phi_1\,x_{0,2} + 2\,\phi_a\,x_{0,1} + 2\,\phi_a\,x_{0,2}, 2\,\phi_a\,\rho\,\phi_1\,x_{0,1} + 4\,\phi_a\,\rho\,\phi_1\,x_{0,2} + 3\,\phi_a^2\,x_{0,1} + 3\,\phi_a^2\,x_{0,2}, 0, -\phi_1\,\lambda, \phi_1\,\lambda - 2\,\phi_1\,\phi_a\,\lambda], [0, \phi_a\,\phi_1\,x_{0,2}, \phi_a^2\,\phi_1\,x_{0,1} + 2\,\phi_a^2\,\phi_1\,x_{0,2}, 0, 0, 0], [0, 0, 0, 1-\phi_1, -\phi_1\,(-1+\phi_a), -\phi_1\,\phi_a\,(-1+\phi_a)]] \quad (19)$$

> $r := Rank(D1);\ d := Dimension(pars) - r;$

$$r := 4$$
$$d := 0 \quad (20)$$

>

> #Integrated population model combined with ring−recovery data. Method B.

> kappa $:= simplify(Expan(A, Z, x0, 3)) :\ kappaB1 :=$ kappa;

$$kappaB1 := \begin{bmatrix} \phi_a\,x_{0,1} + \phi_a\,x_{0,2} \\ \phi_a\,\rho\,\phi_1\,x_{0,2} + \phi_a^2\,x_{0,1} + \phi_a^2\,x_{0,2} \\ \phi_a^2\,\rho\,\phi_1\,x_{0,1} + 2\,\phi_a^2\,\rho\,\phi_1\,x_{0,2} + \phi_a^3\,x_{0,1} + \phi_a^3\,x_{0,2} \end{bmatrix} \quad (21)$$

> $pars1 := \langle \phi_1, \phi_a, \rho \rangle :$

> $D1 := Dmat(kappaB1, pars1);$

$$D1 := [[0, \phi_a\,\rho\,x_{0,2}, \phi_a^2\,\rho\,x_{0,1} + 2\,\phi_a^2\,\rho\,x_{0,2}], [x_{0,1} + x_{0,2}, \rho\,\phi_1\,x_{0,2} + 2\,\phi_a\,x_{0,1} + 2\,\phi_a\,x_{0,2}, 2\,\phi_a\,\rho\,\phi_1\,x_{0,1} + 4\,\phi_a\,\rho\,\phi_1\,x_{0,2} + 3\,\phi_a^2\,x_{0,1} + 3\,\phi_a^2\,x_{0,2}], [0, \phi_a\,\phi_1\,x_{0,2}, \phi_a^2\,\phi_1\,x_{0,1} + 2\,\phi_a^2\,\phi_1\,x_{0,2}]] \quad (22)$$

> $Estpars(D1, pars1)$

$$\{ f(\phi_1, \phi_a, \rho) = \_F1(\phi_a, \rho\,\phi_1) \} \quad (23)$$

> # We reparameterise in terms of the estimable parameter combinations

> $s1 := \langle \phi_a, \rho\,\phi_1 \rangle;$

$$s1 := \begin{bmatrix} \phi_a \\ \rho\,\phi_1 \end{bmatrix} \tag{24}$$

> #Rewriting $\boldsymbol{\kappa}_{B,2}$ in terms of $\boldsymbol{s}$:

> $A := solve(\{seq(ss1[i] = s1[i], i = 1..Dimension(s1))\}, \{seq(pars1[i], i = 1..3)\});$

$$A := \left\{\rho = \rho, \phi_1 = \frac{ss1_2}{\rho}, \phi_a = ss1_1\right\} \tag{25}$$

> $kappaB2 := simplify(eval(\text{kappa2, A}));$

$$kappaB2 := \begin{bmatrix} \frac{(\rho - ss1_2)\,\lambda}{\rho} \\ -\frac{ss1_2\,(-1 + ss1_1)\,\lambda}{\rho} \\ -\frac{ss1_2\,ss1_1\,(-1 + ss1_1)\,\lambda}{\rho} \end{bmatrix} \tag{26}$$

> $pars2 := \langle \lambda, \rho \rangle;$

$$pars2 := \begin{bmatrix} \lambda \\ \rho \end{bmatrix} \tag{27}$$

> $D2 := simplify(Dmat(kappaB2, pars2));$

$$D2 := \begin{bmatrix} \frac{\rho - ss1_2}{\rho} & -\frac{ss1_2\,(-1 + ss1_1)}{\rho} & -\frac{ss1_2\,ss1_1\,(-1 + ss1_1)}{\rho} \\ \frac{\lambda\,ss1_2}{\rho^2} & \frac{ss1_2\,(-1 + ss1_1)\,\lambda}{\rho^2} & \frac{ss1_2\,ss1_1\,(-1 + ss1_1)\,\lambda}{\rho^2} \end{bmatrix} \tag{28}$$

> $r := Rank(D2); d := Dimension(pars2) - r;$

$$r := 2$$

$$d := 0 \tag{29}$$

> #Therefore the integrated model is full rank with rank 2+2=4

>
