# Supplementary material for: Parameter redundancy in discrete state‐space and integrated models
Source: Biom J. 2016 Jun 30;58(5):1071–90. doi: 10.1002/bimj.201400239 (PMC5031231; doi:10.1002/bimj.201400239)
Supplement: Supplementary file 2 — Code [file BIMJ-58-1071-s002.zip › Example2.pdf]

```
> #Example 2 of Parameter Redundancy in Discrete State-Space and Integrated Models by D. J.
      Cole and R.S. McCrea
> restart;
> with(LinearAlgebra) :
> Dmat :=proc(se, pars)
local DD1, i, j;
description "Form the derivative matrix";
with(LinearAlgebra) :
DD1 := Matrix(1 ..Dimension(pars), 1 ..Dimension(se)) :
for i from 1 to Dimension(pars)  do
     for j from 1 to Dimension(se) do
          DD1[i, j] := diff(se[j], pars[i])
     end do
 end do;
DD1;
 end proc:
> Estpars :=proc(DD1, pars)
local r, d, alphapre, alpha, PDE, FF, i, ans;
description "Finds the estimable set of parameters for derivative matrix DD1";
with(LinearAlgebra) :
r := Rank(DD1);
d := Dimension(pars) - r :
alphapre := NullSpace(Transpose(DD1)) :
α := Matrix(d, Dimension(pars)) : PDE := Vector(d) :
FF := f(seq(pars[i], i = 1 ..Dimension(pars))) :
for i from 1 to d do
        α[i, 1 ..Dimension(pars)] := alphapre[i] :
        PDE[i] := add(diff(FF, pars[j])·α[i, j], j = 1 ..Dimension(pars)) :
 end do:
 ans := pdsolve({seq(PDE[i] = 0, i = 1 ..d)});
end proc:
> Expan :=proc(A, C, x0, n)
 local i, x, y, kappa, tt;
description "Finds the exhaustive summary for the expansion method with n terms";
 y := eval(Multiply(C, x0), t = 0);
 x := eval(Multiply(A, x0), t = 1);
 tt := 1 :
 kappa := ⟨ ⟩ :
  for i from 1 to n do
        y := Multiply(eval(C, t = tt), x);
         tt := tt + 1 :
        x := Multiply(eval(A, t = tt), x);
        kappa := ⟨kappa, y⟩;
 end do:
 kappa := convert(kappa, Vector)
 end proc:
> #The measurement matrix, transition matrix and vector of inital values:
> Z := ⟨0|1⟩ : A := ⟨⟨0|ρ·ϕ[1, t]⟩, ⟨ϕ[a]|ϕ[a]⟩⟩ : x0 := ⟨c[1], c[2]⟩ :
> #The exhaustive summary:
```

> kappa := *simplify*(*Expan*(*A*, *Z*, *x0*, 3));

$$\kappa := \begin{bmatrix} \phi_a c_1 + \phi_a c_2 \\ \phi_a \rho \phi_{1,1} c_2 + \phi_a^2 c_1 + \phi_a^2 c_2 \\ \phi_a^2 \rho \phi_{1,2} c_1 + \phi_a^2 \rho \phi_{1,2} c_2 + \phi_a^2 \rho \phi_{1,1} c_2 + \phi_a^3 c_1 + \phi_a^3 c_2 \end{bmatrix} \quad (1)$$

> #Vector of parameters:

> $pars := \langle \rho, \phi_a, \phi_{1,1}, \phi_{1,2} \rangle$:

> #The procedure Dmat finds the derivative matrix:

> $D1 := Dmat(\text{kappa}, pars)$:

> #The rank and deficency of the model:

> $r := Rank(D1); d := Dimension(pars) - r;$

$$r := 3$$
$$d := 1 \quad (2)$$

> #The model is parameter redundant with deficency 1

> #The estimable parameter combinations are:

> $Estpars(D1, pars);$

$$\{ f(\rho, \phi_a, \phi_{1,1}, \phi_{1,2}) = \_F1(\phi_a, \phi_{1,1}\rho, \phi_{1,2}\rho) \} \quad (3)$$

> #Reparameterising in terms of the estimable parameter combinations:

> $parsbeta := \langle \phi_a, \nu_1, \nu_2 \rangle$:

> $kappa2 := eval\left(\text{kappa}, \left\{ seq\left(\phi_{1,i} = \frac{\nu_i}{\rho}, i = 1..2 \right) \right\} \right);$

$$\kappa 2 := \begin{bmatrix} \phi_a c_1 + \phi_a c_2 \\ \phi_a \nu_1 c_2 + \phi_a^2 c_1 + \phi_a^2 c_2 \\ \phi_a^2 \nu_2 c_1 + \phi_a^2 \nu_2 c_2 + \phi_a^2 \nu_1 c_2 + \phi_a^3 c_1 + \phi_a^3 c_2 \end{bmatrix} \quad (4)$$

> $D2 := Dmat(kappa2, parsbeta)$:

> $r := Rank(D2); d := Dimension(parsbeta) - r;$

$$r := 3$$
$$d := 0 \quad (5)$$

> #The reparameterised model is full rank. Adding an extra year of data adds only one extra parameter $\nu_3 = \rho \phi_{1,3}$
therefore by a trivual application of the extension theorem this reparameterised model will always be full rank.
By the reparameterisation theorem the origional parameterisation also has rank $T-1$.
As the model has T parameters the origional model is parameter redundant with deficency 1.

>
