# Supplementary material for: Parameter redundancy in discrete state‐space and integrated models
Source: Biom J. 2016 Jun 30;58(5):1071–90. doi: 10.1002/bimj.201400239 (PMC5031231; doi:10.1002/bimj.201400239)
Supplement: Supplementary file 2 — Code [file BIMJ-58-1071-s002.zip › Example3.pdf]

```
> #Example 3 of Parameter Redundancy in Discrete State-Space and Integrated Models by D. J.
      Cole and R.S. McCrea
> with(LinearAlgebra) :
> Dmat := proc(se, pars)
  local DD1, i, j;
  description "Form the derivative matrix";
  with(LinearAlgebra) :
  DD1 := Matrix(1 ..Dimension(pars), 1 ..Dimension(se)) :
  for i from 1 to Dimension(pars)  do
      for j from 1 to Dimension(se) do
          DD1[i, j] := diff(se[j], pars[i])
      end do
   end do;
  DD1;
   end proc:
> Estpars := proc(DD1, pars)
  local r, d, alphapre, alpha, PDE, FF, i, ans;
  description "Finds the estimable set of parameters for derivative matrix DD1";
  with(LinearAlgebra) :
  r := Rank(DD1);
  d := Dimension(pars) - r :
  alphapre := NullSpace(Transpose(DD1)) :
  α := Matrix(d, Dimension(pars)) : PDE := Vector(d) :
  FF := f(seq(pars[i], i = 1 ..Dimension(pars))) :
  for i from 1 to d do
          α[i, 1 ..Dimension(pars)] := alphapre[i] :
          PDE[i] := add(diff(FF, pars[j])·α[i, j], j = 1 ..Dimension(pars)) :
   end do:
   ans := pdsolve({seq(PDE[i] = 0, i = 1 ..d)});
  end proc:
> #Deriving the exhaustive summary terms:
> x1 := x0·exp(a + b·log(x0)) :
> y1 := x1;
```

$$y_1 := x_0\,\mathrm{e}^{a+b\ln(x_0)} \qquad (1)$$

```
> x2 := x1·exp(a + b·log(x1)) :
> y2 := x2;
```

$$y_2 := x_0\,\mathrm{e}^{a+b\ln(x_0)}\,\mathrm{e}^{a+b\ln\left(x_0\,\mathrm{e}^{a+b\ln(x_0)}\right)} \qquad (2)$$

```
> x3 := x2·exp(a + b·log(x2)) :
> y3 := x3;
```

$$y_3 := x_0\,\mathrm{e}^{a+b\ln(x_0)}\,\mathrm{e}^{a+b\ln\left(x_0\,\mathrm{e}^{a+b\ln(x_0)}\right)}\,\mathrm{e}^{a+b\ln\left(x_0\,\mathrm{e}^{a+b\ln(x_0)}\,\mathrm{e}^{a+b\ln\left(x_0\,\mathrm{e}^{a+b\ln(x_0)}\right)}\right)} \qquad (3)$$

```
> kappa := ⟨y1, y2, y3⟩;
```

$$\kappa := \left[\begin{array}{c} x_0 e^{a+b\ln(x_0)} \\ x_0 e^{a+b\ln(x_0)} e^{a+b\ln\left(x_0 e^{a+b\ln(x_0)}\right)} \\ x_0 e^{a+b\ln(x_0)} e^{a+b\ln\left(x_0 e^{a+b\ln(x_0)}\right)} e^{a+b\ln\left(x_0 e^{a+b\ln(x_0)} e^{a+b\ln\left(x_0 e^{a+b\ln(x_0)}\right)}\right)} \end{array}\right] \quad (4)$$

> #The vector of parameters:

> $pars := \langle x_0, a, b\rangle$:

> #The procedure Dmat finds the derivative matrix:

> $D1 := Dmat(\text{kappa}, pars)$:

> #The rank and deficency of the model:

> $r := Rank(D1); d := Dimension(pars) - r;$

$$r := 3$$

$$d := 0 \quad (5)$$

> # A modified PLUR decomposition (or Turing factorisation) of D1, and finding the determinant of *u1*. This checks whether the model is parameter redundant at any points in the parameter space

$(pp, ll, u1, r1) := LUDecomposition(D1, output = ['P','L','U1','R'])$:

$DetU := Determinant(u1); solve(DetU = 0);$

$$DetU := \left(e^{a+b\ln(x_0)} + b\, e^{a+b\ln(x_0)}\right) x_0 e^{a+b\ln(x_0)} e^{a+b\ln\left(x_0 e^{a+b\ln(x_0)}\right)} \Bigg(-x_0 e^{a+b\ln(x_0)} e^{a+b\ln\left(x_0 e^{a+b\ln(x_0)}\right)} e^{a+b\ln\left(x_0 e^{a+b\ln(x_0)} e^{a+b\ln\left(x_0 e^{a+b\ln(x_0)}\right)}\right)} \ln\left(x_0 e^{a+b\ln(x_0)}\right) + x_0 e^{a+b\ln(x_0)} e^{a+b\ln\left(x_0 e^{a+b\ln(x_0)}\right)} e^{a+b\ln\left(x_0 e^{a+b\ln(x_0)} e^{a+b\ln\left(x_0 e^{a+b\ln(x_0)}\right)}\right)} \ln\left(x_0 e^{a+b\ln(x_0)} e^{a+b\ln\left(x_0 e^{a+b\ln(x_0)}\right)}\right)\Bigg)$$

$$\{a = a, b = -1, x_0 = x_0\}, \left\{a = a, b = b, x_0 = e^{-\frac{a}{b}}\right\} \quad (6)$$

> # The model is parameter redundant if b=−1 or $x_0 = e^{-\frac{a}{b}}$

>
