# Supplementary material for: Parameter redundancy in discrete state‐space and integrated models
Source: Biom J. 2016 Jun 30;58(5):1071–90. doi: 10.1002/bimj.201400239 (PMC5031231; doi:10.1002/bimj.201400239)
Supplement: Supplementary file 2 — Code [file BIMJ-58-1071-s002.zip › Example4.pdf]

```
> #Example 4 of Parameter Redundancy in Discrete State-Space and Integrated Models by D. J.
      Cole and R.S. McCrea
> restart :
> with(LinearAlgebra) :
> Dmat :=proc(se, pars)
  local DD1, i, j;
  description "Form the derivative matrix";
  with(LinearAlgebra) :
  DD1 := Matrix(1 ..Dimension(pars), 1 ..Dimension(se)) :
  for i from 1 to Dimension(pars)  do
      for j from 1 to Dimension(se) do
          DD1[i, j] := diff(se[j], pars[i])
      end do
  end do;
  DD1;
  end proc:
> Estpars :=proc(DD1, pars)
  local r, d, alphapre, alpha, PDE, FF, i, ans;
  description "Finds the estimable set of parameters for derivative matrix DD1";
  with(LinearAlgebra) :
  r := Rank(DD1);
  d := Dimension(pars) - r :
  alphapre := NullSpace(Transpose(DD1)) :
  α := Matrix(d, Dimension(pars)) : PDE := Vector(d) :
  FF := f(seq(pars[i], i = 1 ..Dimension(pars))) :
  for i from 1 to d do
        α[i, 1 ..Dimension(pars)] := alphapre[i] :
        PDE[i] := add(diff(FF, pars[j])·α[i, j], j = 1 ..Dimension(pars)) :
  end do:
  ans := pdsolve({seq(PDE[i] = 0, i = 1 ..d)});
  end proc:
> Estpars2 :=proc(DD1, pars)
  local r, d, alphapre, alpha, PDE, FF, i, ans;
  description "Finds the estimable set of parameters for derivative matrix DD1, and returns alpha
      and the PDEs";
  with(LinearAlgebra) :
  r := Rank(DD1);
  d := Dimension(pars) - r :
  alphapre := NullSpace(Transpose(DD1)) :
  α := Matrix(d, Dimension(pars)) : PDE := Vector(d) :
  FF := f(seq(pars[i], i = 1 ..Dimension(pars))) :
  for i from 1 to d do
        α[i, 1 ..Dimension(pars)] := alphapre[i] :
        PDE[i] := add(diff(FF, pars[j])·α[i, j], j = 1 ..Dimension(pars)) :
  end do:
  ans := ⟨pdsolve({seq(PDE[i] = 0, i = 1 ..d)}), {alpha}, {PDE}⟩;
  end proc:
> Expan :=proc(A, C, x0, n)
  local i, x, y, kappa, tt;
  description "Finds the exhaustive summary for the expansion method with n terms";
```

```
y := eval(Multiply(C, x0), t = 0);
x := eval(Multiply(A, x0), t = 1);
tt := 1 :
kappa := ⟨ ⟩ :
for i from 1 to n do
    y := Multiply(eval(C, t = tt), x);
    tt := tt + 1 :
    x := Multiply(eval(A, t = tt), x);
    kappa := ⟨kappa, y⟩;
end do:
kappa := convert(kappa, Vector)
end proc:
> #The measurement matrix, transition matrix and vector of inital values:
> Z := ⟨⟨0|0|0|0|1|0⟩, ⟨0|0|0|0|0|1⟩⟩; A := ⟨⟨0|0|0|0|ρ1·φ1,1|0⟩, ⟨0|0|0|0|0|ρ2·φ1,2⟩, ⟨φ1,1|0|φ1,1
    ·(1 − π1)|0|0|0⟩, ⟨0|φ1,2|0| φ1,2·(1 − π2)|0|0⟩, ⟨0|0|φ1,1·π1|0|φ2,1·(1 − ψ1,2)|φ2,2
    ·ψ2,1⟩, ⟨0|0|0| φ1,2·π2| φ2,1·ψ1,2|φ2,2·(1 − ψ2,1)⟩⟩; x0 := ⟨seq(x0,i, i = 1 ..6)⟩ :
```

$$Z := \begin{bmatrix} 0 & 0 & 0 & 0 & 1 & 0 \\ 0 & 0 & 0 & 0 & 0 & 1 \end{bmatrix}$$

$$A := \begin{bmatrix} 0 & 0 & 0 & 0 & \rho_1 \phi_{1,1} & 0 \\ 0 & 0 & 0 & 0 & 0 & \rho_2 \phi_{1,2} \\ \phi_{1,1} & 0 & \phi_{1,1}\left(1-\pi_1\right) & 0 & 0 & 0 \\ 0 & \phi_{1,2} & 0 & \phi_{1,2}\left(1-\pi_2\right) & 0 & 0 \\ 0 & 0 & \phi_{1,1}\pi_1 & 0 & \phi_{2,1}\left(1-\psi_{1,2}\right) & \phi_{2,2}\psi_{2,1} \\ 0 & 0 & 0 & \phi_{1,2}\pi_2 & \phi_{2,1}\psi_{1,2} & \phi_{2,2}\left(1-\psi_{2,1}\right) \end{bmatrix} \quad \textbf{(1)}$$

```
> #The exhaustive summary using option III
> kappa := simplify(Expan(A, Z, x0, 5)) :
> pars := ⟨π1, π2, φ1,1, φ1,2, φ2,1, φ2,2, ψ1,2, ψ2,1, ρ1, ρ2⟩ :
> D1 := Dmat(kappa, pars) :
> #Hybrid-symbolic-numeric method
> results := Matrix(5, 1) :
for j from 1 to 5 do
    numpars := seq(indets(kappa)[i] = evalf(rand()/1000000000000), i = 1
      ..nops(indets(kappa))) :
    D1rand := eval(D1, {numpars});
    results[j, 1] := Rank(D1rand);
end do:
results
```

$$\begin{bmatrix} 10 \\ 10 \\ 10 \\ 10 \\ 10 \end{bmatrix} \quad \textbf{(2)}$$

```
> #Symbolic method using reparameterisation:
> s := Vector(10) :
```

$s[1] := \phi_{1,1}\pi_1 x_{0,3} + \phi_{2,1}\left(1 - \psi_{1,2}\right)x_{0,5} + \phi_{2,2}\psi_{2,1}x_{0,6}$:

$s[2] := \phi_{1,2}\pi_2 x_{0,4} + \phi_{2,1}\psi_{1,2}x_{0,5} + \phi_{2,2}\left(1 - \psi_{2,1}\right)x_{0,6}$:

$s[3] := \phi_{1,1}\pi_1\left(\phi_{1,1}x_{0,1} + \phi_{1,1}\left(1 - \pi_1\right)x_{0,3}\right)$:

$s[4] := \phi_{1,2}\pi_2\left(\phi_{1,2}x_{0,2} + \phi_{1,2}\left(1 - \pi_2\right)x_{0,4}\right)$:

$s[5] := \phi_{2,1}\left(1 - \psi_{1,2}\right)$:

$s[6] := \phi_{2,2}\psi_{2,1}$:

$s[7] := \phi_{2,1}\psi_{1,2}$:

$s[8] := \phi_{2,2}\left(1 - \psi_{2,1}\right)$:

$s[9] := \phi_{1,1}\pi_1\left(\rho_1\phi_{1,1}^2 x_{0,5} + \phi_{1,1}\left(1 - \pi_1\right)\left(\phi_{1,1}x_{0,1} + \phi_{1,1}\left(1 - \pi_1\right)x_{0,3}\right)\right)$:

$s[10] := \phi_{1,2}\pi_2\left(\rho_2\phi_{1,2}^2 x_{0,6} + \phi_{1,2}\left(1 - \pi_2\right)\left(\phi_{1,2}x_{0,2} + \phi_{1,2}\left(1 - \pi_2\right)x_{0,4}\right)\right)$:

```
> #check reparameterisation theorem applies (should be 0):
> Dimension(s) - Rank(Dmat(s, pars));
```

$$0 \quad \textbf{(3)}$$

```
> #Rewritting κ in terms of s
> AA := solve({seq(s[i] = ss[i], i = 1 ..Dimension(s))}, {seq(pars[i], i = 1
      ..Dimension(pars))}) :
> κ2 := Vector(Dimension(kappa)) :
  for i from 1 to 10 do
     κ2[i] := simplify(applyrule([seq(op(i, AA), i = 1 ..nops(AA))], kappa[i]));
  end do:
  for i from 1 to Dimension(kappa) do
  ⟨i|indets(κ2[i])⟩
  end do
```

$$\begin{bmatrix} 1 & \{ss_1\} \end{bmatrix}$$

$$\begin{bmatrix} 2 & \{ss_2\} \end{bmatrix}$$

$$\begin{bmatrix} 3 & \{ss_1, ss_2, ss_3, ss_5, ss_6\} \end{bmatrix}$$

$$\begin{bmatrix} 4 & \{ss_1, ss_2, ss_4, ss_7, ss_8\} \end{bmatrix}$$

$$\begin{bmatrix} 5 & \{ss_1, ss_2, ss_3, ss_4, ss_5, ss_6, ss_7, ss_8, ss_9\} \end{bmatrix}$$

$$\left[\begin{array}{cc} 6 & \{ss_1, ss_2, ss_3, ss_4, ss_5, ss_6, ss_7, ss_8, ss_{10}\} \end{array}\right]$$

$$\left[\begin{array}{cc} 7 & \{ss_1, ss_2, ss_3, ss_4, ss_5, ss_6, ss_7, ss_8, ss_9, ss_{10}, x_{0,1}, x_{0,3}, x_{0,5}, x_{0,6}\} \end{array}\right]$$

$$\left[\begin{array}{cc} 8 & \{ss_1, ss_2, ss_3, ss_4, ss_5, ss_6, ss_7, ss_8, ss_9, ss_{10}, x_{0,2}, x_{0,4}, x_{0,5}, x_{0,6}\} \end{array}\right]$$

$$\left[\begin{array}{cc} 9 & \{ss_1, ss_2, ss_3, ss_4, ss_5, ss_6, ss_7, ss_8, ss_9, ss_{10}, x_{0,1}, x_{0,2}, x_{0,3}, x_{0,4}, x_{0,5}, x_{0,6}\} \end{array}\right]$$

$$\left[\begin{array}{cc} 10 & \{ss_1, ss_2, ss_3, ss_4, ss_5, ss_6, ss_7, ss_8, ss_9, ss_{10}, x_{0,1}, x_{0,2}, x_{0,3}, x_{0,4}, x_{0,5}, x_{0,6}\} \end{array}\right] \quad \textbf{(4)}$$

\>

\> $parss := \langle seq(ss[i], i = 1..Dimension(s))\rangle$:

\> #Using the extension theorem

\> $parss1 := \langle ss_1, ss_2\rangle$:

\> $kappas1 := \langle kappa2[1], kappa2[2]\rangle$:

\> $Ds1 := Dmat(kappas1, parss1)$: $Rank(Ds1)$;

$$2 \quad \textbf{(5)}$$

\> $parss2 := \langle seq(ss[i], i = 3..Dimension(s))\rangle$:

\> $kappas2 := convert(kappa2[3..10], Vector)$:

\> $Ds2 := Dmat(kappas2, parss2)$: $Rank(Ds2)$;

$$8 \quad \textbf{(6)}$$

\> # Therefore Ds will be of full rank 10. Therefore by the reparameterisation Theorem of Cole et al (2010) the origional parameterisation will also have full rank 10

\>
