# Supplementary material for: Parameter redundancy in discrete state‐space and integrated models
Source: Biom J. 2016 Jun 30;58(5):1071–90. doi: 10.1002/bimj.201400239 (PMC5031231; doi:10.1002/bimj.201400239)
Supplement: Supplementary file 2 — Code [file BIMJ-58-1071-s002.zip › Example5.pdf]

```
> #Example 5 of Parameter Redundancy in Discrete State-Space and Integrated Models by D. J.
      Cole and R.S. McCrea
> with(LinearAlgebra) :
> Dmat :=proc(se, pars)
  local DD1, i, j;
  description "Form the derivative matrix";
  with(LinearAlgebra) :
  DD1 := Matrix(1 ..Dimension(pars), 1 ..Dimension(se)) :
  for i from 1 to Dimension(pars)  do
     for j from 1 to Dimension(se) do
        DD1[i, j] := diff(se[j], pars[i])
     end do
   end do;
  DD1;
   end proc:
> Estpars :=proc(DD1, pars)
  local r, d, alphapre, alpha, PDE, FF, i, ans;
  description "Finds the estimable set of parameters for derivative matrix DD1";
  with(LinearAlgebra) :
  r := Rank(DD1);
  d := Dimension(pars) - r :
  alphapre := NullSpace(Transpose(DD1)) :
  α := Matrix(d, Dimension(pars)) : PDE := Vector(d) :
  FF := f(seq(pars[i], i = 1 ..Dimension(pars))) :
  for i from 1 to d do
        α[i, 1 ..Dimension(pars)] := alphapre[i] :
        PDE[i] := add(diff(FF, pars[j])·α[i, j], j = 1 ..Dimension(pars)) :
   end do:
   ans := pdsolve({seq(PDE[i] = 0, i = 1 ..d)});
  end proc:
>
> #The productivity data alone
> T := 8 :
> kappa1 := <seq(ρ_i·nn_i, i = 1 ..T)> :
> pars1 := <seq(ρ_i, i = 1 ..T)> :
> D1 := Dmat(κ1, pars1) :
> r := Rank(D1); d := Dimension(pars1) − r;
                                    r := 8
                                    d := 0                                    (1)
> # The model with productivity data alone will always be full rank with rank T. This result is
      obvious, but also results from a trivual application of the extension theorem as increasing T
      by 1 only adds one extra parameter.
>
> #The adult mark-recapture data alone:
> kappa2 := Vector(2·T − 1) :
> indexi := 1 :
  for i from 1 to T − 1 do
```

```
    kappa2[indexi] := φ_{a,i}·p_{a,i+1} :
    indexi := indexi + 1 :
   if i ≠ T − 1 then
      kappa2[indexi] := φ_{a,i}·(1 - p_{a,i+1}) :
      indexi := indexi + 1 :
   end if:
 end do:
> pars2 := ⟨seq(φ_{a,i}, i=1..T−1), seq(p_{a,i}, i=2..T)⟩ :
> D2 := Dmat(kappa2, pars2) :
> r := Rank(D2); d := Dimension(pars2) − r;
```

$$r := 13$$

$$d := 1 \qquad \textbf{(2)}$$

```
> Estpars(D2, pars2);
```

$$\left\{f\left(\phi_{a,1}, \phi_{a,2}, \phi_{a,3}, \phi_{a,4}, \phi_{a,5}, \phi_{a,6}, \phi_{a,7}, p_{a,2}, p_{a,3}, p_{a,4}, p_{a,5}, p_{a,6}, p_{a,7}, p_{a,8}\right) = \_F1\left(\phi_{a,1}, \phi_{a,2}, \phi_{a,3}, \phi_{a,4}, \phi_{a,5}, \phi_{a,6}, p_{a,2}, p_{a,3}, p_{a,4}, p_{a,5}, p_{a,6}, p_{a,7}, \phi_{a,7} p_{a,8}\right)\right\} \qquad \textbf{(3)}$$

```
> #The adult mark−recapture data alone are parameter redundant with d = 1. (See Cole et al,
      2010, for the extension theorem to prove this).
>
> #The Chick MRR data alone
> kappa3 := Vector(T^2 − 5/2 T + T (T − 1) + 3/2 − 1/2 (T − 1)^2) : indexi := 1 :
 for i from 1 to T − 1 do
    for j from i to T − 1 do
       kappa3[ indexi] := eval(ψ_{i,j}·τ_{i,j}·φ_{i,j}·q_{i,j}, {seq(seq(φ_{i,j} = φ_{0,j}, i = 1), j = 1..T),
     seq(seq(φ_{i,j} = φ_{i−1}, i = 2..4), j = 1..T), seq(seq(φ_{i,j} = φ_{a,j}, i = 5..T), j = 1..T),
     seq(seq(ψ_{i,j} = ψ, i = 4), j = 1..T), seq(seq(ψ_{i,j} = 1, i = 1..3), j = 1..T), seq(seq(ψ_{i,j} = 1, i
     = 5..T), j = 1..T), seq(seq(τ_{i,j} = 1, i = 1..4), j = 1..T), seq(seq(τ_{i,j} = τ, i = 5..T), j = 1
     ..T), seq(seq(q_{i,j} = 0 , i = 1), j = 1..T), seq(seq(q_{i,j} = q_i , i = 4), j = 1..T), seq(seq(q_{i,j}
     = q_a , i = 5...T), j = 1..T)}) :
       indexi := indexi + 1 :
    end do:
 end do:for i from 1 to T − 2 do
    for j from i to T − 2 do
       kappa3[ indexi] := eval(ψ_{i,j}·τ_{i,j}·φ_{i,j}·(1 - q_{i,j}), {seq(seq(φ_{i,j} = φ_{0,j}, i = 1), j = 1
     ..T), seq(seq(φ_{i,j} = φ_{i−1}, i = 2..4), j = 1..T), seq(seq(φ_{i,j} = φ_{a,j}, i = 5..T), j = 1..T),
     seq(seq(ψ_{i,j} = ψ, i = 4), j = 1..T), seq(seq(ψ_{i,j} = 1, i = 1..3), j = 1..T), seq(seq(ψ_{i,j} = 1, i
     = 5..T), j = 1..T), seq(seq(τ_{i,j} = 1, i = 1..4), j = 1..T), seq(seq(τ_{i,j} = τ, i = 5..T), j = 1
     ..T), seq(seq(q_{i,j} = 0 , i = 1), j = 1..T), seq(seq(q_{i,j} = q_i , i = 4), j = 1..T), seq(seq(q_{i,j}
     = q_a , i = 5...T), j = 1..T)}) :
```

```
            indexi := indexi + 1 :
        end do:
    end do:
    for i from 1 to T − 1 do
        for j from i to T − 1 do
            kappa3[ indexi] := eval(φ[i, j]·(1 − λ[j]), {seq(seq(φ[i, j] = φ[0, j], i = 1), j = 1 ..T),
        seq(seq(φ[i, j] = φ[i − 1], i = 2 ..4), j = 1 ..T), seq(seq(φ[i, j] = φ[a, j], i = 5 ..T), j = 1 ..T)}) :
            indexi := indexi + 1 :
        end do:
    end do:
> pars3 := ⟨seq(indets(κ3)[i], i = 1 ..nops(indets(κ3)))⟩ :
> D3 := Dmat(κ3, pars3) :
> r := Rank(D3); d := Dimension(pars3) − r;
                                r := 35
                                d := 0                                        (4)
> #Adding an extra year of data:
> kappa3ex := Vector(43) : indexi := 1 :
    for i from 1 to T − 1 do
        for j from T − 1 to T do
            kappa3ex[ indexi] := eval(ψ[i, j]·τ[i, j]·φ[i, j]·q[i, j], {seq(seq(φ[i, j] = φ[0, j], i = 1), j = 1 ..T),
        seq(seq(φ[i, j] = φ[i − 1], i = 2 ..4), j = 1 ..T), seq(seq(φ[i, j] = φ[a, j], i = 5 ..T), j = 1 ..T),
        seq(seq(ψ[i, j] = ψ, i = 4), j = 1 ..T), seq(seq(ψ[i, j] = 1, i = 1 ..3), j = 1 ..T), seq(seq(ψ[i, j] = 1, i
        = 5 ..T), j = 1 ..T), seq(seq(τ[i, j] = 1, i = 1 ..4), j = 1 ..T), seq(seq(τ[i, j] = τ, i = 5 ..T), j = 1
        ..T), seq(seq(q[i, j] = 0 , i = 1), j = 1 ..T), seq(seq(q[i, j] = q[i] , i = 4), j = 1 ..T), seq(seq(q[i, j]
        = q[a] , i = 5 ...T), j = 1 ..T)}) :
            indexi := indexi + 1 :
        end do:
    end do:for i from 1 to T − 2 do
        for j from T − 2 to T − 1 do
            kappa3ex[ indexi] := eval(ψ[i, j]·τ[i, j]·φ[i, j]·(1- q[i, j]), {seq(seq(φ[i, j] = φ[0, j], i = 1), j = 1
        ..T), seq(seq(φ[i, j] = φ[i − 1], i = 2 ..4), j = 1 ..T), seq(seq(φ[i, j] = φ[a, j], i = 5 ..T), j = 1 ..T),
        seq(seq(ψ[i, j] = ψ, i = 4), j = 1 ..T), seq(seq(ψ[i, j] = 1, i = 1 ..3), j = 1 ..T), seq(seq(ψ[i, j] = 1, i
        = 5 ..T), j = 1 ..T), seq(seq(τ[i, j] = 1, i = 1 ..4), j = 1 ..T), seq(seq(τ[i, j] = τ, i = 5 ..T), j = 1
        ..T), seq(seq(q[i, j] = 0 , i = 1), j = 1 ..T), seq(seq(q[i, j] = q[i] , i = 4), j = 1 ..T), seq(seq(q[i, j]
        = q[a] , i = 5 ...T), j = 1 ..T)}) :
            indexi := indexi + 1 :
        end do:
    end do:
    for i from 1 to T − 1 do
        for j from T − 1 to T do
            kappa3ex[ indexi] := eval(φ[i, j]·(1 − λ[j]), {seq(seq(φ[i, j] = φ[0, j], i = 1), j = 1 ..T),
        seq(seq(φ[i, j] = φ[i − 1], i = 2 ..4), j = 1 ..T), seq(seq(φ[i, j] = φ[a, j], i = 5 ..T), j = 1 ..T)}) :
```

```
            indexi := indexi + 1 :
        end do:
    end do:
    for i from T to T do
        for j from i to T do
            kappa3ex[ indexi] := eval(ψ[i, j]·τ[i, j]·ϕ[i, j]·q[i, j], {seq(seq(ϕ[i, j] = ϕ[0, j], i = 1), j = 1 ..T),
    seq(seq(ϕ[i, j] = ϕ[i - 1], i = 2 ..4), j = 1 ..T), seq(seq(ϕ[i, j] = ϕ[a, j], i = 5 ..T), j = 1 ..T),
    seq(seq(ψ[i, j] = ψ, i = 4), j = 1 ..T), seq(seq(ψ[i, j] = 1, i = 1 ..3), j = 1 ..T), seq(seq(ψ[i, j] = 1, i
    = 5 ..T), j = 1 ..T), seq(seq(τ[i, j] = 1, i = 1 ..4), j = 1 ..T), seq(seq(τ[i, j] = τ, i = 5 ..T), j = 1
    ..T), seq(seq(q[i, j] = 0 , i = 1), j = 1 ..T), seq(seq(q[i, j] = q[i] , i = 4), j = 1 ..T), seq(seq(q[i, j]
    = q[a] , i = 5 ...T), j = 1 ..T)}) :
            indexi := indexi + 1 :
        end do:
    end do:for i from T - 1 to T - 1 do
        for j from i to T - 1 do
            kappa3ex[ indexi] := eval(ψ[i, j]·τ[i, j]·ϕ[i, j]·(1 - q[i, j]), {seq(seq(ϕ[i, j] = ϕ[0, j], i = 1), j = 1
    ..T), seq(seq(ϕ[i, j] = ϕ[i - 1], i = 2 ..4), j = 1 ..T), seq(seq(ϕ[i, j] = ϕ[a, j], i = 5 ..T), j = 1 ..T),
    seq(seq(ψ[i, j] = ψ, i = 4), j = 1 ..T), seq(seq(ψ[i, j] = 1, i = 1 ..3), j = 1 ..T), seq(seq(ψ[i, j] = 1, i
    = 5 ..T), j = 1 ..T), seq(seq(τ[i, j] = 1, i = 1 ..4), j = 1 ..T), seq(seq(τ[i, j] = τ, i = 5 ..T), j = 1
    ..T), seq(seq(q[i, j] = 0 , i = 1), j = 1 ..T), seq(seq(q[i, j] = q[i] , i = 4), j = 1 ..T), seq(seq(q[i, j]
    = q[a] , i = 5 ...T), j = 1 ..T)}) :
            indexi := indexi + 1 :
        end do:
    end do:
    for i from T to T do
        for j from i to T do
            kappa3ex[ indexi] := eval(ϕ[i, j]·(1 - λ[j]), {seq(seq(ϕ[i, j] = ϕ[0, j], i = 1), j = 1 ..T),
    seq(seq(ϕ[i, j] = ϕ[i - 1], i = 2 ..4), j = 1 ..T), seq(seq(ϕ[i, j] = ϕ[a, j], i = 5 ..T), j = 1 ..T)}) :
            indexi := indexi + 1 :
        end do:
    end do:
> pars3ex := <ϕ[a, 8], λ[8], q[2, 8], q[3, 8], ϕ[0, 8]> :
> D3ex := Dmat(kappa3ex, pars3ex) : r := Rank(D3ex); d := Dimension(pars3ex) - r;
                                r := 5
                                d := 0                                        (5)
> # Therefore by the extension theorem the Chick MRR data alone are full rank with 5 T - 5
>
> #The state-space model alone:
> kappa4 := Vector(T - 5 + 1) :
> for i from 1 to 5 do
      x[i] := x[o, i] :
```

$$J_i := \frac{x_i \cdot \rho_i \cdot \phi_{0,\,i} \cdot \phi_1 \cdot \phi_2 \cdot \phi_3}{2}:$$

**end do**:
**for** $i$ **from** 6 **to** $T$ **do**

$w_i := x_{i-1} \cdot \phi_{a,\,i-1}:$

$z_i := J_{i-5} \cdot \mathrm{psi} \cdot \phi_{a,\,i-1}:$

$x_i := w_i + z_i:$

$y_i := x_i:$

$kappa4_{i-5} := y_i:$

**end do**:

$kappa4(T-5+1) := \sigma_N^2:$

> $kappa4$

$$\left[\left[x_{o,\,5}\,\phi_{a,\,5} + \frac{1}{2}\,x_{o,\,1}\,\rho_1\,\phi_{0,\,1}\,\phi_1\,\phi_2\,\phi_3\,\psi\,\phi_{a,\,5}\right],\right. \tag{6}$$

$$\left[\left(x_{o,\,5}\,\phi_{a,\,5} + \frac{1}{2}\,x_{o,\,1}\,\rho_1\,\phi_{0,\,1}\,\phi_1\,\phi_2\,\phi_3\,\psi\,\phi_{a,\,5}\right)\phi_{a,\,6} + \frac{1}{2}\,x_{o,\,2}\,\rho_2\,\phi_{0,\,2}\,\phi_1\,\phi_2\,\phi_3\,\psi\,\phi_{a,\,6}\right],$$

$$\left[\left(\left(x_{o,\,5}\,\phi_{a,\,5} + \frac{1}{2}\,x_{o,\,1}\,\rho_1\,\phi_{0,\,1}\,\phi_1\,\phi_2\,\phi_3\,\psi\,\phi_{a,\,5}\right)\phi_{a,\,6} + \frac{1}{2}\,x_{o,\,2}\,\rho_2\,\phi_{0,\,2}\,\phi_1\,\phi_2\,\phi_3\,\psi\,\phi_{a,\,6}\right)\phi_{a,\,7}\right.$$

$$\left.+ \frac{1}{2}\,x_{o,\,3}\,\rho_3\,\phi_{0,\,3}\,\phi_1\,\phi_2\,\phi_3\,\psi\,\phi_{a,\,7}\right],$$

$$\left.\left[\sigma_N^2\right]\right]$$

> $pars4 := \langle \psi, seq(\rho_i, i = 1..T-5), seq(\phi_{0,\,i}, i = 1..T-5), \phi_1, \phi_2, \phi_3, seq(\phi_{a,\,i}, i = 5..T-1), \sigma_N \rangle :$

> $D4 := Dmat(kappa4, pars4) :$

> $r := Rank(D4); d := Dimension(pars4) - r;$

$$r := 4$$
$$d := 10 \tag{7}$$

> #The state-space model is always parameter redundant limited by the number of exhaustive summary terms (see Cole et al, 2012 Biometrical Journal, for details of this method). The rank is T−4, as there are 3T−10 parameters this model is alway parameter redundant with deficency 2 T−6

>

> # Productivity data and Adult Mark-recapture data

> #Results by remark 2 as there are no parameters in common

>

> #Productivity data and Chick MMR data

> #Full rank by remark 1

>

> #Productivity data and state-space data

> #We use method B. As $\kappa_1$ is full rank the reparameterisation $s_1$ is just the origional

parameterisation, rank is $q_1 = T$

> $kappai2 := kappa4 : parsi2 := \langle \psi, seq(\phi_{0,i}, i=1..T-5), \phi_1, \phi_2, \phi_3, seq(\phi_{a,i}, i=5..T-1), \sigma_N \rangle :$

> $Di2 := Dmat(kappai2, parsi2) :$

> $r := Rank(Di2); d := Dimension(parsi2) - r;$

$$r := 4$$

$$d := 7 \tag{8}$$

> # $r_{ex} = T - 4$ (due to being limited by number of exhaustive summary terms), therefore model rank of this model is $q_1 + r_{ex} = 2\,T - 4$

>

> #Adult capture-recapture data and chick MRR data

> # Using method B. kappai1=$\kappa_3$ is full rank the reparameterisation $s_1$ is just the origional parameterisation, rank is $q_1 = 5\,T - 5$

> $kappai2 := kappa2 :$

> $indets(kappa2)$

$$\{p_{a,2}, p_{a,3}, p_{a,4}, p_{a,5}, p_{a,6}, p_{a,7}, p_{a,8}, \phi_{a,1}, \phi_{a,2}, \phi_{a,3}, \phi_{a,4}, \phi_{a,5}, \phi_{a,6}, \phi_{a,7}\} \tag{9}$$

> $indets(\kappa 3)$

$$\{\psi, \tau, \phi_1, \phi_2, \phi_3, \phi_{0,1}, \phi_{0,2}, \phi_{0,3}, \phi_{0,4}, \phi_{0,5}, \phi_{0,6}, \phi_{0,7}, \phi_{a,5}, \phi_{a,6}, \phi_{a,7}, q_4, q_a, q_{2,2}, q_{2,3}, q_{2,4}, q_{2,5}, q_{2,6}, q_{2,7}, q_{3,3}, q_{3,4}, q_{3,5}, q_{3,6}, q_{3,7}, \lambda_1, \lambda_2, \lambda_3, \lambda_4, \lambda_5, \lambda_6, \lambda_7\} \tag{10}$$

> $parsi2 := \langle seq(p_{a,i}, i=2..T), \phi_{a,1}, \phi_{a,2}, \phi_{a,3}, \phi_{a,4} \rangle :$

> $Di2 := Dmat(kappai2, parsi2) :$

> $r := Rank(Di2); d := Dimension(parsi2) - r;$

$$r := 11$$

$$d := 0 \tag{11}$$

> # Adding an extra year adds one extra parameter $p_{a,T+1}$.

# Therefore by a trivual application of extension theorem can show this model is full rank with rank $r_{ex} = T + 3$.
# Therefore the rank of this model is $q_1 + r_{ex} = 6\,T - 2$ :

>

> #Adult capture-recapture data and state-space model

> #Using method B. kappai1=$\kappa_2$ and use the reparameterisation of the estimable parameters $\phi_{a,1}, \ldots, \phi_{a,T-2}, p_{a,2}, \ldots, p_{a,T-1}, \ \phi_{T-1} p_T$. Rank is $q_1 = 2\,T - 3$

> kappai2 := kappa4 :

> $kappai2r := eval\left(kappai2, \phi_{a,T-1} = \frac{\beta}{p_{a,T-1}}\right) :$

> $parsi2 := \langle \psi, seq(\rho_i, i=1..T-5), seq(\phi_{0,i}, i=1..T-5), \phi_1, \phi_2, \phi_3, \sigma_N, p_{a,T-1} \rangle :$

> $Di2 := Dmat(kappai2r, parsi2) :$

> $r := Rank(Di2); d := Dimension(parsi2) - r;$

$$r := 4$$

$$d := 8 \qquad (12)$$

> #rank limited by number of exhuative summary terms so that $r_{ex} = T - 4$ therefore rank of this model is $q_1 + r_{ex} = 3\,T - 7$ . There are

# 4 T−7 parameters so the deficency is d=T

>

> #Chick MMR data and state-space data

> #Using method B. kappai1=$\kappa_3$ is of full rank $q_1 = 5\,T - 5$

> kappai2 := kappa4 :

> $parsi2 := \langle seq(\rho_i, i = 1..T-5), \sigma_N \rangle$ :

> $Di2 := Dmat(kappai2, parsi2)$ :

> $r := Rank(Di2); d := Dimension(parsi2) - r;$

$$r := 4$$

$$d := 0 \qquad (13)$$

> #By a trivual application of the extension theorem this will always be full rank with $r_{ex} = T - 4$ therefore rank of this model is $q_1 + r_{ex} = 6\,T - 9$

>

> #Productivity data, adult capture-recapture data and chick MMR data.

> #As dault capture-recapture and chick MMR together are full rank, and Productivity data alone is full rank by remark 1 this model is also full rank

>

> #Productivity data, adult capture-recapture and chick state-space model.

> # Using method B. kappai1=$\kappa_1$ is of full rank T

> kappai2 := convert( ⟨kappa2, kappa4⟩, *Vector*) :

> $parsi2 := \langle \psi, seq(p_{a,i}, i = 2..T), seq(\phi_{0,i}, i = 1..T-5), \phi_1, \phi_2, \phi_3, seq(\phi_{a,i}, i = 1..T-1), \sigma_N \rangle$ :

> $Di2 := Dmat(kappai2, parsi2)$ :

> $r := Rank(Di2); d := Dimension(parsi2) - r;$

$$r := 17$$

$$d := 5 \qquad (14)$$

> $s := \left\langle seq(p_{a,i}, i = 2..T-1), seq(\phi_{a,i}, i = 1..T-2), \phi_{a,T-1} \cdot p_{a,T}, \phi_{0,1}\,\phi_1\,\phi_2\,\phi_3\,\psi, \frac{\phi_{0,2}}{\phi_{0,1}}, \sigma_N, kappai2[18] \right\rangle;$

(15)

$$s := \left[\begin{array}{c} \text{1 .. 17 } Vector_{column} \\ \text{Data Type: anything} \\ \text{Storage: rectangular} \\ \text{Order: Fortran\_order} \end{array}\right] \quad (15)$$

```
> #check reparameterisation theorem applies (should be 0):
> Dimension(s) - Rank(Dmat(s, parsi2));
```

$$0 \quad (16)$$

```
> #Rewritting κ in terms of s
> AA := solve({seq(s[i] = ss[i], i = 1 ..Dimension(s))}, {seq(parsi2[i], i = 1
      ..Dimension(parsi2))}) :
> kappai2re := Vector(Dimension(kappai2)) :
  for i from 1 to 19 do
     kappai2re[i] := simplify(applyrule([seq(op(i, AA), i = 1 ..nops(AA))], kappai2[i]));
  end do;
```

$$kappai2re_1 := ss_7\, ss_1$$
$$kappai2re_2 := -ss_7\,(-1 + ss_1)$$
$$kappai2re_3 := ss_2\, ss_8$$
$$kappai2re_4 := -(-1 + ss_2)\, ss_8$$
$$kappai2re_5 := ss_3\, ss_9$$
$$kappai2re_6 := -(-1 + ss_3)\, ss_9$$
$$kappai2re_7 := ss_4\, ss_{10}$$
$$kappai2re_8 := -(-1 + ss_4)\, ss_{10}$$
$$kappai2re_9 := ss_5\, ss_{11}$$
$$kappai2re_{10} := -ss_{11}\,(-1 + ss_5)$$
$$kappai2re_{11} := ss_{12}\, ss_6$$
$$kappai2re_{12} := -ss_{12}\,(-1 + ss_6)$$
$$kappai2re_{13} := ss_{13}$$
$$kappai2re_{14} := 0$$
$$kappai2re_{15} := 0$$
$$kappai2re_{16} := x_{o,\,5}\, ss_{11} + \frac{1}{2}\, x_{o,\,1}\, \rho_1\, ss_{14}\, ss_{11}$$
$$kappai2re_{17} := ss_{12}\, x_{o,\,5}\, ss_{11} + \frac{1}{2}\, ss_{12}\, x_{o,\,1}\, \rho_1\, ss_{14}\, ss_{11} + \frac{1}{2}\, x_{o,\,2}\, \rho_2\, ss_{15}\, ss_{14}\, ss_{12}$$
$$kappai2re_{18} := ss_{17}$$
$$kappai2re_{19} := ss_{16}^2 \quad (17)$$

```
> parsi2s := ⟨seq(ss[i], i = 1 ..17)⟩ :
> Di2s := Dmat(kappai2re, parsi2s) :
> r := Rank(Di2s); d := Dimension(parsi2s) − r;
```

$$r := 17$$
$$d := 0 \quad \textbf{(18)}$$

> # The last reparameterisation term will always be the last state−space term. It can be shown using the extension theorem that

> #the rank is $r_{ex} = 3\,T - 7$, therefore the rank of this model is $q_1 + r_{ex} = 4\,T - 7$, $d = 5$

>

>

> #Productivity data, chick MRR and state-space data

> #As chick MRR and state-space model together are full rank, and the productivity data is full rank, by remark 1 the combined model is also full rank

>

> #Adult capture-recapture data and,chick MRR data and state−space data

> # Using method B. kappai1=$[\kappa_3, \kappa_4]$ is of full rank $q_1 = 6\,T - 9$

> kappai2 := kappa2 :

> $parsi2 := \langle seq(p_{a,i}, i = 2..T), seq(\phi_{a,i}, i = 1..4)\rangle$ :

> $Di2 := Dmat(kappai2, parsi2)$ :

> $r := Rank(Di2); d := Dimension(parsi2) - r;$

$$r := 11$$
$$d := 0 \quad \textbf{(19)}$$

> #By extension theorem this is also full rank with rank $r_{ex} = T + 3$, therefore the rank of this model is $q_1 + r_{ex} = 7\,T - 6$

> kappai2 := convert( ⟨kappa1, kappa2, *kappa3*, kappa4⟩ , *Vector*) : *indets*(*kappai2*)

$$\{\psi, \tau, nn_1, nn_2, nn_3, nn_4, nn_5, nn_6, nn_7, nn_8, p_{a,2}, p_{a,3}, p_{a,4}, p_{a,5}, p_{a,6}, p_{a,7}, p_{a,8}, \phi_1, \phi_2, \phi_3, \phi_{0,1}, \phi_{0,2}, \phi_{0,3}, \phi_{0,4}, \phi_{0,5}, \phi_{0,6}, \phi_{0,7}, \phi_{a,1}, \phi_{a,2}, \phi_{a,3}, \phi_{a,4}, \phi_{a,5}, \phi_{a,6}, \phi_{a,7}, q_4, q_a, q_{2,2}, q_{2,3}, q_{2,4}, q_{2,5}, q_{2,6}, q_{2,7}, q_{3,3}, q_{3,4}, q_{3,5}, q_{3,6}, q_{3,7}, \rho_1, \rho_2, \rho_3, \rho_4, \rho_5, \rho_6, \rho_7, \rho_8, x_{o,1}, x_{o,2}, x_{o,3}, x_{o,5}, \lambda_1, \lambda_2, \lambda_3, \lambda_4, \lambda_5, \lambda_6, \lambda_7, \sigma_N\} \quad \textbf{(20)}$$

> $parsi2 := \langle \psi, \tau, p_{a,2}, p_{a,3}, p_{a,4}, p_{a,5}, p_{a,6}, p_{a,7}, p_{a,8}, \phi_1, \phi_2, \phi_3, \phi_{0,1}, \phi_{0,2}, \phi_{0,3}, \phi_{0,4}, \phi_{0,5}, \phi_{0,6}, \phi_{0,7}, \phi_{a,1}, \phi_{a,2}, \phi_{a,3}, \phi_{a,4}, \phi_{a,5}, \phi_{a,6}, \phi_{a,7}, q_4, q_a, q_{2,2}, q_{2,3}, q_{2,4}, q_{2,5}, q_{2,6}, q_{2,7}, q_{3,3}, q_{3,4}, q_{3,5}, q_{3,6}, q_{3,7}, \rho_1, \rho_2, \rho_3, \rho_4, \rho_5, \rho_6, \rho_7, \rho_8, \lambda_1, \lambda_2, \lambda_3, \lambda_4, \lambda_5, \lambda_6, \lambda_7, \sigma_N\rangle$ :

> $Di2 := Dmat(kappai2, parsi2)$ :

> $r := Rank(Di2); d := Dimension(parsi2) - r;$

$$r := 55$$
$$d := 0 \quad \textbf{(21)}$$

>

> #All four data sets

> #Obviously by remark 1 as adult capture-recapture data and,chick MRR data and state−space data are full rank and productivity data is full rank

>
