# Supplementary material for: Parameter redundancy in discrete state‐space and integrated models
Source: Biom J. 2016 Jun 30;58(5):1071–90. doi: 10.1002/bimj.201400239 (PMC5031231; doi:10.1002/bimj.201400239)
Supplement: Supplementary file 2 — Code [file BIMJ-58-1071-s002.zip › Example7.pdf]

```
> #Example 7 of Parameter Redundancy in Discrete State-Space and Integrated Models by D. J.
      Cole and R.S. McCrea
> restart;
> with(LinearAlgebra) :
> Dmat :=proc(kappa, pars)
local DD1, i, j;
description "Form the derivative matrix";
with(LinearAlgebra) :
DD1 := Matrix(1 ..Dimension(pars), 1 ..Dimension(kappa) ) :
for i from 1 to Dimension(pars)  do
    for j from 1 to Dimension(kappa) do
        DD1[i, j] := diff (kappa[j], pars[i])
    end do
 end do;
DD1;
end proc:
> Matvec :=proc(P)
 local sizekappa, i, j, kappa, kappaindex;
description "Converts a Matrix into a Vector of the matrix's non-zero enteries";
with(LinearAlgebra) :  sizekappa := 0 :
for i from 1 to Dimension(P)[1] do
    for j from 1 to Dimension(P)[2] do
        if (P[i, j] ≠ 0) then  sizekappa := sizekappa + 1 :  end if:
    end do
 end do;
κ := Vector(sizekappa) : kappaindex := 0 :
for i from 1 to Dimension(P)[1] do
    for j from 1 to Dimension(P)[2] do
        if (P[i, j] ≠ 0) then
           kappaindex :=   kappaindex + 1 : κ[kappaindex] := P[i, j] :
        end if:
    end do:
 end do:  κ;
 end proc:
> logvector :=proc(A)
local i, lnA;
description "Finds ln of the entries of vector";
with(LinearAlgebra) :
 lnA := Vector(A);
for i from 1 to Dimension(A) do
    lnA[i] := ln(A[i]) :
 end do:
 lnA;
end proc:
> Estpars :=proc(DD1, pars)
local r, d, alphapre, alpha, PDE, FF, i, ans;
description "Finds the estimable set of parameters";
with(LinearAlgebra) :
r := Rank(DD1);
d := Dimension(pars) - r :
```

```
alphapre := NullSpace(Transpose(DD1)) :
α := Matrix(d, Dimension(pars)) : PDE := Vector(d) :
FF := f(seq(pars[i], i = 1 ..Dimension(pars))) :
for i from 1 to d do
      α[i, 1 ..Dimension(pars)] := alphapre[i] :
      PDE[i] := add(diff(FF, pars[j])·α[i, j], j = 1 ..Dimension(pars)) :
 end do:
 ans := pdsolve({seq(PDE[i] = 0, i = 1 ..d)});
 end proc:
> ringmod2 :=proc(y, z, r, c)
 local i, j, P, aa, b;
description "Finds P-array for y/z ring-recovery models.  y survival, z reporting probability. 1=
      constant(C), 2=time(T), 3=age(A), 4=A,T ";
with(LinearAlgebra) :
P := Matrix(r, c) :
 if y = 1 then
     for i from 1 to c do
        for j from 1 to c do
              aa[i, j] := phi :
        end do:
     end do:
 elif y = 2 then
      for i from 1 to c do
        for j from 1 to c do
              aa[i, j] := phi[j] :
        end do:
     end do:
 elif y = 3 then
      for i from 1 to c do
        for j from 1 to c do
              aa[i, j] := phi[i] :
        end do:
     end do:
 else
     for i from 1 to c do
        for j from 1 to c do
              aa[i, j] := phi[i, j] :
        end do:
     end do:
 end if:
  if z = 1 then
     for i from 1 to c do
        for j from 1 to c do
              b[i, j] := lambda :
        end do:
     end do:
 elif z = 2 then
      for i from 1 to c do
        for j from 1 to c do
              b[i, j] := lambda[j] :
        end do:
```

```
        end do:
    elif z = 3 then
         for i from 1 to c do
            for j from 1 to c do
                  b[i, j] := lambda[i] :
            end do:
        end do:
   else
        for i from 1 to c do
            for j from 1 to c do
                  b[i, j] := lambda[i, j] :
            end do:
        end do:
    end if:

    for i from 1 to Dimension(P)[1] do
        for j from i to Dimension(P)[2] do
              P[i, j] := product(aa[k - i + 1, k], k = i..j - 1) · (1 - aa[j - i + 1, j]) · b[j - i + 1,
        j];
        end do:
    end do:
    P;
    end proc:
> #Method A
> # The birds are juvenile for 1 year (J=1) there are 3 year of ringing (n1=3) and 3 years of
      recovery (n2=3):
> J := 1 : n1 := 3 : n2 := 3 :
> # The probabilities of recovery for the juveniles are:
> P := eval(ringmod2(3, 3, n1, n2), {seq(phi[i] = phi[a], i = J + 1 ..10), seq(λ_i = λ_a, i = J + 1
      ..30)});
```

$$P := \begin{bmatrix} (1-\phi_1)\,\lambda_1 & \phi_1\,(1-\phi_a)\,\lambda_a & \phi_1\,\phi_a\,(1-\phi_a)\,\lambda_a \\ 0 & (1-\phi_1)\,\lambda_1 & \phi_1\,(1-\phi_a)\,\lambda_a \\ 0 & 0 & (1-\phi_1)\,\lambda_1 \end{bmatrix} \tag{1}$$

```
> #The exhuastive summary κ_1 is :
> kappa1 := logvector(Matvec(P));
```

(2)

$$\kappa 1 := \begin{bmatrix} \ln\left(\left(1-\phi_1\right)\lambda_1\right) \\ \ln\left(\phi_1\left(1-\phi_a\right)\lambda_a\right) \\ \ln\left(\phi_1\phi_a\left(1-\phi_a\right)\lambda_a\right) \\ \ln\left(\left(1-\phi_1\right)\lambda_1\right) \\ \ln\left(\phi_1\left(1-\phi_a\right)\lambda_a\right) \\ \ln\left(\left(1-\phi_1\right)\lambda_1\right) \end{bmatrix} \quad \textbf{(2)}$$

> # The probabilities of recovery for the adults are:

> $P2 := eval\left(ringmod2(3, 1, n1, n2), \left\{\text{lambda} = \lambda_a, seq(\text{phi}[i] = \text{phi}[a], i = 1..10)\right\}\right);$

$$P2 := \begin{bmatrix} \left(1-\phi_a\right)\lambda_a & \phi_a\left(1-\phi_a\right)\lambda_a & \phi_a^2\left(1-\phi_a\right)\lambda_a \\ 0 & \left(1-\phi_a\right)\lambda_a & \phi_a\left(1-\phi_a\right)\lambda_a \\ 0 & 0 & \left(1-\phi_a\right)\lambda_a \end{bmatrix} \quad \textbf{(3)}$$

> #The exhuastive summary κ_2 is :

> $kappa2 := logvector(Matvec(P2));$

$$\kappa 2 := \begin{bmatrix} \ln\left(\left(1-\phi_a\right)\lambda_a\right) \\ \ln\left(\phi_a\left(1-\phi_a\right)\lambda_a\right) \\ \ln\left(\phi_a^2\left(1-\phi_a\right)\lambda_a\right) \\ \ln\left(\left(1-\phi_a\right)\lambda_a\right) \\ \ln\left(\phi_a\left(1-\phi_a\right)\lambda_a\right) \\ \ln\left(\left(1-\phi_a\right)\lambda_a\right) \end{bmatrix} \quad \textbf{(4)}$$

> #The following code finds the joint exhaustive sumamary (kappa) identifies the parameters (pars). Then finds the devivative matrix (D1) and calculates its rank (r) and the model deficency.

> $\text{kappa} := convert(\langle kappa1, kappa2\rangle, Vector):$

> $pars := \langle seq(op(i, indets(Matvec(\langle P, P2\rangle))), i = 1..nops(indets(Matvec(\langle P, P2\rangle))))\rangle :$

> $D1 := Dmat(\text{kappa}, pars) :$

> $r := Rank(D1); d := Dimension(pars) - r;$

$$r := 4$$

$$d := 0 \quad \textbf{(5)}$$

> # The model rank is 4 and the deficency is 0, therefore the model is full rank and it is possible to estimate all the parameters.

> # Adding 1 extra year of ringing or 1 extra year of recovery adds no extra parameters, therefore by a trivual application of the extension theorem (Catchpole and Morgan, 1997) the model

will always be full rank.

>

> #Method B

> $J := 1 : n1 := 3 : n2 := 3 :$

> $P := eval\left(ringmod2(3, 3, \mathrm{n1}, \mathrm{n2}), \left\{seq(\mathrm{phi}[i] = \mathrm{phi}[a], i = \mathrm{J}+1..10), seq\left(\lambda_i = \lambda_a, i = J+1..30\right)\right\}\right) :$

> $kappa1 := logvector(Matvec(\mathrm{P}));$

$$\kappa 1 := \begin{bmatrix} \ln\left(\left(1-\phi_1\right)\lambda_1\right) \\ \ln\left(\phi_1\left(1-\phi_a\right)\lambda_a\right) \\ \ln\left(\phi_1\,\phi_a\left(1-\phi_a\right)\lambda_a\right) \\ \ln\left(\left(1-\phi_1\right)\lambda_1\right) \\ \ln\left(\phi_1\left(1-\phi_a\right)\lambda_a\right) \\ \ln\left(\left(1-\phi_1\right)\lambda_1\right) \end{bmatrix} \quad (6)$$

> $pars := \langle seq(op(i, indets(Matvec(\mathrm{P}))), i = 1..nops(indets(Matvec(\mathrm{P}))))\rangle :$

> $D1 := Dmat(kappa1, pars) :$

> $r := Rank(D1); d := Dimension(pars) - r;$

$$r := 3$$

$$d := 1 \quad (7)$$

> $Estpars(D1, pars)$

$$\left\{f\left(\phi_1, \phi_a, \lambda_1, \lambda_a\right) = \_F1\left(\phi_a, \lambda_1\left(-1+\phi_1\right), \phi_1\,\lambda_a\right)\right\} \quad (8)$$

> #Reparameterise kappa1

> $s := \left\langle \phi_a, \lambda_1\left(1-\phi_1\right), \phi_1\,\lambda_a\right\rangle :$

> #check (should be zero)

> $Dimension(s) - Rank(Dmat(s, pars));$

$$0 \quad (9)$$

> $A := solve(\{seq(s[i] = ss[i], i = 1..Dimension(s))\}, \{seq(pars[i], i = 1..Dimension(pars))\}) :$

> $\kappa 12 := Vector(kappa1) :$
for i **from** 1 **to** $Dimension(kappa1)$ **do**
$\kappa 12[\mathrm{i}] := simplify(\mathrm{applyrule}([seq(op(i, A), i = 1..nops(A))], kappa1[i]));$
end do;

$$\kappa 12_1 := \ln\left(ss_2\right)$$

$$\kappa 12_2 := \ln\left(-\left(-1+ss_1\right)ss_3\right)$$

$$\kappa 12_3 := \ln\left(-ss_1\left(-1+ss_1\right)ss_3\right)$$

$$\kappa 12_4 := \ln\left(ss_2\right)$$

$$\kappa 12_5 := \ln(-(-1 + ss_1)\, ss_3)$$
$$\kappa 12_6 := \ln(ss_2) \qquad \textbf{(10)}$$

> #check should have d=0:

> $parss := \langle seq(ss[i], i = 1..Dimension(s))\rangle : Ds := Dmat(\kappa 12, parss) : r := Rank(Ds); d := Dimension(parss) - r;$

$$r := 3$$
$$d := 0 \qquad \textbf{(11)}$$

> #Reparameterise kappa2

> $P2 := eval\Big(ringmod2(3, 1, n1, n2), \big\{\text{lambda} = \lambda_a, seq(\text{phi}[i] = \text{phi}[a], i = 1..10)\big\}\Big) :$

> $kappa2 := logvector(Matvec(P2));$

$$\kappa 2 := \begin{bmatrix} \ln((1-\phi_a)\,\lambda_a) \\ \ln(\phi_a\,(1-\phi_a)\,\lambda_a) \\ \ln(\phi_a^2\,(1-\phi_a)\,\lambda_a) \\ \ln((1-\phi_a)\,\lambda_a) \\ \ln(\phi_a\,(1-\phi_a)\,\lambda_a) \\ \ln((1-\phi_a)\,\lambda_a) \end{bmatrix} \qquad \textbf{(12)}$$

> $\kappa 22 := Vector(kappa2) :$
> **for** i **from** 1 **to** $Dimension(kappa2)$ **do**
> $\kappa 22[\mathrm{i}] := simplify(\text{applyrule}([seq(op(i, A), i = 1..nops(A))], kappa2[i]));$
> **end do**;

$$\kappa 22_1 := \ln\left(\frac{(-1 + ss_1)\, ss_3\, \lambda_1}{-\lambda_1 + ss_2}\right)$$
$$\kappa 22_2 := \ln\left(\frac{ss_1\,(-1 + ss_1)\, ss_3\, \lambda_1}{-\lambda_1 + ss_2}\right)$$
$$\kappa 22_3 := \ln\left(\frac{ss_1^2\,(-1 + ss_1)\, ss_3\, \lambda_1}{-\lambda_1 + ss_2}\right)$$
$$\kappa 22_4 := \ln\left(\frac{(-1 + ss_1)\, ss_3\, \lambda_1}{-\lambda_1 + ss_2}\right)$$
$$\kappa 22_5 := \ln\left(\frac{ss_1\,(-1 + ss_1)\, ss_3\, \lambda_1}{-\lambda_1 + ss_2}\right)$$
$$\kappa 22_6 := \ln\left(\frac{(-1 + ss_1)\, ss_3\, \lambda_1}{-\lambda_1 + ss_2}\right) \qquad \textbf{(13)}$$

> # Only 1 extra parameter λ_1 so by remark 3 model is trivually full rank.

> # Adding 1 extra year of ringing or 1 extra year of recovery adds no extra parameters. The estimable parameters of κ_1 remain the same.

>
