# Supplementary material for: Parameter redundancy in discrete state‐space and integrated models
Source: Biom J. 2016 Jun 30;58(5):1071–90. doi: 10.1002/bimj.201400239 (PMC5031231; doi:10.1002/bimj.201400239)
Supplement: Supplementary file 2 — Code [file BIMJ-58-1071-s002.zip › Example6.pdf]

```
> #Example 6 of Parameter Redundancy in Discrete State-Space and Integrated Models by D. J.
      Cole and R.S. McCrea
> restart;
> with(LinearAlgebra) :
> Dmat :=proc(kappa, pars)
  local DD1, i, j;
  description "Form the derivative matrix";
  with(LinearAlgebra) :
  DD1 := Matrix(1 ..Dimension(pars), 1 ..Dimension(kappa)) :
  for i from 1 to Dimension(pars)  do
      for j from 1 to Dimension(kappa) do
          DD1[i, j] := diff(kappa[j], pars[i])
      end do
   end do;
  DD1;
  end proc:
> Matvec :=proc(P)
   local sizekappa, i, j, kappa, kappaindex;
  description "Converts a Matrix into a Vector of the matrix's non-zero enteries";
  with(LinearAlgebra) :  sizekappa := 0 :
  for i from 1 to Dimension(P)[1] do
      for j from 1 to Dimension(P)[2] do
          if (P[i, j] ≠ 0) then  sizekappa := sizekappa + 1 :  end if:
      end do
   end do;
  κ := Vector(sizekappa) : kappaindex := 0 :
  for i from 1 to Dimension(P)[1] do
      for j from 1 to Dimension(P)[2] do
          if (P[i, j] ≠ 0) then
              kappaindex :=   kappaindex + 1 : κ[kappaindex] := P[i, j] :
          end if:
      end do:
   end do:  κ;
   end proc:
> logvector :=proc(A)
  local i, lnA;
  description "Finds ln of the entries of vector";
  with(LinearAlgebra) :
   lnA := Vector(A);
  for i from 1 to Dimension(A) do
      lnA[i] := ln(A[i]) :
   end do:
   lnA;
  end proc:
> Estpars :=proc(DD1, pars)
  local r, d, alphapre, alpha, PDE, FF, i, ans;
  description "Finds the estimable set of parameters";
  with(LinearAlgebra) :
  r := Rank(DD1);
  d := Dimension(pars) - r :
```

```
alphapre := NullSpace(Transpose(DD1)) :
α := Matrix(d, Dimension(pars)) : PDE := Vector(d) :
FF := f(seq(pars[i], i = 1 ..Dimension(pars))) :
for i from 1 to d do
        α[i, 1 ..Dimension(pars)] := alphapre[i] :
        PDE[i] := add(diff(FF, pars[j])·α[i, j], j = 1 ..Dimension(pars)) :
 end do:
 ans := pdsolve({seq(PDE[i] = 0, i = 1 ..d)});
 end proc:
> ringmod2 :=proc(y, z, r, c)
 local i, j, P, aa, b;
description "Finds P-array for y/z ring-recovery models.  y survival, z reporting probability. 1=
      constant(C), 2=time(T), 3=age(A), 4=A,T ";
with(LinearAlgebra) :
P := Matrix(r, c) :
 if y = 1 then
     for i from 1 to c do
        for j from 1 to c do
              aa[i, j] := phi :
        end do:
     end do:
 elif y = 2 then
      for i from 1 to c do
        for j from 1 to c do
              aa[i, j] := phi[j] :
        end do:
     end do:
 elif y = 3 then
      for i from 1 to c do
        for j from 1 to c do
              aa[i, j] := phi[i] :
        end do:
     end do:
 else
     for i from 1 to c do
        for j from 1 to c do
              aa[i, j] := phi[i, j] :
        end do:
     end do:
 end if:
  if z = 1 then
     for i from 1 to c do
        for j from 1 to c do
              b[i, j] := lambda :
        end do:
     end do:
 elif z = 2 then
      for i from 1 to c do
        for j from 1 to c do
              b[i, j] := lambda[j] :
        end do:
```

```
        end do:
    elif z = 3 then
         for i from 1 to c do
            for j from 1 to c do
                  b[i, j] := lambda[i] :
            end do:
        end do:
    else
        for i from 1 to c do
            for j from 1 to c do
                  b[i, j] := lambda[i, j] :
            end do:
        end do:
    end if:

    for i from 1 to Dimension(P)[1] do
        for j from i to Dimension(P)[2] do
            P[i, j] := product(aa[k − i + 1, k], k = i ..j − 1) · (1 − aa[j − i + 1, j]) · b[j − i + 1,
        j];
        end do:
    end do:
    P;
    end proc:
```

```
> #Ringrecovery Model
> J := 2 : P := eval(ringmod2(4, 1, 4, 4), {seq(seq(phi[i, j] = phi[a, ], i = J + 1 ..10), j = 1
    ..20), seq(seq(phi[i, j] = phi[i], i = 1 ..J), j = 1 ..20)})
```

$$P := \begin{bmatrix} (1-\phi_1)\,\lambda & \phi_1\,(1-\phi_2)\,\lambda & \phi_1\,\phi_2\,(1-\phi_a)\,\lambda & \phi_1\,\phi_2\,\phi_a\,(1-\phi_a)\,\lambda \\ 0 & (1-\phi_1)\,\lambda & \phi_1\,(1-\phi_2)\,\lambda & \phi_1\,\phi_2\,(1-\phi_a)\,\lambda \\ 0 & 0 & (1-\phi_1)\,\lambda & \phi_1\,(1-\phi_2)\,\lambda \\ 0 & 0 & 0 & (1-\phi_1)\,\lambda \end{bmatrix} \tag{1}$$

```
> kappa := Matvec(P) :
> pars := ⟨seq(op(i, indets(kappa)), i = 1 ..nops(indets(kappa)))⟩ :
> D1 := Dmat(kappa, pars) :
> r := Rank(D1); d := Dimension(pars) − r;
```

$$r := 4$$

$$d := 0 \tag{2}$$

```
> #Ring-recovery model is full rank. Add an extra year of ringing or recovery adds no extra
    parameters therefore by a trivual application of the extension theorem (Catchpole and
    Morgan, 1997) this model will always be full rank.
> #Capture recapture model
> P := ⟨⟨phi[a]·p[2]|phi[a]^2·(1 − p[2])·p[3]|phi[a]^3·(1 − p[2])·(1 − p[3])·p[4]|phi[a]^4
    ·(1 − p[2])·(1 − p[3])·(1 − p[4])·p[5]⟩, ⟨0|phi[a]·p[3]|phi[a]^2·(1 − p[3])·p[4]
    |phi[a]^3·(1 − p[3])·(1 − p[4])·p[5]⟩, ⟨0|0|phi[a]·p[4]|phi[a]^2·(1 − p[4])·p[5]⟩, ⟨0|0
    |0|phi[a]·p[5]⟩⟩
```

$$P := \begin{bmatrix} \phi_a p_2 & \phi_a^2 (1-p_2) p_3 & \phi_a^3 (1-p_2)(1-p_3) p_4 & \phi_a^4 (1-p_2)(1-p_3)(1-p_4) p_5 \\ 0 & \phi_a p_3 & \phi_a^2 (1-p_3) p_4 & \phi_a^3 (1-p_3)(1-p_4) p_5 \\ 0 & 0 & \phi_a p_4 & \phi_a^2 (1-p_4) p_5 \\ 0 & 0 & 0 & \phi_a p_5 \end{bmatrix} \quad \textbf{(3)}$$

> kappa $:= Matvec(P)$:

> $pars := \langle seq(op(i, indets(\text{kappa})), i = 1..nops(indets(\text{kappa})))\rangle$:

> $D1 := Dmat(\text{kappa}, pars)$:

> $r := Rank(D1); d := Dimension(pars) - r;$

$$r := 5$$

$$d := 0 \quad \textbf{(4)}$$

> #Capture-recapture model is full rank. Add an extra year of recapture only adds adds one extra parameter extra parameters, adding an extra year of marking adds no extra parameters, therefore by a trivual application of the extension theorem (Catchpole and Morgan, 1997) this model will always be full rank.

> # As both models are individually full rank by remark 1 the joint model is also full rank.

>
